# The Diversity of Parasitoids and Their Role in the Control of the Siberian Moth, *Dendrolimus sibiricus* (Lepidoptera: Lasiocampidae), a Major Coniferous Pest in Northern Asia

**DOI:** 10.3390/life14020268

**Published:** 2024-02-17

**Authors:** Natalia I. Kirichenko, Alexander A. Ageev, Sergey A. Astapenko, Anna N. Golovina, Dmitry R. Kasparyan, Oksana V. Kosheleva, Alexander V. Timokhov, Ekaterina V. Tselikh, Evgeny V. Zakharov, Dmitrii L. Musolin, Sergey A. Belokobylskij

**Affiliations:** 1 Federal Research Center, Sukachev Institute of Forest, Siberian Branch of the Russian Academy of Sciences, Akademgorodok 50/28, 660036 Krasnoyarsk, Russia; 2 Institute of Ecology and Geography, Siberian Federal University, Svobodny pr. 79, 660041 Krasnoyarsk, Russia; 3 All-Russian Plant Quarantine Center (FGBU VNIIKR), Krasnoyarsk Branch, Zhelyabova Str., 6/6, 660020 Krasnoyarsk, Russia; 4 All-Russian Research Institute of Forestry and Forestry Mechanization (VNIILM), “Forest Pyrology Center”, Krasnoyarsk Branch, Krupskoy St., 42, 660062 Krasnoyarsk, Russia; ageevaa@firescience.ru (A.A.A.); forest_les@mail.ru (S.A.A.); golovinaan@firescience.ru (A.N.G.); 5 Federal Budgetary Institution “Russian Forest Protection Center”, Akademgorodok 50/2, 660036 Krasnoyarsk, Russia; 6 Zoological Institute of the Russian Academy of Sciences, Universitetskaya nab. 1, 199034 Saint Petersburg, Russia; kasparyan@yandex.ru (D.R.K.); tselikhk@gmail.com (E.V.T.); 7 All-Russian Institute of Plant Protection (FSBSI VIZR), Podbelskogo 3, 196608 Saint Petersburg, Russia; koscheleva_o@mail.ru; 8 Department of Entomology, Lomonosov Moscow State University, Leninskie Gory, 119234 Moscow, Russia; atimokhov@mail.ru; 9 Canadian Center for DNA Barcoding, Centre for Biodiversity Genomics, College of Biological Sciences, University of Guelph, 50 Stone Road, Guelph, ON N1G 2W1, Canada; zakharov@uoguelph.ca; 10 European and Mediterranean Plant Protection Organization, 21 Boulevard Richard Lenoir, 75011 Paris, France; musolin@gmail.com

**Keywords:** Hymenoptera, Diptera, Lasiocampidae, Siberia, Asia, archival specimens, morphological identification, DNA barcoding, check list, parasitism

## Abstract

The Siberian moth, *Dendrolimus sibiricus* Tschetv., 1908 (Lepidoptera: Lasiocampidae) is a conifer pest that causes unprecedented forest mortality in Northern Asia, leading to enormous ecological and economic losses. This is the first study summarizing data on the parasitoid diversity and parasitism of this pest over the last 118 years (1905–2022). Based on 860 specimens of freshly reared and archival parasitoids, 16 species from two orders (Hymenoptera and Diptera) were identified morphologically and/or with the use of DNA barcoding. For all of them, data on distribution and hosts and images of parasitoid adults are provided. Among them, the braconid species, *Meteorus versicolor* (Wesmael, 1835), was documented as a parasitoid of *D. sibiricus* for the first time. The eastern Palaearctic form, *Aleiodes esenbeckii* (Hartig, 1838) *dendrolimi* (Matsumura, 1926), status nov., was resurrected from synonymy as a valid subspecies, and a key for its differentiation from the western Palaearctic subspecies *Aleiodes esenbeckii* ssp. *esenbecki* is provided. DNA barcodes of 11 parasitoid species from Siberia, i.e., nine hymenopterans and two dipterans, represented novel records and can be used for accurate molecular genetic identification of species. An exhaustive checklist of parasitoids accounting for 93 species associated with *D. sibirisus* in northern Asia was compiled. Finally, the literature and original data on parasitism in *D. sibiricus* populations for the last 83 years (1940–2022) were analysed taking into account the pest population dynamics (i.e., growth, outbreak, decline, and depression phases). A gradual time-lagged increase in egg and pupal parasitism in *D. sibiricus* populations was detected, with a peak in the pest decline phase. According to long-term observations, the following species are able to cause significant mortality of *D. sibiricus* in Northern Asia: the hymenopteran egg parasitoids *Telenomus tetratomus* and *Ooencyrtus pinicolus*; the larval parasitoids *Aleiodes esenbeckii* sp. *dendrolimi*, *Cotesia* spp., and *Glyptapanteles liparidis*; and the dipteran pupal parasitoids *Masicera sphingivora*, *Tachina* sp., and *Blepharipa* sp. Their potential should be further explored in order to develop biocontrol programs for this important forest pest.

## 1. Introduction

The Siberian moth, *Dendrolimus sibiricus* Tschetv., 1908 (Lepidoptera: Lasiocampidae), is one of the major pests of taiga forests in Northern Asia, damaging coniferous species of *Abies*, *Larix*, *Picea*, and *Pinus* (Pinaceae) [1,2,3]. Its outbreaks can occur simultaneously on large territory covering millions of hectares [1,2,3,4,5,6,7]. The dramatic damage caused by *D. sibiricus* to forests makes it one of the main biotic factors determining the succession trends and structural composition of taiga forests [3,8,9]. In addition to the Asian part, the species is present in the European part of Russia, where it also damages coniferous stands significantly [10]. Given the potential risks associated with the introduction of this pest into European countries, it has been included in the A2 quarantine list of EPPO (European and Mediterranean Plant Protection Organization), and stringent regulations are enforced to prevent its inadvertent introduction [11].

In its native range, the most recent pest outbreaks occurred between 2011 and 2022 in Krasnoyarsk Territory, Kemerovo, and Tomsk Provinces (Siberia, Russia), affecting an area of over 1.5 million ha of forests and causing economic losses (mostly wood losses) of RUB 127.8 billion [12], i.e., appr. USD 2.1 billion (based on the currency rate in December 2022) [13]. Since the late 19th century, in Krasnoyarsk Territory alone, nine outbreaks were documented [14], each time resulting in profound ecological and economic consequences. For instance, the outbreak of 1952–1958 led to the destruction of forests, resulting in the loss of timber stock estimated at a staggering 424 million m^3^ [15].

Based on the damage associated with wood loss, the expenses incurred for controlling the pest (mostly insecticide treatments using aviation) are considerable. For instance, in the late 1990s in Krasnoyarsk Territory, the pest control costs in the area of 480 thousand ha exceeded non-denominated RUB 34 billion [16], i.e., appr. USD 1.9 mln, taking into account the monetary reform in Russia in 1998 [17] and the currency rate of December 1998) [13]. In the last three years (2021–2023), the outbreaks of *D. sibiricus* have been documented in other Siberian regions: Irkutsk Province, the Republic of Buryatia, the Republic of Sakha (or Yakutia), and Altai Territory, and the economic and ecological losses are already discernible, though they have yet to reach their peak.

*D. sibiricus* outbreaks are triggered by hot weather and water deficit during the vegetation season and occur with a periodicity of 10–12 years [3]. The life cycle usually takes 2–3 years, with overwintering in larval stage [1,2,3]. However, in built-up populations and at favorable climatic conditions, *D. sibiricus* may switch to a shorter, 1-year life cycle, whereas during the depression phase, the life cycle may last up to 5 years [3,18]. The adults of *D. sibiricus* do not feed; they emerge in early July to early August (Prozorov, 1952). Females lay eggs in bunches (up to 200 eggs) on needles or twigs within the crown of a host plant [3]. The fecundity is 200–300 eggs, rarely up to 800 eggs (in the individuals developed on larch *Larix sibirica*) [3,18]. Under natural conditions, eggs develop for 2–3 weeks, and larvae pass six instars (exceptionally, seven instars) within 1–3 years [3]. Larvae of each subsequent instar develop longer consuming more needles and causing more damage to the trees [3,18]. Pupation occurs in thick cocoons attached to twigs or branches.

The diversity of natural enemies of *D. sibiricus*, in particular, the parasitoid fauna, remains understudied in Northern Asia. The majority of studies on the parasitoids were performed in Siberia in the 1950s–1960s [18,19,20,21]. The parasitoid complex primarily comprises Hymenoptera species that target various developmental stages of *D. sibiricus* (egg, larva, and pupa) [19]. Additionally, a few tachinid species (Diptera) are also known to parasitize *D. sibiricus* [22]. In Siberia, the composition of parasitoid complexes varies greatly and depends on the region [18,23,24]. The following species are generally known to be most abundant in the pest range: the hymenopteran egg parasitoid *Telenomus tetratomus* (Thomson) (Scelionidae), the larval parasitoid *Aleiodes esenbeckii* (Hartig) (=*Rhogas dendrolimi* Matsumura) (Braconidae), and the pupal parasitoid *Masicera sphingivora* (Robineau-Desvoidy) (=*M. zimini* Kolomiets) (Diptera: Tachinidae) [18,19,20,21,24,25,26,27].

Here, we summarized long-term data of the diversity of parasitoids associated with *D. sibiricus* in Northern Asia. Using an integrative approach combining morphological identification and DNA barcoding, we identified parasitoids reared from the eggs, larvae, and pupae of *D. sibiricus* from different Siberian regions and those sampled in the 20th century and stored in the collections of national research institutes. We compiled a comprehensive checklist of parasitoids associated with *D. sibiricus*, unveiling novel trophic associations. Subsequently, we examined parasitism data across various life stages of *D. sibiricus* (eggs, larvae, and pupae) in relation to the pest’s population dynamics, which encompassed growth, outbreak, decline, and depression phases. Furthermore, we estimated the contribution of some parasitoid species to the pest’s mortality in Northern Asia.

## 2. Materials and Methods

### 2.1. Study Region

**Figure 1:**
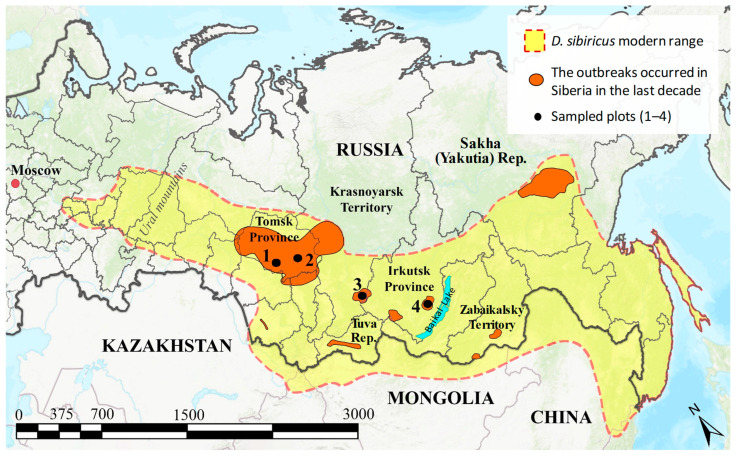
Modern range of *Dendrolimus sibiricus*, the most recent outbreaks and sampled sites in Northern Asia. The pest’s range is modified from Rhozkov [3] and Kononov et al. [28]. Rep.—Republic. The map was generated using ArcGIS 9.3 [29].

This study was carried out in Northern Asia (herein referred to as the Asian part of Russia, i.e., Siberia and the Russian Far East), in three administrative regions: Krasnoyarsk Territory and Tomsk and Irkutsk Provinces, where *D. sibiricus* outbreaks occurred in 2014–2022 (Figure 1).

In Krasnoyarsk Territory, the outbreak happened over an area of 123.4 thousand ha at the foothills of the Eastern Sayan Mountains in a coniferous forest dominated by *A. sibirica* Ledeb. and *Pinus sibirica* Du Tour. in 2018–2022. In Tomsk Province, it covered an area of 808.2 thousand ha in the eastern part of the West Siberian Plain, in dense coniferous forests primarily composed of *Abies sibirica*, and lasted from 2015 to 2018. In Irkutsk Province, it occurred over an area of 11.2 thousand ha in the southern foothills of Baikal Lake, in mixed tree stands with a prevalence of *Larix sibirica* Ledeb. in 2014–2022.

**Figure 2:**
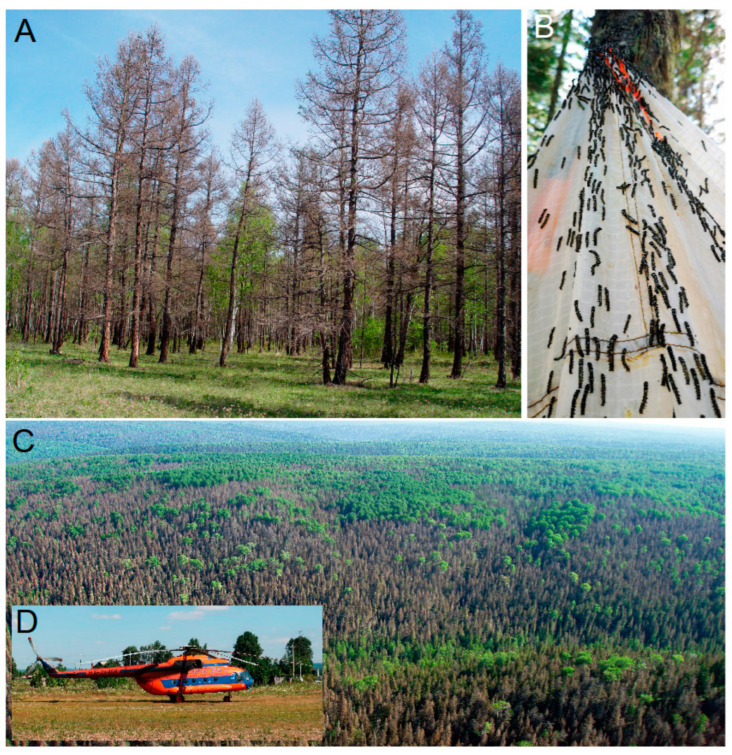
Sampled foci of *Dendrolimus sibiricus* in Siberia in 2018–2022. (**A**) Significantly defoliated fir forest, Kachug (Irkutsk Province), June 2021; (**B**) sampling of larvae emerging from litter, Irbei (Krasnoyarsk Territory), end of April 2019; (**C**) aerial view of the foci in the mixed forest predominated by fir, *Abies sibirica*, Tomsk Province, July 2018; and (**D**) the helicopter used for accessing infested plots. Photos: A.A. Ageev, S.A. Astapenko.

Overall, four plots (1 × 1 km each) were sampled: one in Krasnoyarsk Territory in March–August of 2019–2021, two in Tomsk Province in July–September 2018, and one in Irkutsk Province in May–August of 2021–2022. Some of these localities were accessible only with a helicopter (Figure 2). The visiting period coincided with the decline in *D. sibiricus* populations, which increased the probability of collecting the pest eggs, larvae, and pupae infested by parasitoids.

### 2.2. Field Sampling

**Figure 3:**
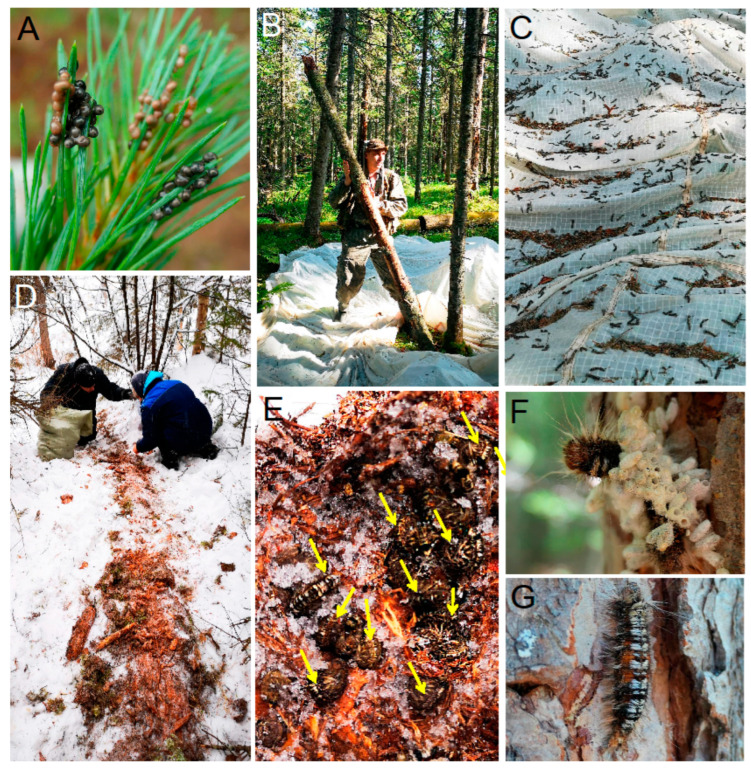
Sampling of *Dendrolimus sibiricus* specimens in the forest: (**A**) eggs, July 2022; (**B**,**C**) collecting young larvae by beating tree stems, August 2019; (**D**,**E**) collecting overwintering late instar larvae (shown by arrows) from the litter, January 2020; and (**F**,**G**) collecting parasitized late instar larvae found on tree stems, June 2022. Photos: A.A. Ageev, S.A. Astapenko. Images B and D are published with permission from the photographed coauthors (S.A. Astapenko and A.N. Golovina).

Eggs, larvae, and pupae of *D. sibiricus* were collected in outbreaking localities to obtain the parasitoids associated with the pest’s eggs, larvae and pupae. Clusters of eggs laid by females on needles were sampled with needles and twigs during July, i.e., a few days after oviposition so that the parasitoids had enough time to infest the eggs (Figure 3A).

Young larvae (I–III instars) were collected in August–September by beating tree trunks (Figure 3B,C). Overwintering larvae (III and IV instars) were collected in January 2020 from the leaf litter beneath the snow (Figure 3D,E). Late instar larvae (IV–VI) found on tree branches (eventually, parasitized larvae found on tree stems) were collected in May–June (Figure 3F,G). The cocoons with pupae found on branches and trunks of coniferous trees were sampled in late June–early July. Overall, across all visited sites, about 300 egg clusters (each containing from 40 to 300 eggs), 3000 early and late instar larvae, and 1000 pupae were collected. They were transported to Krasnoyarsk for indoor rearing.

### 2.3. Rearing Parasitoids

**Figure 4:**
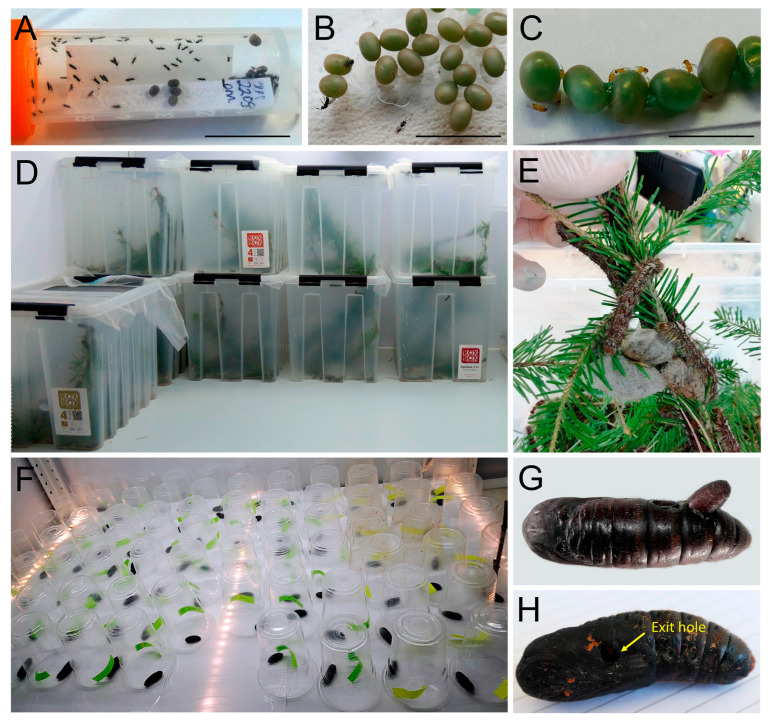
Obtaining parasitoids from different ontogenetic stages of *Dendrolimus sibiricus* in laboratory conditions: (**A**) parasitoids of *D. sibiricus* eggs; (**B**,**C**) parasitoids exploring and infesting fresh eggs of *D. sibiricus*; (**D**,**E**) rearing larvae of *D. sibiricus* and sampling the parasitized specimens; and (**F**–**H**) keeping *D. sibiricus* pupae for monitoring parasitoids emergence. Photos: A.A. Ageev.

The collected eggs, larvae, and pupae of *D. sibiricus* were maintained in the laboratory under stable conditions: 24 ± 2 °C air temperature, 50% relative humidity, and a 16:8 h (day/night) photoperiod for obtaining the parasitoids. The eggs from different clusters were pooled together according to the sampled locality, placed in Petri dishes with about 100–200 eggs per dish, and kept for 2–5 days for maturing. After maturing, parasitized eggs were selected based on the change in the chorion colour (from light brown to dark grey in the case of parasitized eggs vs. from light brown to dark brown in the case of not parasitized eggs). Parasitized eggs were transferred to 1.5 mL tubes (10–30 eggs per tube) sealed with cotton and kept until parasitoids emerged (Figure 4). The tubes were monitored daily.

Early instar larvae (I–III instars) were kept in Petri dishes (90 mm diameter) with 50 individuals per dish; late instar larvae (IV–VI) were kept in plastic containers (4000 mL) with 50 individuals per container, sealed by mesh, and lined with filter paper to absorb excessive humidity. Larvae were provided with 2–4-year-old shoots of the main host plant species, *Abies sibirica* and *Larix sibirica*, for feeding. Tree shoots were replaced regularly (daily or every second day) with fresh shoots, frass was removed, and the dishes and containers were cleaned by wiping tissues moistened with ethanol solution (95%) to avoid mould.

Pupae obtained through rearing in the laboratory and those collected from forests were visually checked, and those that looked unhealthy (i.e., were soft, had weakly or non-moving abdomen) were individually placed in plastic glasses (200 mL) and kept until parasitoid emergence. The glasses with pupae were monitored daily.

Overall, 630 specimens of parasitoids were obtained and used for morphological identification. The emerged adults of egg parasitoids were preserved in 95% ethanol solution in 1.5 mL tubes; those that emerged from larvae and pupae of *D. sibiricus* were pinned, and the parasitoid specimens from the same series were preserved in 95% ethanol solution and stored in a freezer at −20 °C for morphological and molecular genetic studies.

### 2.4. Involvement of Archival Specimens

In the past, parasitoids obtained by earlier researchers from the eggs, larvae, or pupae of *D. sibiricus* were sent to specialists at the Zoological Institute of Russian Academy of Sciences (ZISP, St. Petersburg, Russia), who worked with various Hymenoptera (Ichneumonidae, Braconidae, Scelionidae, and Chalcidoidea) and Diptera (Tachinidae). The specimens were subsequently archived at the collection of ZISP. The Siberian entomologists, Yu.P. Kondakov and N.G. Kolomiets, also deposited some parasitoids reared from *D. sibiricus* to the collection housed within the Forest Zoology laboratory at the Sukachev Institute of Forest Siberian Branch of the Russian Academy of Sciences (SIF, Krasnoyarsk, Russia). The specimens stored in these collections were used in our study.

In total, 230 specimens of parasitoids, collected between 1905 and 1966, and held in the collections of these two institutions, were analysed. Sixty-three parasitoid specimens, reared from different ontogenetic stages of *D. sibiricus*, originated from Siberia: three specimens were from Novosibirsk Province (1962, N. Kolomiets col.), twenty-eight from Tomsk Province (1955, N. Kolomiets col.), four from Krasnoyarsk Territory (1957, Lipanova col.), 7 from Irkutsk Province (1927, Floroff col.; 1949, Bondarev col.), nineteen from Tuva Republic (1958–1965, Yu.P. Kondakov col.; 1958, N. Kolomiets col.), and one specimen from Buryatia Republic (1966, Mikhaylov col.). Fifty-five parasitoid specimens reared from *D. sibiricus* originated from Sakhalin Province (1964; D. Kasparyan col.), two from Korea (unknown date; unknown collector), and one from Mongolia (1905; P. Kozlov col.). The list of studied specimens (including collection data, region, and name of collector) is given for each species below.

### 2.5. DNA Barcoding

A total of 48 parasitoids of *D. sibiricus* were involved in DNA barcoding. Both freshly reared adults (37 specimens) and one larva, and the specimens stored in early collections (10 pinned and morphologically identified adults), were DNA barcoded to identify species, define intra- and interspecific divergence, and, where possible, address taxonomic concerns. Prior to performing DNA barcoding, all adult specimens were identified morphologically. The hymenopteran parasitoids *Iseropus stercorator* (Fabricius, 1793), *Therion circumflexum* (Linnaeus, 1758) (both Ichneumonidae), and *Meteorus versicolor* (Braconidae) were not DNA barcoded as they were included in this study at a later stage.

Whole bodies of parasitoid adults were used (in the case of tiny individuals) or only hindlegs (in large adult specimens). Non-destructive DNA extraction was applied in order to save adult bodies and return them to ZISP and SIF. The mitochondrial cytochrome oxidase I gene (mtDNA COI, 658 bp) was sequenced in parasitoids using the standard protocol [30]. The analyses were done at the Canadian Centre for DNA Barcoding (CCDB, Centre for Biodiversity Genomics, College of Biological Sciences, University of Guelph).

The parasitoid species were identified by their DNA barcodes in BOLD SYSTEM (Barcode of Life Data System, https://www.boldsystems.org/, accessed on 1 January 2024). The nearest neighbors were determined and Barcode Index Numbers (BINs), used as a species proxy identifier in BOLD [31], were retrieved. DNA barcodes of 12 parasitoid species publicly available in BOLD were used for comparison. Specimen data are provided in Table S1. The voucher data, the original DNA sequences and trace files, BINs, and GenBank accession numbers can be retrieved at https://dx.doi.org/10.5883/DS-PARDS (accessed on 1 January 2024).

The sequences were aligned in BioEdit 7.2.5 [32]. The phylogenetic trees were built in MEGA X [33] using the maximum likelihood method, the Kimura two-parameter model, and a bootstrap method (2000 iterations). Where possible, intra- and interspecific genetic distances were assessed using the same approaches. Two DNA barcodes obtained for *Phytomyza* sp. (Diptera: Agromyzidae) and *Profenusa thompsoni* (Konow, 1886) (Hymenoptera: Tenthredinidae), collected by our team in Novosibirsk (Russia) from the leaf mines on *Populus balsafimera* and *Betula pendula*, respectively, were used to root the genetic trees. The Spearman’s rank correlation (R) was utilized to estimate the sequencing success, i.e., the relationship between the length of the sequenced fragment of the gene COI (658 bp lenght) and the age of parasitoid archival specimens. For this, the data on sequence length and the specimen age were pooled together from different parasitoid species.

### 2.6. Identification of Parasitoids on Morphology

The parasitoids were identified mainly based on their external morphological features using the keys for different taxonomic groups [34,35,36,37,38,39,40,41,42,43,44].

Pictures of biotopes and insects in the forest were taken using the digital camera Panasonic DC-TZ200 (Panasonic Corporation, Osaka, Japan); aerial imaging of forest damaged by *D. sibiricus* was performed from the plane Mi-8 (Kazan Helicopters, Joint Stock Company, Kazan, Russia) using the digital camera Canon PowerShot G9 X Mark II (Canon Inc., Tokyo, Japan). The parasitoids were examined for morphological identification using an Olympus SZ51 stereomicroscope (Olympus Corporation, Tokyo, Japan). Photographs were taken with an Olympus OM-D E-M1 digital camera mounted on an Olympus SZX10 microscope (Olympus Corporation, Tokyo, Japan) (ZISP, St Petersburg, Russia). Ultrastructural morphological features of *Telenomus* specimens were examined under a JEOL JSM-6380 scanning electron microscope (SEM) (JEOL Ltd., Tokyo, Japan) after critical point drying Hitachi HCP-2 (Hitachi Ltd., Tokyo, Japan) and sputter coating with gold (Giko JSM-6380). Image stacking was performed using Helicon Focus 8.0 (Kharkiv, Ukraine; https://helicon-focus.software.informer.com/5.0/, accessed on 1 May 2023). The figures were produced using the Photoshop 24.0.1 program.

The studied freshly reared parasitoid specimens are deposited in the collections of the ZISP (St Petersburg), SIF (Krasnoyarsk), and the Zoological Museum of Lomonosov Moscow State University (Moscow, Russia; ZMMU). This studied material was compared with available specimens from the ZISP collection determined by expert taxonomists for corresponding systematic groups. The images of specimens *Telenomus bombycis* (lectotype, female, NHMW-HYM#0005384) and *T. gracilis* (lectotype, male, NHMW-HYM#0005403) stored in the Naturhistorisches Museum Wien (Vienna, Austria) were also studied.

### 2.7. Parasitoid List

For each of the 16 parasitoid species identified in the present study, information on the studied material, data on hosts, and distributions are provided. The new distributional records or trophic associations are marked with an asterisk (*).

An exhaustive checklist of parasitoids of *D. sibiricus* was compiled based on the analysis of the literature sources published in the last 109 years [2,19,20,21,22,23,24,25,26,27,28,36,37,38,39,40,42,43,44,45,46,47,48,49,50,51,52,53,54,55,56,57,58,59,60,61,62,63,64,65,66,67,68,69,70,71,72,73,74,75,76,77,78,79,80,81,82,83,84,85,86,87,88,89,90,91,92,93,94,95,96,97]. It includes the data on parasitoid hosts and specialization.

### 2.8. Parasitism in D. sibiricus Populations

Data on parasitism of eggs, larvae, and pupae in different populations of *D. sibiricus* experiencing growth, outbreak, decline, or depression in Siberia were extracted from sources in the literature ([2,7,14,18,19,20,21,55,98], etc.) from the last 83 years (from 1940 to 2020). They were supplemented by the data that we obtained from outbreak localities of the pest in Krasnoyarsk Territory and Tomsk and Irkutsk Provinces in 2018–2022. In many cases, in addition to absolute values of parasitism, the number of *D. sibiricus* specimens (eggs, larvae, and pupae) was provided in the literature; thus, we were able to estimate relative parasitism in %. The latter was calculated as a ratio of parasitized *D. sibiricus* individuals to the number of examined specimens and provided according to the Siberian moth developmental stages (egg, larva, pupa) and the population dynamics phase (i.e., growth, outbreak, decline, depression) [99] (Table S2). The growth phase is the transition of a population from a stable state (i.e., low population density) to a phase of population increase; the outbreak stage is a continuing increase in the population density and the expansion of outbreak boundaries; the decline stage is a gradual decrease in the population density and the reproduction intensity; and the depression stage is a continuing decline in the population density due to maximum impact of regulatory factors (biotic and/or abiotic) resulting in the minimum population size of a pest [99].

The relative contribution of different parasitoid species is estimated as a percentage of mortality caused by a certain parasitoid species from total parasitism in the populations of *D. sibiricus* experiencing growth, outbreak peak, decline, or depression.

The parasitism values were averaged (±standard error, i.e., SE) for the egg, larval, and pupal stages of *D. sibiricus* according to the growth, outbreak, decline, and depression phases. The Mann–Whitney test was used to compare parasitism between different *D. sibiricus* population phases. A polynomial quadratic function was used to describe the changes in parasitism in different ontogenetic stages of *D. sibiricus* and different population dynamics phases. Furthermore, the data on mortality due to certain parasitoid species was estimated as an average value ± SE (%) to demonstrate the contribution of different parasitoid species to egg, larval, and pupal mortality at different population dynamics phases of *D. sibiricus*.

## 3. Results

### 3.1. Molecular Genetic Data

**Figure 5:**
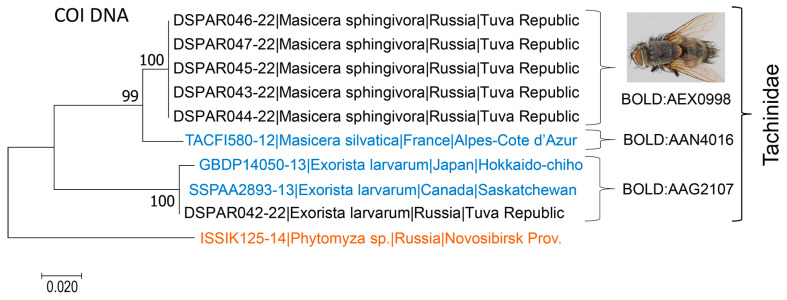
Maximum likelihood tree showing the relatedness of tachinids attacking *Dendrolimus sibiricus* in Northern Asia with closest neighbours in BOLD (borrowed sequences are indicated in blue); the outgroup is indicated in orange. Each specimen is provided with a BOLD process ID (beginning with DSPAR for our data), followed by species name, country, and region. Bootstrap values > 70 are indicated next to the corresponding branches. BIN numbers are given next to each cluster. The adult image is provided for the most frequently recorded tachinid species in Siberia.

**Figure 6:**
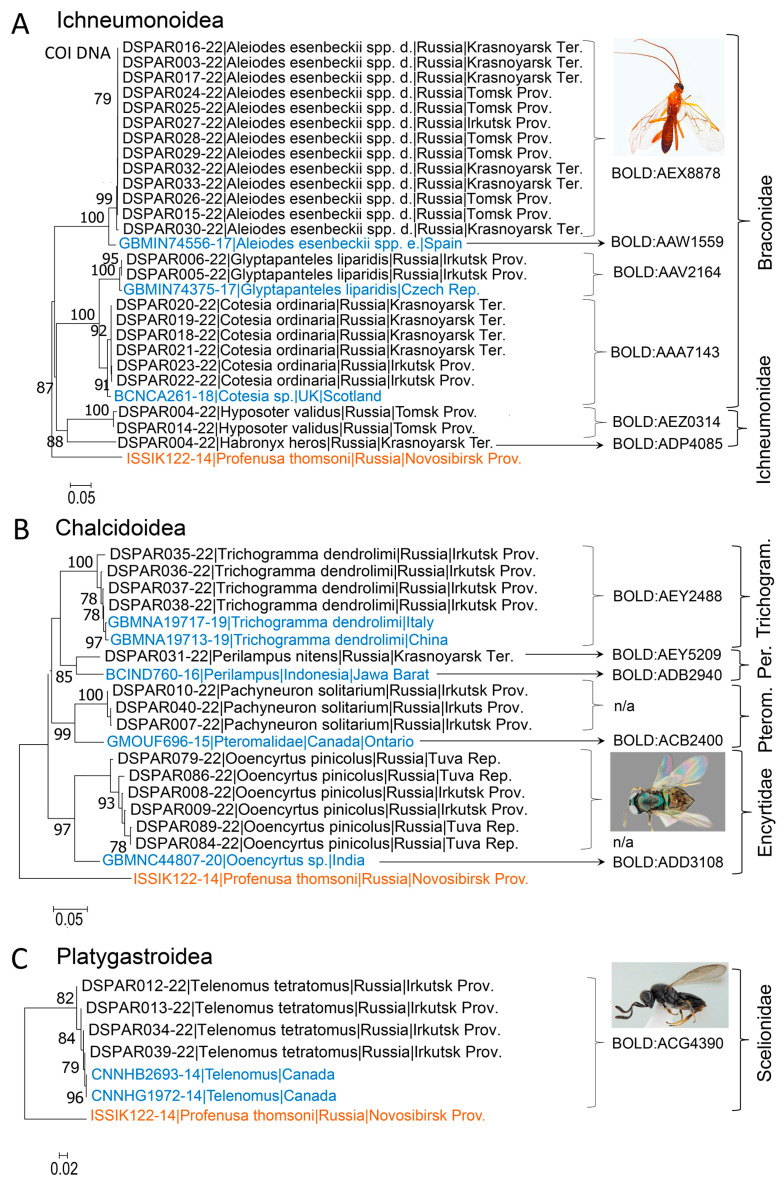
Maximum likelihood trees showing the relatedness of hymenopteran parasitoids from three superfamilies: (**A**) Ichneumonoidea, (**B**) Chalcidoidea, and (**C**) Platygastroidea, attacking *Dendrolimus sibiricus* in Northern Asia, with closest neighbours in BOLD. Borrowed sequences are highlighted in blue; the outgroup is indicated in orange. Each specimen is indicated by a BOLD process number, followed by species name, country, and region. Next to the clusters, BINs and family names are provided. n/a—not assignable (not possible to assign BIN in BOLD as the sequences are short, i.e., <430 bp). Bootstrap values >70 are indicated next to the corresponding branches. The adult images are shown for some frequently recorded species in Siberia. Abbreviation: Province—Province, Ter.—*Territory*, spp. d.—subspecies *dendrolimi*, spp. e.—subspecies *esenbeckii*; families: Trichogram.—Trichogrammatidae, Per.—Perilampidae, Pterom.—Pteromalidae.

Overall, 48 DNA barcodes were obtained from Siberia (Figure 5 and Figure 6). COI sequences of the targeted length (658 b.p.) were obtained for 23 specimens (48%). Eighteen specimens (37%) yielded sequences of length between 430 and 563 bp. Short fragments of the COI gene (207–338 bp) were obtained for seven specimens (15%). No significant correlation was documented between the age of specimens (3–61 years old, 1963–2022) and the success of sequencing, i.e., obtained length of the COI gene: y = 1.87x − 3227, R^2^ = 0.087 (N = 48; *p* > 0.05).

Overall, 48 DNA-barcoded parasitoid specimens were identified as 12 species by morphology. However, DNA barcoding allowed us to reliably identify only 3 out of 12 species (i.e., 23% of all DNA barcoded species in the study) in BOLD and/or GenBank, i.e., the fly *Exorista larvarum* (Linnaeus, 1758) (Tachinidae) and two hymenopteran parasitoids *Glyptapanteles liparidis* (Bouché, 1834) (Braconidae) and *Trichogramma dendrolimi* Matsumura, 1926 (Trichogrammatidae) (Figure 5).

The other 10 species sequenced in our study, with reliable morphological identification, represented novel records for BOLD and GenBank. Among them, one dipteran parasitoid, *Masicera sphingivora* (Robineau-Desvoidy, 1830), and nine hymenopterans: *Habronyx heros* (Wesmael, 1849), *Hyposoter validus* (Pfankuch, 1921) (both Ichneumonidae), *Aleiodes esenbeckii* (Hartig, 1838) ssp. *dendrolimi* (Matsumura, 1926), status nov., *Cotesia ordinaria* (Ratzeburg, 1844) (both Braconidae), *Perilampus nitens* Walker, 1834 (Perilampidae), *Pachyneuron solitarium* (Hartig, 1838) (Pteromalidae), *Ooencyrtus pinicolus* (Matsumura, 1926) (Encyrtidae), and *Telenomus tetratomus* (Thomson, 1861) (Scelionidae) were identified.

In BOLD or Genbank, the hymenopteran parasitoids from Europe (Spain, the Czech Republic, the U.K., and Italy), Asia (China and India), and North America (Canada) were the nearest neighbours of parasitoids reared in our study from *D. sibiricus* (Figure 5). In Ichneumonoidea, the nearest neighbours were identified in BOLD and/or GenBank only for the representatives of Braconidae (Figure 5A).

The nearest neighbour of *Aleiodes esenbeckii* from Siberia, which we resurrected below as a valid subspecies, was *A. esenbeckii* from Spain (process ID GBMIN74556-17), with an interspecies divergence of 3.72% and a shared BIN (Figure 5A, Table 1). Two specimens of *G. liparidis* sequenced from Irkutsk Province (Siberia) showed the highest proximity to that from the Czech Republic (process ID GBMIN74375-17), with 0.92% intraspecific diversity, and shared the same BIN (Figure 5A, Table 1).

Another braconid, *Cotesia ordinaria*, was possible to identify in BOLD to the genus level only, with the nearest neighbour *Cotesia* sp. from the U.K. The species of *Cotesia* from Siberia and the U.K. shared one BIN and showed only 1.8% genetic divergence, suggesting that the British specimen is undoubtedly *C. ordinaria.*

Two representatives of Ichneumonidae from Siberia, *Habronyx heros* and *Hyposoter validus*, were impossible to identify in BOLD and/or GenBank even to the genus level. Thus, no nearest neighbours from the same genera are indicated in the tree (Figure 5A).

In Chalcidoidea, four species from Siberia grouped with relatives from Europe, Asia, and Canada (Figure 5B). Among them, *Trichogramma dendrolimi* from Irkutsk Province (with a maximal genetic variability of 2.05% with the population) showed the highest proximity to the species representatives from Italy and China, with 2.62% and 2.9% divergence, respectively (Table 2).

Other representatives of Chalcidoidea from Siberia showed similarity to the congeners from South and South-eastern Asia and Canada that are deposited in GenBank. In particular, the nearest neighbour of *Perilampus nitens* (Perilampidae) identified from Krasnoyarsk Territory was an unidentified representative of *Perilampus* from Indonesia (process ID BCIND760-16) (Figure 5B), with 7.4% interspecific divergence.

For *Pachyneuron solitarium* (Pteromalidae) from Irkutsk Province (Eastern Siberia), the nearest neighbour turned out to be an unidentified pteromalid from Canada (process ID GMOUF696-15) (Figure 5B), with 8.09% interspecific divergence. *Ooencyrtus pinicolus* (Encyrtidae) identified from Tuva Republic (Southern Siberia) showed 2.48% intraspecific variability. Its nearest neighbour turned out to be an unidentified species of *Ooencyrtus* from India (process ID GBMNC44807-20) (Figure 5B), with 8.74% interspecific divergence.

The superfamily Platygastroidea was represented in the genetic analysis by one species, *Telenomus tetratomus* (Scelionidae), associated with *D. sibiricus* in Siberia (Figure 5C). Reared from the pest eggs collected in Irkutsk Province (Eastern Siberia), this species showed 0.44% variability in DNA barcoding fragments and 0.65% intraspecific divergence when compared against the species representatives from Canada (process IDs CNNHB2693-14 and CNNHG1972-14).

Among the studied tachinids, the representatives from Tuva Republic (Siberia) were identified morphologically as *Masicera sphingivora*. They clustered with *Masicera silvatica* (Fallén, 1810) from France (process ID TACFI580-12), with 3.3% interspecies divergence (Figure 6, Table 3).

The nearest neighbours of the tachinid *Exorista larvarum* from Tuva Republic were two species representatives—one from Japan (process ID GBDP14050-13), with 1.03% divergence in the DNA barcoding fragment, and one from Canada (process ID SSPAA2893-13), with 1.07% divergence. They all shared one BIN in BOLD (Figure 6). The maximal divergence in *E. larvarum* reached 1.6% (Table 3). The interspecific divergence for the species pairs (*Masicera sphingivora—Exorista larvarum*) reached 12.2% (Table 3).

### 3.2. Parasitoids Identified from Northern Asia Based on Morphology

In total, 860 parasitoid specimens were included in this study, comprising 630 freshly collected specimens and 230 archival specimens. These specimens were identified as belonging to 16 parasitoid species. Of these, 14 species were documented in the contemporary *D. sibiricus* foci in Siberian regions, while 4 species were identified from earlier collections in Northern Asia, Korea, and Mongolia that are archived in ZISP (St. Petersburg) and SIF (Krasnoyarsk). Among the 16 parasitoid species, 14 belonged to Hymenoptera representing eight families: Ichneumonidae (4 species), Braconidae (4), Perilampidae (1), Pteromalidae (1), Encyrtidae (1), Trichogrammatidae (1), and Scelionidae (1). The other two species were from Diptera (family Tachinidae). The majority of parasitoid species were documented in Irkutsk Province (nine species), followed by records in Krasnoyarsk Territory (six), Tuva Republic (four), Tomsk Province (two), Novosibirsk Province (one), Buryatia Republic (one), and Sakhalin Province (one).

The taxonomic status of the braconid *Aleiodes esenbeckii* (Hartig, 1838) from Siberia was resurrected from synonymy as a valid subspecies, *Aleiodes esenbeckii* ssp. *dendrolimi*, status nov. (the diagnosis is given below in the species data). In addition to Northern Asia, it was identified among archival specimens from Korea (unknown collection year) and Mongolia (1905); its other subspecies, *A. esenbeckii* ssp. *esenbeckii*, represented a first record for the Republic of Karelia (Russia) and Belarus.

Another braconid, *G. liparidis*, studied among the specimens stored in ZISP (St. Petersburg), represented a new record for six administrative regions in Russia (Moscow, Ulyanovsk, Volgograd Provinces, the Republics of Buryatia and Yakutia, and Amur Province) and three countries: Georgia, Armenia, and Azerbaijan. The ichneumonid, *Hyposoter validus* represented a new geographical record for Tomsk Province based on our sampling in 2018–2019. The braconid *Meteorus versicolor* reared in July 2022 from *D. sibiricus* larva from Irkutsk Province was documented as a parasitoid of *D. sibiricus* for the first time.

Additionally, based on the investigated museum material, the egg parasitoid, *Telenomus tetratomus* (Thomson, 1861), was for the first time recorded as a parasitoid of two other Lasiocampidae moths, *Euthrix potatoria* (Linnaeus, 1758) and *Eriogaster lanestris* (Linnaeus, 1758).

Below, the list of identified parasitoids with data on the examined material, species distribution, and hosts is provided. Images of parasitoid adults are given.

**Order Hymenoptera Linnaeus**, **1758**

**Superfamily Ichneumonoidea Latreille**, **1802**

**Family Ichneumonidae Latreille**, **1802**

**Subfamily Campopleginae Foerster**, **1869**

***Hyposoter validus* (Pfankuch**, **1921)**

Figure 7A,B

*Material examined* (a total of three specimens). Russia: Tomsk Province, Asinovskiy District, Baturino, 57°44′01″ N 85°10′16″ E, from the larva of *D. sibiricus*, 21 September 2018 (par. em.), A.A. Ageev col., No. 15, one female (apex of metasoma damaged) DNA-barcoded (sample ID NK1498, process ID DSPAR014-22); same label, but No. 04, one adult DNA-barcoded (sample ID NK1488, process ID DSPAR004-22). Krasnoyarsk Territory, Irbeyskiy district, Stepanovka Village, bank of the Kungus River, 55°3′42″ N 96°1′19″ E, 25.VIII.2019 S.A. Astapenko col., from larva of *D. sibiricus*, one female.

*Hosts*. Probably monophagous primary solitary larval endoparasitoid of *D. sibiricus* [19,93].

*Distribution*. Russia: Krasnoyarsk Territory, *Tomsk, Irkutsk, and Sakhalin Provinces. Norway, Germany, and Poland [90,93].

*Remarks.* The specimen barcoded from Siberia represents the first DNA barcode of *H. validus* in BOLD and GenBank.

**Subfamily Pimplinae Wesmael**, **1845**

***Iseropus stercorator* (Fabricius**, **1793)**

Figure 7C,D

*Material examined* (a total of three specimens). Russia: Irkutsk Province, Kachugskiy District, Kachug, Kachugskiy Forestry, 53°55′30″ N 105°48′01″ E, from the pupa of *D. sibiricus*, 18.VI.2022 (host col.), 25. VII.2022 (par. em.), A.A. Ageev col., No. 5, one female; same label, but 04.VII.2022 (par. em.), A.A. Ageev col., No. 9, one male; same label, but 04.VII.2022 (par. em.), S.A. Astapenko col., No. 7, one female.

*Hosts*. This is a polyphagous species, a primary larval parasitoid of many families of Lepidoptera, in particular, Erebidae, Gelechiidae, Lasiocampidae, Noctuidae, Nolidae, Pyralidae, Tortricidae, Yponomeutiodae, and Zygaenidae [93]. Among the representatives of Lasiocampidae, the following moth species are listed as hosts: *Dendrolimus pini* (Linnaeus, 1758), *D. sibiricus*, *D. spectabilis* (Butler, 1877), *D. superans* (Butler, 1877), *Euthrix potatoria* (Linnaeus, 1758), *Malacosoma disstria* Hübner, 1820, and *M. neustria* (Linnaeus, 1758) [93].

*Distribution*. Russia: Kaliningrad, Leningradskaya, Yaroslavl, Penza, Tambov, Saratov, Samara, Kursk, Voronezh, Astrakhan Provinces, Perm Territory, Novosibirsk, Irkutsk Provinces, Yakutia Republic, Amur, Sakhalin, Magadan Provinces, Khabarovsk, and Primorskiy and Kamchatka Territories. Algeria, Europe (widely), Georgia, Armenia, Türkiye, Iran, Kazakhstan, Uzbekistan, Mongolia, China, and Japan; and Canada and the USA [85,93].

**Subfamily Anomaloninae Viereck**, **1918**

***Habronyx heros* (Wesmael**, **1849)**

Figure 7E,F

*Material examined* (a total of one specimen). Russia: Krasnoyarsk Territory, Irbeyskiy district, Stepanovka Village, bank of the Kungus River, 55°2′20″ N 96°1′30″ E, 25.VI.2019, S.A. Astapenko col., from the pupa of *D. sibiricus*, DNA-barcoded (sample ID NK1486, process ID DSPAR002-22), one female.

*Hosts*. Oligophagous, a primary solitary pupal endoparasitoid of Lasiocampidae (Lepidoptera): *Dendrolimus pini*, *D. punctatus* (Walker, 1855), *D. spectabilis*, *D. sibiricus*, *D. superans*, *D. superans albolineatus* (Matsumura, 1921), *Lasiocampa quercus* (Linnaeus, 1758), *Macrothylacia rubi* (Linnaeus, 1758) and *Pachypasa otus* (Druyry, 1773) [93]. Also, this parasitoid was documented from the representatives of Noctuidae and Sphingidae (Lepidoptera) [93].

*Distribution*. Russia: Leningradskaya, Vologda, Kostroma, Moscow Provinces, Zabaikalskiy Territory, Tomsk, Amur, Sakhalin Provinces, Primorskiy Territory. Europe (widely), Türkiye, Israel, China, Korea, and Japan [85,93].

***Therion circumflexum* (Linnaeus**, **1758)**

Figure 7G,H

*Material examined* (a total of one specimen). Russia: Krasnoyarsk Territory, Irbeyskiy district, Stepanovka Village, bank of the Kungus River, 55°2′20″ N 96°1′30″ E, 25.VI.2019, S.A. Astapenko col., from the pupa of *D. sibiricus*, one female.

*Hosts*. Oligophagous, a primary solitary pupal endoparasitoid of Lasiocampidae (Lepidoptera): *Dendrolimus pini*, *D. spectabilis*, *D. sibiricus*, *D. superans*, *D. superans albolineatus*, *Lasiocampa trifolii* (Denis & Schiffermüller, 1775) [93]. Also, this parasitoid was documented from the representatives of Erebidae, Geometridae, Noctuidae, Notodontidae, and Sphingidae (Lepidoptera) [93].

*Distribution*. Russia: Leningradskaya, Kirov, Yaroslavl, Vladimir, Moscow, Tula, Ryazan, Bryansk, Samara, Tambov, Rostov, Astrakhan Provinces, Perm Territory, Irkutsk, Sakhalin Provinces, and Primorskiy Territory. Algeria, Europe (widely), Georgia, Azerbaijan, Türkiye, Israel, Mongolia, China, Korea, and Japan; India; and Canada and the USA [85,93].

**Family Braconidae Nees**, **1811**

**Subfamily Rogadinae Foerster**, **1863**

***Aleiodes esenbeckii* (Hartig**, **1838) ssp. *dendrolimi* (Matsumura**, **1926)**, **status nov.**

**Figure 8:**
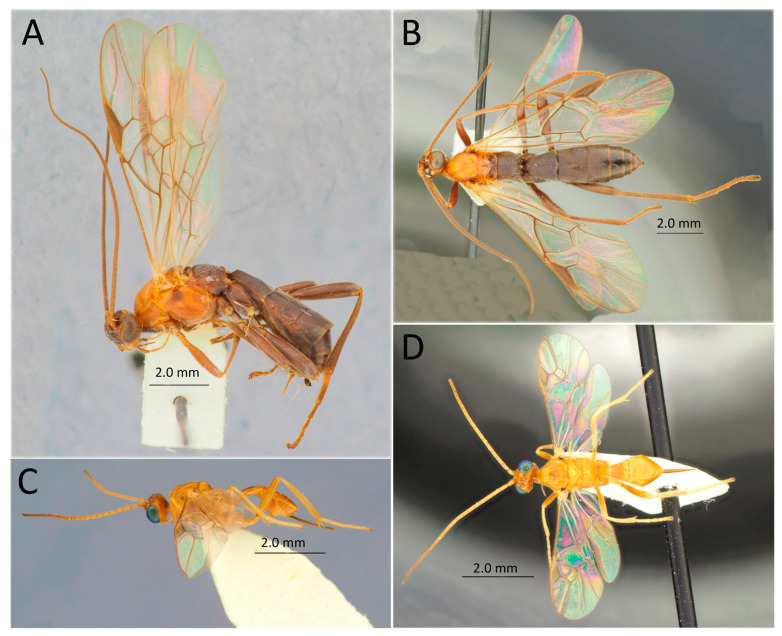
Parasitoids from the family Braconidae, (**A**,**C**) lateral view and (**B**,**D**) dorsal view. (**A**,**B**) *Aleiodes esenbeckii* (Hartig) ssp. *dendrolimi* (Matsumura) (Rogadinae), Tomsk Province and (**C**,**D**) *Meteorus versicolor* (Wesmael) (Euphorinae), Irkutsk Province. Photos: S.A. Belokobylskij.

**Figure 9:**
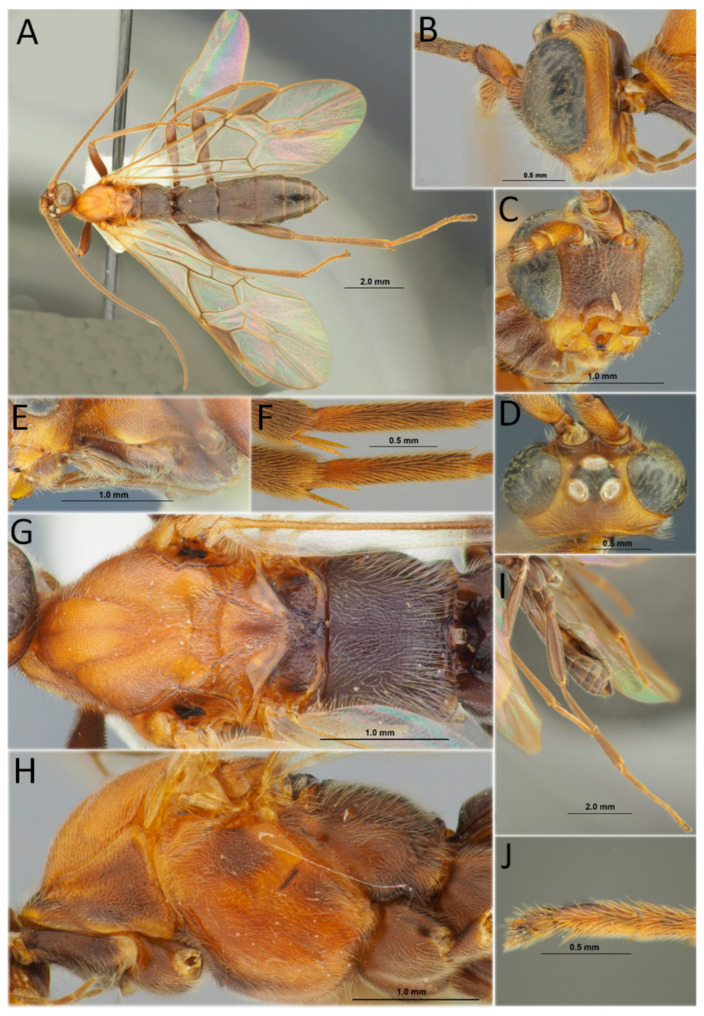
Parasitoid *Aleiodes esenbeckii* ssp. *dendrolimi* (Matsumura) (Rogadinae), Tomsk Province: (**A**) body, dorsal view; (**B**) head, lateral view; (**C**) head, front view; (**D**) head, dorsal view; (**E**) palpi; (**F**) spurs of hind tibia; (**G**) mesosoma, dorsal view; (**H**) mesosoma, lateral view; (**I**) hind leg; and (**J**) claw of hind tarsus. Photos: S.A. Belokobylskij.

**Figure 10:**
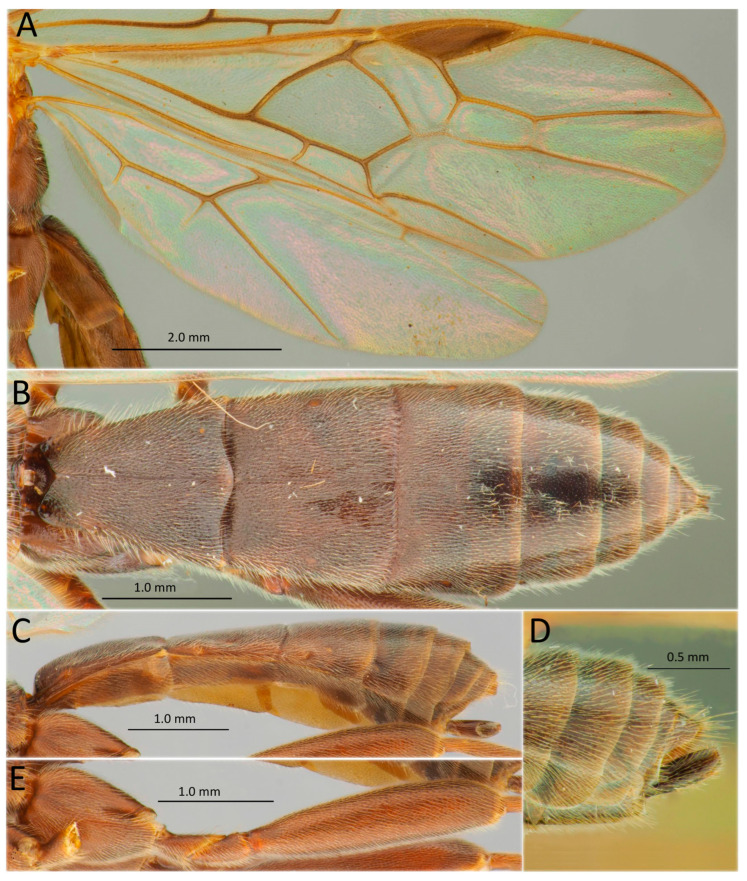
Parasitoid *Aleiodes esenbeckii* (Hartig, 1838) ssp. *dendrolimi* (Matsumura) (Rogadinae), Tomsk Province: (**A**) wings; (**B**) metasoma, dorsal view; (**C**) metasoma, lateral view; (**D**) apex of metasoma and ovipositor, lateral view; and (**E**) coxa and femur of hind leg. Photos: S.A. Belokobylskij.

Figure 8A,B, Figure 9 and Figure 10

*Material examined* (a total of 107 specimens). Russia: Sakhalin Province, North Sakhalin, Okha, emerged from the larva of *D. sibiricus*, 4.VIII.1964, D. Kasparyan col, 20 females, seven males. Irkutsk Province, Kachugskiy District, Kachug, Kachugskiy Forestry, 53°55′30″ N 105°48′01″ E, 28.VII.2020, from the larva of *D. sibiricus*, A.A. Ageev col., No. 23, two males, one male, including one specimen DNA-barcoded (sample ID NK1511, process ID DSPAR027-22); same label, but 12.VI.2022 (host col.), No. 1, one female; same label, 18.VI.2022 (host col.), 23.VI.2022 (par. em.), No. 4, one male; same label, 18.VI.2022 (host col.), 24.VI.2022 (par. em.), No. 6, one male; Kultuk, 27.VII.1927, emerged from the small larva of *D. segregatus* Butler (=*Dendrolimus sibiricus*), D. Froloff col., two females; same label, but 9.VII.1927, ex “pupa” (=mummy) of *D. segregatus* (=*D. sibiricus*), one female; bank of Baikal Lake, emerged from *D. sibiricus*, VI.1949, Bondarev col., four females. Buryatia Republic, Kyakhta District, 2.VII.1966, emerged from the larva of *D. sibiricus*, Mikhaylov col., one female. Tuva Republic, “Khaybar”, from *D. sibiricus*, VI.1958, N. Kolomiets col., two females, one male.

*Material examined (continuation).* Krasnoyarsk Territory, Irbeyskiy District, Irbeyskiy Village, Irbeyskiy Forestry, 55°01′54″ N 91°01′56″ E, from the larva of *D. sibiricus*, S.A. Astapenko col., 15.VI.2021, No. 17, one female DNA-barcoded (sample ID NK1500, process ID DSPAR016-22); same label, No. 18, one female DNA-barcoded (sample ID NK1501, process ID DSPAR017-22); same label, but 30.IV.2020, A.N. Golovina col., No. 28, three females, including one specimen DNA-barcoded (sample ID NK1516, process ID DSPAR032-22); same label, No. 20, three females, including two females DNA-barcoded (sample ID NK1517, process ID DSPAR033-22 and NK1514, DSPAR030-22); Irbeyskiy district, Stepanovka Village, bank of the Kungus River, 26.VIII.2019, S.A. Astapenko col., from the larva of *D. sibiricus*, one female DNA-barcoded (sample ID NK1487, process ID DSPAR003-22); Bolshemurtinskiy District, landmark Vangino, 23.VII.1957, emerged from *D. sibiricus*, Lipatova col., three females, one male. Novosibirsk Province, Tashara Village, 29.VI.1962, N. Kolomiets col., three females. Tomsk Province, Shegarskiy District, Mel’nikovo Village, Shegarskiy Forestry, 56°50′11″ N 83°24′21″ E, from the larva of *D. sibiricus*, S.A. Astapenko col., 19.IX.2018, No. 21, two females, two males, including two specimens DNA-barcoded (sample ID NK1508, process ID DSPAR024-22; NK1509, DSPAR025-22); same label, No. 22, two males, one female DNA-barcoded (sample ID NK1510, process ID DSPAR026-22); same label, No. 24, one female DNA-barcoded (sample ID NK1512, process ID DSPAR028-22); same label, No. 25, one male DNA-barcoded (sample ID NK1513, process ID DSPAR029-22); Asinovskiy District, Baturino, 57°44′01″ N 85°10′16″ E, from larva of *D. sibiricus*, 21.IX.2018, A.A. Ageev col., No. 16, one male DNA-barcoded (sample ID NK1499, process ID DSPAR015-22); Tomsk District, Batalino Village, VI.1955, from *D. sibiricus*, N. Kolomiets col., twenty-three females, five males; Karelia: “Kivach” Natural Reserve, light trap, 15–18.X.1990, Kutenkova leg., one female. Korea: unknown locality and data, emerged from *D. sibiricus*, (Hoen Ok Won), one female, one male. Mongolia: Urga (=Ulan-Bator), Tola (=Tuul) River, 5.VII.1905, P.K. Kozlov col., one female. Belarus: Grodno, *Dendrolimus pini* L., 20.V.1968, L. Entin leg., four females, one male.

*Hosts*. Oligophagous, primary solitary larval endoparasitoid of Lasiocampidae: *Dendrolimus pini*, *D. punctatus*, *D. spectabilis*, *D. sibiricus*, *D. superans*, *D. tabulaeformis* Tsai and Liu, 1962, and *Cosmotriche lobulina* (Denis & Schiffermüller, 1775) [93].

*Distribution*. *Aleiodes esenbeckii* ssp. *esenbeckii*: Russia: St. Petersburg, *Republic of Karelia. Norway, the Netherlands, France, Spain, Germany, Austria, Croatia, the Czech Republic, Hungary, Poland, Lithuania, *Belarus, Iran (verification required), and Afghanistan (verification required), as per the records from the collection of ZISP and the literature data [85,92,93].

*Aleiodes esenbeckii* ssp. *dendrolimi*: Russia: Altai Territory, Buryatia and Tuva Republics, Tomsk, Omsk, Novosibirsk, Kemerovo, Irkutsk, Sakhalin Provinces, Zabaikalskiy, and Krasnoyarsk and Primorskiy Territories. Switzerland, Finland (very rarely in both countries), Mongolia, China (widely), Korea, and Japan.

*Remarks.* For a long time, the Eastern Palaearctic species *Aleiodes dendrolimi* (Matsumura, 1926) was considered as a synonym of *A. esenbeckii* (Hartig, 1838) [83,93]. However, Belokobylskij [84], after studying the abundant material of the first form (*A. dendrolimi*) from the Asian part of Russia, showed that it has stable differences that allow for distinguishing it from *A. esenbeckii* s. str. The form *dendrolimi*, which is predominant in the East, has a dark colouration of the body (especially legs, palpi, and metasoma). As a result, this name was here restored as a valid species. Later, van Achterberg and M. Shaw [92], in their revision of the Western Palaearctic *Aleiodes* species, accepted only two morphological forms, *A. esenbeckii* f. *esenbeckii* and *A. esenbeckii* f. *dendrolimi*. In addition, the latter form was also very rarely recorded in the Western Palaearctic in boreal Europe (Finland and Switzerland). In the *Aleiodes* revision [92], a distinct (about 5%) barcode (COI) difference between the Mallorcan (Spanish) *A. e.* f. *esenbeckii* and Finnish *A. e.* f. *dendrolimi* was highlighted, suggesting genetic isolation of these populations. Similarly, a pronounced genetic distance (3.72%) between Siberian populations of *A. e.* f. *dendrolimi* (from Krasnoyarsk Territory and Tomsk and Irkutsk Provinces) and the European population of *A. esenbeckii* from Spain (data mined in GenBank) was also revealed (see Table 2). Such data supported our decision to separate the Asian form from the European one and consider these forms as subspecies. Below, we provide the identification key for these two subspecies.

**Key to subspecies of the species *Aleiodes esenbeckii***

1. Body usually entirely yellow or brownish yellow; rarely metasoma in posterior half infuscate, very rarely metasoma almost entirely brownish. Antenna 55–61-segmented. Western Palaearctic. Body length 7.5–11.5 mm ……………………………………………. *A. e.* ssp. *esenbeckii*

— Head partly, often palpi, always mesosoma at least ventrally and posteriorly, entirely metasoma and legs more or less brown, dark brown or even blackish; sometimes mesosoma reddish brown with dark dorsally and almost black posteriorly. Antenna 54–63-segmented. Predominantly Eastern Palaearctic. Body length 8.5–10.5 mm ………………………………………………………………………….. *A. e.* ssp. *dendrolimi*

**Subfamily Euphorinae Foerster**, **1863**

***Meteorus versicolor* (Wesmael**, **1835)**

Figure 8C,D

*Material examined* (a total of one specimen). Russia: Irkutsk Province, Kachugskiy District, Kachug, Kachugskiy Forestry, 53°55′30″ N 105°48′01″ E, 18.VI.2022, No. 10, from the larva of *D. sibiricus*, A.A. Ageev col., one female.

*Hosts*. Oligophagous, primary solitary larval endoparasitoid of Lepidoptera from the family Lasiocampidae: *Dendrolimus pini*, *D. spectabilis*, *D. sibiricus, *Macrothylacia rubi*, *Malacosoma castrense* (Linnaeus, 1758), *M. neustria* (Linnaeus, 1758), *M. parallela* Staudinger, 1887, and *Selenephera lobulina* (Denis & Schiffermüller, 1775) [93].Also, this parasitoid was documented from lepidopteran species of the families Erebidae, Geometridae, Lymantriidae, Noctuidae, Nolidae, Notodontidae, and Thaumetopoeidae [93].

*Distribution*. Russia: Leningradskaya, Novgorod, Smolensk, Bryansk, Voronezh, Saratov, Rostov Provinces, Krasnodar Territory, Bashkortostan, Tyumen, Novosibirsk, Tomsk Provinces, Altai Territory, Buryatia, Irkutsk, Amur Provinces, Kamchatka, Khabarovsk, and Primorskiy Territories. Europe (widely), Georgia, Armenia, Azerbaijan, Türkiye, Israel, Palestine, Iran, Uzbekistan, Tajikistan, Kazakhstan, Mongolia, China, Korea, and Japan; and Canada, the USA, and Mexico [85,93].

*Remarks. Meteorus versicolor* is known as a parasitoid of many macrolepidopterans, including Lasiocampidae. Before our study, this species was not known from *D. sibiricus.*

**Subfamily Microgastrinae Foerster**, **1863**

***Cotesia ordinaria* (Ratzeburg**, **1844)**

Figure 11A,B

*Material examined* (a total of 78 specimens). Russia: Krasnoyarsk Territory, Irbeyskiy district, Irbeyskiy Village, Irbeyskiy Forestry, 55°01′54″ N 91°01′56″ E, from the larva of *D. sibiricus*, 30.IV.2020, A.N. Golovina col., No. 19, 25 females, four males, including two specimens DNA-barcoded (sample ID NK1502, process ID DSPAR018-22; NK1503, DSPAR019-22); same label, No. 19(1), 18 females, three males, including two specimens DNA-barcoded (sample ID NK1504, process ID DSPAR020-22; NK1505, DSPAR021-22); Irbeyskiy district, Stepanovka Village, bank of the Kungus River, 26.VIII.2019, S.A. Astapenko col., from the larva of *D. sibiricus*, 1 female. Irkutsk Province, Kachugskiy District, Kachug, Kachugskiy Forestry, 53°55′30″ N 105°48′01″ E, from the larva of *D. sibiricus*, 16.VII.2021, A.A. Ageev col., No. 20, 17 females, 10 males, two ex., including two specimens DNA-barcoded (sample ID NK1506, process ID DSPAR022-22; NK1507, DSPAR023-22).

*Hosts*. Oligophagous, a primary gregarious larval endoparasitoid of Lepidoptera, Lasiocampidae: *D. pini*, *D. punctatus*, *D. spectabilis*, *D. sibiricus*, *D. superans*, *D. superans albolineatus*, *D. tabulaeformis*, and *Macrothylacia rubi* [93]. Also, this parasitoid was reared from the moth Amata palestinae Hampson, 1898 (Erebidae) [93].

*Distribution*. Russia: Yaroslavl, Tomsk, Krasnoyarsk Provinces, Buryatia, Tuva, Irkutsk, Amur, Sakhalin Provinces, and Primorskiy Territory. Europe (the U.K., Germany, Italy, the Czech Republic, Hungary, Poland, Romania, and Ukraine), Türkiye, Israel, Iran, Mongolia, China (widely), Korea, and Japan [85,93].

*Remarks.* In BOLD, the Siberian specimen showed 98.19% similarity to an unidentified species of *Cotesia* (sample ID MRS_JFT0716) from the U.K. The DNA barcode of the specimen from Siberia is the first for *C. ordinaria* in BOLD.

***Glyptapanteles liparidis* (Bouché**, **1834)**

Figure 11C,D

*Material examined* (a total of 61 specimens). Russia: Irkutsk Province, Kachugskiy District, Kachug, Kachugskiy Forestry, 53°55′30″ N 105°48′01″ E, from larva of *D. sibiricus*, 18.VII.2021, A.A. Ageev col., No. 12, two males DNA-barcoded (sample ID NK1489, process ID DSPAR005-22; NK1490, DSPAR006-22); Moscow Province: Mytishchi, from *Lymantria dispar*, 17.VI.1963, Semevskiy leg., five females, one male; Chashnikovo, from *L. dispar*, VII–VIII.1958, 5.VIII.1959, without collector, four females, three males; Ulyanovsk Province: Ulyanovsk, 1987, Zolotarev leg., two females; Volgograd Province: Kikvidze, forest belt, 1997. Yu. Mukhin leg., one female, three males; Buryatia: Khilok River, from *D. sibiricus*, 1950 and 22.VII.1962, Boldaruev leg. ten females, one male; Kyakhta, 16.VII.1967, without collector, 1 female; Mukhorshibir’, 16.VII.1967, without collector, one female, one male; Yakutia: Yakutia, Pokrovsk, 29.VI.2001, without collector, one female, one male; Amur Province: Shimanovsk, from *D. sibiricus*, 17.VIII.1966, D. Kasparyan leg., three females, three males. Georgia: Khashuri, from *Dendrolimus pini*, 18.V.1979, Zharkov leg., two males; same label, but, 12.VII.1979, two females; same label, but, 22.VIII.1979, two females. Armenia: three females, two males, Dilizhan, forest, 8.VI.1971, Kuslitsky leg.; three females, Tsav, forest, 26.VII.1971, Kuslitsky leg.; two females, one male, Tsav, 1800 m, forest, 4.VII.1971, Kuslitsky leg. Azerbaijan: one female, Pirkuli, reared from a caterpillar of Geometridae, 18.VII.1988, Piriev leg.

*Hosts*. Polyphagous, a primary solitary or gregarious larval endoparasitoid of Lepidoptera from the family Lasiocampidae: *D. pini*, *D. punctatus*, *D. spectabilis*, *D. sibiricus*, *D. superans*, *D. superans albolineatus*, *D. tabulaeformis*, and *Eriogaster lanestris* (Linnaeus, 1758) [90]. Also, this parasitoid was documented from representatives of the families Erebidae, Notodontidae and Noctuidae (Lepidoptera) [93].

*Distribution*. Russia: Kaliningrad, Leningradskaya, Yaroslavl, *Moscow, *Ulyanovsk, Saratov, Voronezh, *Volgograd Provinces, Krasnodar Territory, Tomsk, Novosibirsk, Irkutsk Provinces, *Buryatia, *Yakutia, Chita, *Amur, Sakhalin Provinces, Khabarovsk, and Primorskiy Territories. Europe (widely), *Georgia, *Armenia, *Azerbaijan, Kazakhstan, Iran, Mongolia, China (widely), Korea, and Japan; India; and the USA (introduced), as per records from the collection of ZISP and the literature data [85,93].

*Remarks.* The determination of the microgastrins based on males is problematic. According to the morphological characters, we assigned two specimens (both males) from Siberia to *G. liparidis*. DNA barcoding of these specimens showed high proximity to *G. liparidis* from the Czech Republic (process ID GBMIN74375-17), with only 0.92% divergence.

**Superfamily Chalcidoidea Latreille**, **1817**

**Family Perilampidae Latreille**, **1809**

***Perilampus nitens* Walker**, **1834**

Figure 12A,B

*Material examined* (a total of one specimen). Russia: Krasnoyarsk Territory, Irbeyskiy district, Irbeyskiy Village, Irbeyskiy Forestry, 55°01′54″ N 91°01′56″ E, 15.VII.2020, S.A. Astapenko col., No 27, from a mummy of the larva of *D. sibiricus* infested by *A. esenbeckii* ssp. *dendrolimi*, one female DNA-barcoded (sample ID NK1515, process ID DSPAR031-22).

*Hosts*. Associated with Lasiocampidae (Lepidoptera). Primary parasitoid of *D. sibiricus* (Lepidoptera: Lasiocampidae), secondary parasitoid of *Aleiodes esenbeckii* ssp. *dendrolimi* (Matsumura, 1926) (Hymenoptera: Braconidae), and some species of the family Tachinidae (Diptera) [69,70,97].

*Distribution*. Russia: Republic of Karelia, Vologda, Leningradskaya, Kirov Provinces, Perm and Zabaikalskiy Territories, Tomsk, Amur Provinces, and Khabarovsk and Primorskiy Territories. Europe (widely) [85,94].

**Family Pteromalidae Dalman**, **1820**

**Subfamily Pachyneurinae Ashmead**, **1904**

***Pachyneuron solitarium* (Hartig**, **1838)**

Figure 12C–F

*Material examined* (a total of 13 specimens). Russia: Irkutsk Province, Kachugskiy District, Kachug, Kachugskiy Forestry, 53°55′30″ N 105°48′01″ E, No. 35, from the pupa of *D. sibiricus*, 28.VII.2020, A.A. Ageev col., one female, three males, including two specimens DNA-barcoded (sample ID NK1491, process ID DSPAR007-22 and NK1494, DSPAR010-22); same label, 28.VII.2020, S. Astapenko, five females, four males, including one specimen DNA-barcoded (sample ID NK1524, process ID DSPAR040-22).

*Hosts*. Polyphagous, primary on Coleoptera (Coccinellidae), Diptera (Asilidae), Hemiptera (Aphididae, Coccidae, Pseudococcidae and Psyllidae), and Lepidoptera (Lasiocampidae: *Cosmotriche lobulina* (Denis & Schiffermüller, 1775), *Dendrolimus kikuchii* Matsumura 1927, *D. pini*, *D. sibiricus*, *D. spectabilis*, *D. superans*), and Lymantriidae. Secondary parasitoid of Hymenoptera (Aphelinidae, Braconidae, Encyrtidae, and Scelionidae) [85,94].

*Distribution*. Russia: Republic of Buryatia, Primorskiy Territories, and Sakhalin Province. Europe (widely), Georgia, Kazakhstan, China, Korea, Japan, and India [85,94,95].

**Family Encyrtidae Walker**, **1837**

***Ooencyrtus pinicolus* (Matsumura**, **1926)**

Figure 13A,B

*Material examined* (a total of six specimens). Russia: Tuva Republic, Targalovka, 51°17′27″ N 92°47′31″ E, from eggs of *D. sibiricus*, 9.IX.1963 (par. em.), Yu.P. Kondakov col., four adult specimens DNA-barcoded (sample ID NK1563, process ID DSPAR079-22; NK1568, DSPAR084-22; NK1570, DSPAR086-22; NK1573, DSPAR089-22). Irkutsk Province, Kachugskiy District, Kachug, Kachugskiy Forestry, 53°55′30″ N 105°48′01″ E, from the egg of *D. sibiricus*, 26.VII.2022 (eggs coll.), 11.VIII.2022 (par. em.), A.A. Ageev col., No. 32, two females DNA-barcoded (sample ID NK1492, process ID DSPAR008-22; NK1493, DSPAR009-22).

*Hosts*. Primary egg parasitoid of Lepidoptera: Bombycidae, Erebidae, and Lasiocampidae (*Cosmotriche lobulina* (Denis & Schiffermüller), *Euthrix potatoria* (Linnaeus, 1758), *D. superans*, *D. pini*, and *D. sibiricus*) [37,87].

*Distribution*. Russia: Omsk, Tomsk, Novosibirsk, Kemerovo Provinces, Altai Territory, Khakassia, Tuva and Buryatia Republics, Krasnoyarsk and Zabaikalskiy Territories, Irkutsk, Amur and Sakhalin Provinces, and Primorskiy Territory. Kazakhstan, China, and Japan [37,85,94].

*Remarks.* The species was identified using the key of the Palaearctic Encyrtidae [34] and was verified by comparison with reference collection material identified by V.A. Trjapitzin and stored in ZISP.

**Family Trichogrammatidae Haliday** & **Walker**, **1851**

***Trichogramma dendrolimi* Matsumura**, **1926**

Figure 13C

*Material examined* (a total of 138 specimens). Russia: Irkutsk Province, Kachugskiy District, Kachug, Kachugskiy Forestry, 53°55′30″ N 105°48′01″ E, from the egg of *D. sibiricus*, 26.VII.2022 (eggs col.), 10.VIII.2022 (par. em.), A.A. Ageev col., No. 26, 27 specimens; same label, but 26.VII.2022 (host col.), 12.VIII.2022 (par. em.), No. 28, one female; same label, but 26.VII.2022 (eggs col.), 15.VIII.2022 (par. em.); same label, No. 31, 5 specimens, including 1 specimen DNA-barcoded (sample ID NK1519, process ID DSPAR035-22); same label, No. 32, 60 specimens, including 1 specimen DNA-barcoded (sample ID NK1520, process ID DSPAR036-22); same label, No. 33, 45 specimens, including 2 specimens DNA-barcoded (sample ID NK1521, process ID DSPAR037-22; NK1522, DSPAR038-22).

*Hosts*. Polyphagous, a primary egg parasitoid of Lepidoptera (Erebidae, Geometridae, Hesperiidae, Hyblaeidae, Lasiocampidae, Limacodidae, Lymantriidae, Noctuidae, Notodontidae, Nymphalidae, Pyralidae, Saturniidae, Tortricidae, and Gelechiidae). The hosts among Lasiocampidae: *D. pini*, *D. punctatus*, *D. sibiricus*, *D. spectabilis*, *Lebeda nobilis* Walker, 1855, *Adela nobilis* Christoph, 1882, and *Malacosoma neustria* [69,72,78]. Also, this species is known as a hyperparasitoid of *Pachyneuron solitarium* (Hartig, 1838) and *Euneura lachni* (Ashmead, 1887) (Hymenoptera: Pteromalidae) [68,79].

*Distribution*. Russia: Moscow Province, Krasnodar and Altai Territories, Omsk, Tomsk, Novosibirsk, Kemerovo, Irkutsk Provinces, Khakassia and Tuva Republics, Krasnoyarsk, Zabaikalskiy, Primorskiy Territories, and Sakhalin Province. Europe (widely), Türkiye, Iran, Pakistan, Kazakhstan, China, Korea, Japan, India, Vietnam, and Chile [85,94].

*Remarks.* The specimens sequenced from Siberia showed high similarity with the DNA barcodes of *Trichogramma dendrolimi* from Italy and China.

**Superfamily Platygastroidea Haliday**, **1833**

**Family Scelionidae Haliday**, **1839**

**Subfamily Telenominae Thomson**, **1861**

***Telenomus tetratomus* (Thomson**, **1861)**

*Synonyms. Telenomus bombycis* Mayr, 1879, *T. gracilis* Mayr, 1879, *T. verticillatus* Kieffer, 1917 (synonymized by Kozlov [66]).

**Figure 13:**
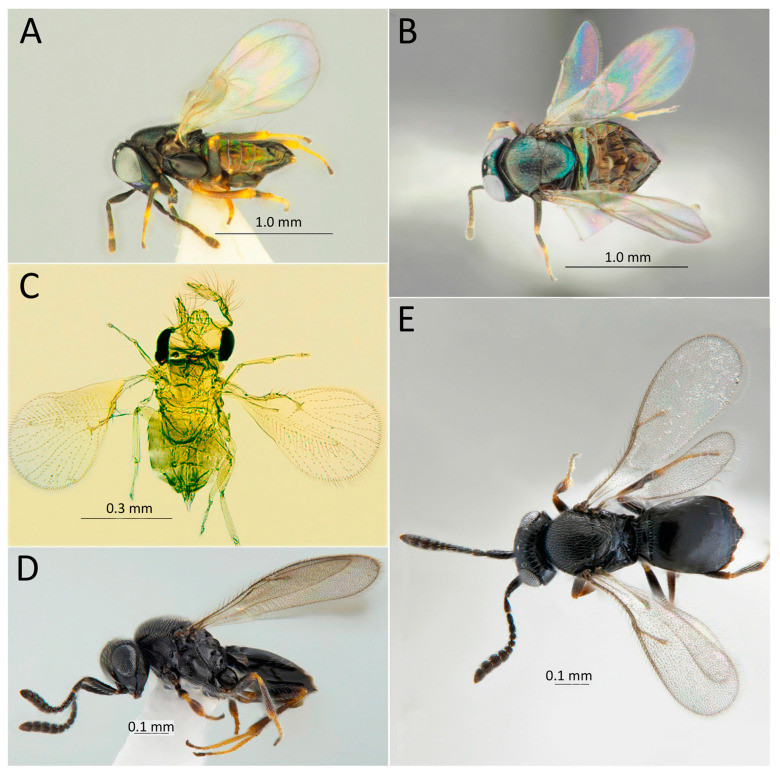
Parasitoids from the superfamily Chalcidoidea and Platygastroidea: (**A**,**D**) lateral view and (**B**,**C**,**E**) dorsal view. (**A**,**B**) *Ooencyrtus pinicolus* (Matsumura) (Encyrtidae), Irkutsk Province; (**C**) *Trichogramma dendrolimi* Matsumura (Trichogrammatidae); and (**D**,**E**) *Telenomus tetratomus* (Thomson) (Scelionidae), Irkutsk Province. Photos: S.A. Belokobylskij (**A**,**B**), E.V. Tselikh (**C**), and A.V. Timokhov (**D**,**E**).

**Figure 14:**
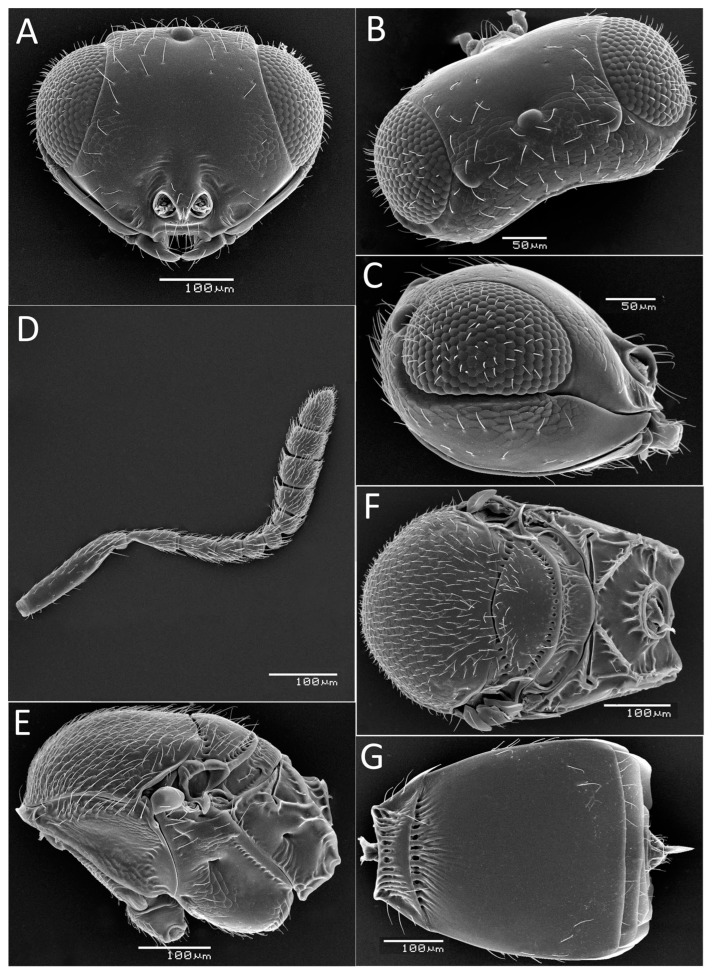
Parasitoid *Telenomus tetratomus* (Thomson, 1861) (Scelionidae), Irkutsk Province. (**A**) head, front view; (**B**) head, dorsal view; (**C**) head, lateral view; (**D**) antenna; (**E**) mesosoma, lateral view; (**F**) mesosoma, dorsal view; and (**G**) metasoma, dorsal view. Photos: A.V. Timokhov.

Figure 13D,E and Figure 14

*Material examined* (a total of 401 specimens). Russia: Irkutsk Province, Kachugskiy District, Kachug, Kachugskiy Forestry, 53°55′30″ N 105°48′01″ E, from the eggs of *D. sibiricus*, 28.VII.2022 (eggs col.), 10–15.VIII.2022 (par. em.), A.A. Ageev col., No. 14, 10 females, 12 males, including two specimens DNA-barcoded (sample ID NK1496, process ID DSPAR012-22; NK1497, DSPAR013-22); same label, No. 30, 4 females, 1 male, including one specimen DNA-barcoded (sample ID NK1518, process ID DSPAR034-22); same label, No. 34, 28 females, 3 males, including one specimen DNA-barcoded (sample ID NK1523, process ID DSPAR039-22); same label, No. 16 n/a, 18.VI.2022 (eggs col.), 10.VII.2022 (par. em.), S.A. Astapenko col., 41 females, 21 males; same label, No. 18 n/a, 48 females, 14 males; same label, No. 25, 26.VII.2022 (eggs col.), 11.VIII.2022 (par. em.), A.A. Ageev col., 21 females, 3 males; same label, No. 27; 4 females, same label, but No. 29, 105 females, 28 males; same label, No. 30, 16.VIII.2022 (par. em.), 9 females; Voronezh Province, Voronezh Nature Reserve, form the eggs of *Cosmotriche potatoria* (=*Euthrix potatoria*), 3.VII.1952, B. Smirnov leg., 5 females, 3 males. Kazakhstan, West Kazakhstan Province, Yanvartsevo, from the eggs of Eriogaster lanestris, 28.V.1950, K. Grunin leg., 22 females, 19 males.

*Hosts.* Oligophagous egg parasitoid of moths from the family Lasiocampidae: *D. pini*, *D. sibiricus*, *Macrothylacia rubi* (Linnaeus, 1758), and *Lasiocampa trifolii*, as well as *Euthrix potatoria (Linnaeus, 1758) and *Eriogaster lanestris, as per records from the collection of ZISP. The species *Calliteara abietis* (Denis & Schiffermüller, 1775) (=*Dasychira albodentata* Bremer, 1864) and *Orgyia antiqua* (Linnaeus, 1758) (Erebidae) were also reported as possible hosts [19,46,58,66]. Reports of its parasitism on *Deporaus betulae* (Linnaeus, 1758) (Coleoptera: Rhynchitidae) [19] should be considered incorrect.

*Distribution*. Russia: Voronezh, Omsk, Novosibirsk, Tomsk, Kemerovo, Irkutsk, Provinces, Altai, Krasnoyarsk and Zabaikalskiy Territories, Tuva, Buryatia and Yakutia Republics, and Amur and Sakhalin Provinces. Europe (France, Denmark, Sweden, Germany, Austria, Poland, and Belarus), Kazakhstan, China, Mongolia, and Japan [19,42,66,71,85,96].

*Remarks. Telenomus* Haliday, 1833, is a species-rich genus whose representatives are often not easy to identify, especially the species from the *Telenomus californicus* complex parasitizing the eggs of Lepidoptera [75]. The studied specimens of *Telenomus* from Siberia belong to this complex. *Telenomus tetratomus* can be identified by the combination of facial striae present as very short grooves orienting from malar sulcus to antennal foramen; temples distinctly bulging; metascutellum rectangular (about as long medially as laterally), shortly reticulate dorsally and smooth or finely striate ventrally; and second metasomal tergite almost as long as wide, distinctly tapering to its base (the ratio of the maximum width to the width at the base is 1.3:1.0).

This species is reported to be phoretic on the adults of its hosts [19,58,59], which is a rare behavioural trait for *Telenomus* [75]. Another specific feature of the biology of *T. tetratomus* is its gregarious parasitism in host eggs, while the majority of scelionids are solitary parasitoids. *T. tetratomus* females oviposit from three to twelve eggs into an egg of *D. sibiricus.* On average, about seven parasitoids develop per host egg; however, up to 25 wasps can emerge from a single host egg if superparasitism occurs [58,59].

The specimen barcoded from Siberia represents the first DNA barcode of *Telenomus tetratomus* in BOLD. In GenBank, there are two specimens (sample IDs: BIOUG17132-E12, BIOUG17632-B12) from Canada that show 100% identity with the specimens from Siberia, but they are not identified to the species; based on our identification from Siberia, these Canadian specimens may represent the same species, *Telenomus tetratomus.*

**Order Diptera Linnaeus**, **1758**

**Superfamily Oestroidea Leach**, **1815**

**Family Tachinidae Bigot**, **1853**

**Subfamily Exoristinae Robineau-Desvoidy**, **1863**

***Exorista larvarum* (Linnaeus**, **1758)**

*Synonyms. Tachina flavescens* Meigen, 1824, *T. insuscepta* Walker, 1853, *T. noctuarum* Rondani, 1865, *T. praepotens* Meigen, 1824

Figure 15A,B

*Material examined* (a total of one specimen). Russia: Tuva Republic, Ishtii-Khem, from *D. sibiricus*, 1.VIII.1963, Yu. Kondakov col., one specimen DNA-barcoded (sample ID NK1526, process ID DSPAR042-22).

*Hosts.* Pupal parasitoid of many lepidopterans, including *D. sibiricus* (Lasiocampidae) and *Lymantria dispar* (Erebidae), as well as the larvae of sawflies (Hymenoptera).

*Distribution*. Russia: Leningradskaya, Moscow, Samara Provinces, Crimea Republic, Zabaikalskiy Territory, and Amur and Sakhalin Provinces. North Africa, Europe (widely), North Africa, Middle East, Caucasus, Central Asia, Mongolia, China, and Japan; India; and Canada (introduced), as per the records from ZISP and the literature data [26,67].

***Masicera sphingivora*** **(Robineau-Desvoidy, 1830)**

*Synonyms. Musca crassiseta* Ratzeburg, 1844, *Masicera cuculliae* Robineau-Desvoidy, 1863, *M. puparum* Robineau–Desvoidy, 1863, *M. zimini* Kolomiets, 1952.

Figure 15C,D

*Material examined* (a total of 45 specimens). Russia: Sakhalin Province, South of Okha, Shkhunnyi Spring, from *D. sibiricus*, 4.VIII.1964, D. Kasparyan col., 28 specimens. Tuva Republic, Turan, from *D. sibiricus*, 15–18.VII.1963, Yu.P. Kondakov col., 2 specimens; Ishtii-Khem, from *D. sibiricus*, 20.III.1964, Yu.P. Kondakov, 1 specimen DNA-barcoded (sample ID NK1529, process ID DSPAR045-22); same locality, but 15.IV.1964, 1 specimen barcoded (sample ID NK1530, process ID DSPAR046-22); same locality, but 18.III.1964, 1 specimen DNA-barcoded (sample ID NK1531, process ID DSPAR047-22); same locality, but 18.III.1964, 1 specimen DNA-barcoded (sample ID NK1527, process ID DSPAR043-22); same locality, but 13.III.1964, 1 specimen DNA-barcoded (sample ID NK1528, process ID DSPAR044-22); same locality, but 9.III.1964, 2 specimens; same locality, but 20.IV.1964, 2 specimens; Irkutsk Province, Kachugskiy District, Kachug, 53°55′30″ N 105°48′01″ E, 23.VI–1.VII.2022 A.A. Ageev col., 10 specimens.

*Hosts.* Pupal parasitoid of *D. sibiricus* and several other species of Lasiocampidae; as well as species from the families Geometridae, Erebidae (*Euproctis chrysorrhoea* Linnaeus, 1758, *Lymantria dispar*), Noctuidae, and Sphingidae and some families of butterflies.

*Distribution*. Russia: Leningradskaya Province, Dagestan and Tuva Republics, Krasnoyarsk and Zabaikalskiy Territories, Tuva and Yakutia Republics, and Amur and Sakhalin Provinces. Europe (widely), Georgia, Armenia, Azerbaijan, Iran, Kazakhstan, Mongolia, and Japan, as per the records from the collection of ZISP and the literature data [67].

*Remarks.* The specimens barcoded from Siberia represent the first DNA barcodes of *Masicera sphingivora* in BOLD and GenBank.

### 3.3. The Checklist of Dendrolimus sibiricus Parasitoids

**Figure 16:**
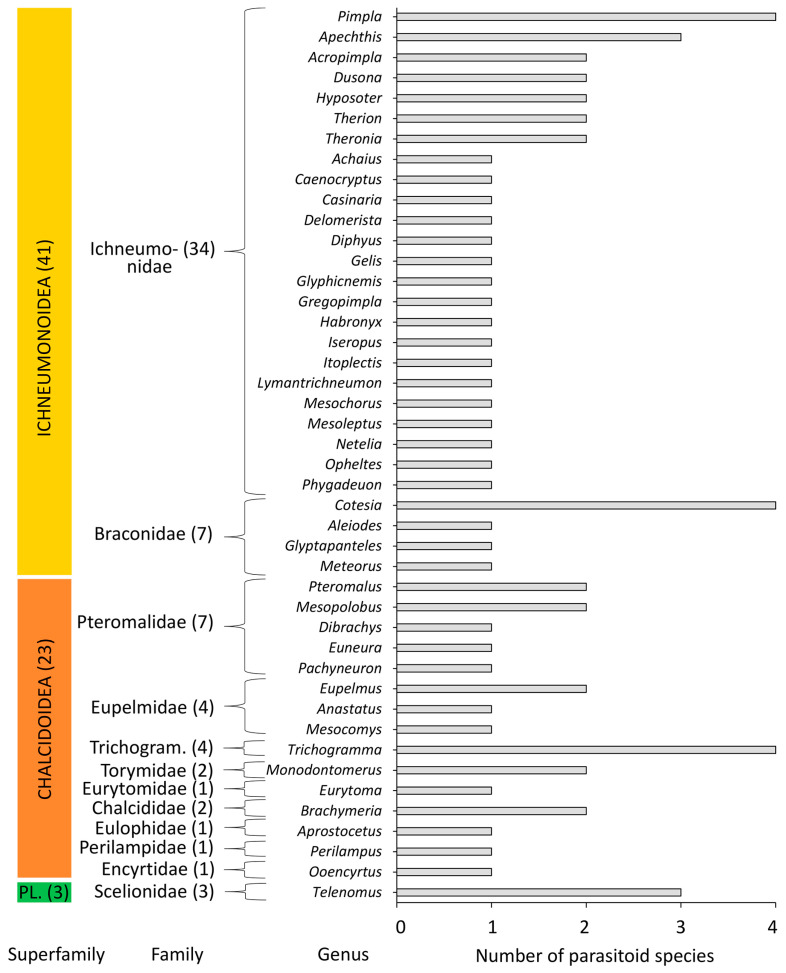
Hymenopteran parasitoids associated with *Dendrolimus sibiricus* in Northern Asia based on the literature data and our records. The number of parasitoid species is provided in brackets next to each superfamily and family; PL.—Platygastroidea, Trichogram.—Trichogrammatidae.

**Figure 17:**
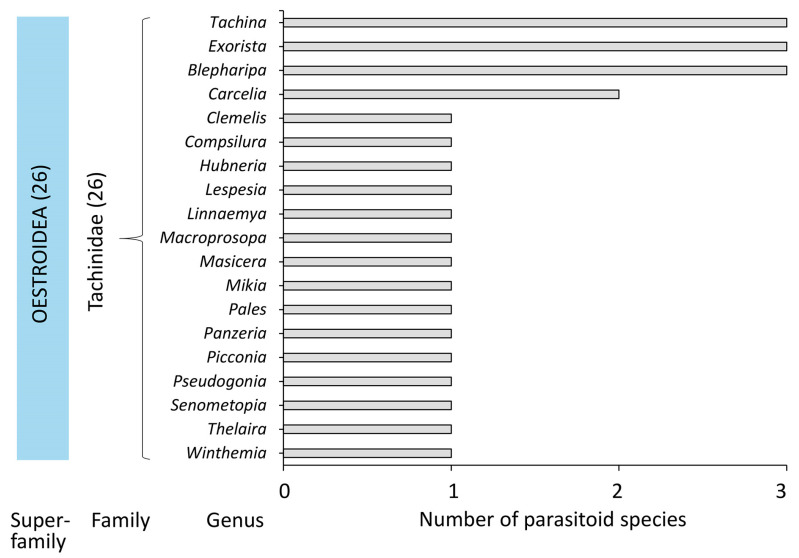
Dipteran parasitoids trophically associated with the Siberian moth, *Dendrolimus sibiricus*, in Northern Asia based on the literature data and our records. The number of parasitoid species is given in brackets next to the superfamily and family name.

Based on an exhaustive literature data survey and our observations, a list of 93 parasitoids associated with *D. sibiricus* was compiled (Table S3). In the list, 67 species (72%) are from Hymenoptera, and 26 species (28%) are from Diptera (Figure 16 and Figure 17, Table S3).

Among Hymenoptera, the representatives of three superfamilies (Ichneumonoidea, Chalcidoidea, and Platygastroidea), 12 families (Braconidae, Chalcididae, Encyrtidae, Eulophidae, Eupelmidae, Eurytomidae, Ichneumonidae, Perilampidae, Pteromalidae, Scelionidae, Torymidae, and Trichogrammatidae), and 44 genera were documented (Figure 16). The majority of parasitoids were from Ichneumonoidea at 41 species (61% of all hymenopterans associated with *D. sibiricus*), followed by Chalcidoidea (23 species, 34%) and Platygastroidea (3 species, 5%) (Figure 16). The representatives of Ichneumonidae were the richest at 34 species (i.e., 51% of all hymenopteras associated with *D. sibiricus*), followed by Braconidae (7 species, 10%) and Pteromalidae (7 species, 10%). The other 19 hymenopteran species (i.e., 29%) were the representatives of nine families (Eupelmidae and Trichogrammatidae by 4 species each; Torymidae by 2 species; Scelionidae Chalcidida, and Eulophidae by 2 species each; and Encyrtidae, Eurytomidae and Perilampidae by 1 species each) (Figure 16).

In Diptera, solely Tachinidae representatives parasitize on *D. sibiricus*. Among them, nine species (i.e., 35% of all parasitoid flies from the *D. sibiricus* checklist) are the representatives of three genera: *Tachina*, *Exorista*, and *Blepharipa* (Figure 17).

The majority of parasitoids from the *D. sibiricus* checklist (i.e., 63 out of 93 species, i.e., 68%) are generalists associated with other Lepidoptera. The remaining 27 species, in addition to Lepidoptera, have hosts among Hymenoptera, Coleoptera, and Diptera (Table S3). *Cotesia prozorovi* (Telenga, 1955), *Hyposoter validus*, and *Itoplectis tabatai* (Uchida, 1930) are the specialists known only from *D. sibiricus* and/or closely related *D. superans* [61,85,86].

Among the 93 parasitoids, 14 species (i.e., 15% of all parasitoids) develop in the eggs of *D. sibiricus*, 18 species (20%) are associated with larvae, 44 species (47%) with larvae and pupae, and 17 species (18%) with pupae. Egg parasitoids are the representatives of five hymenopteran families: Trichogrammatidae and Pteromalidae (by four species of each), Scelionidae (three) and Eupelmidae (two), and Encyrtidae (one species). Larval and larva-pupal parasitoids are the most diverse, with 62 species from the families: Tachinidae (26 species), Ichneumonidae (18), Braconidae (7), Torymidae and Pteromalidae (by 3 species each), Eupelmidae and Chalcididae (by 2 species each), and Perilampidae (1). The remaining 17 species, i.e., Ichneumonidae (16 species) and Eulophidae (1), are linked with pupae. Among larval and pupal parasitoids, 62 species are endoparasitoids, and 17 species are ectoparasitoids (Table S3).

Overall, 23 out of the 93 parasitoid species (i.e., 25%) have been reported in the literature also as hyperparasitoids, developing on some parasitoids of *D. sibiricus* (Table S3). Among them, there are 21 hymenopteran and two dipteran parasitoids (Table S3). Some parasitoids that attack *D. sibiricus* can also behave as hyperparasitoids, developing on primary parasitoids of the pest. For example, *Perilampus nitens*, a parasitoid of *D. sibiricus*, can also act as a hyperparasitoid of *Aleiodes esenbeckii* ssp. *dendrolimi*.

In addition to the 93 parasitoid species, there are 11 predatory flies (with some exhibiting parasitoid behaviour), which are known to develop on *D. sibiricus* based on the surveyed literature (Table S4). These species are treated in our study separately; they are the representatives of Sarcophagidae (nine species) and Muscidae (two species). Among them, the species from the genus *Sarcophaga* are the most diverse (seven species), followed by *Agria* (two) and *Muscina* (two). They are mostly generalists and are known to have trophic relations with a number of other lepidopteran species (Table S4).

### 3.4. Parasitism in Dendrolimus sibiricus Populations

Overall, 19 species of parasitoids, i.e., 14 hymenopterans and five dipterans, have been recorded by different authors as the most abundant in the *D. sibiricus* populations in Northern Asia and China (Table 4).

Of the 16 identified parasitoid species, 6 species target the eggs of *D. sibiricus*, while the other 13 develop in larvae and/or pupae (Table 4). Only for some species, parasitism in *D. sibiricus* populations was documented (Table S2). For instance, the egg parasitoid, *Telenomus dendrolimi*, induced high egg mortality (ranging from 56% to 94%) in *D. sibiricus* in the Russian Far East (Sakhalin and Kuril Islands) and China [47,61,76]. Another egg parasitoid, *Telenomus tetratomus*, caused up to 80% mortality in Krasnoyarsk Territory in the 1950s [19,57]. The braconid parasitoid, *Cotesia rubripes*, was documented to cause from 25% to 70% of larval mortality in *D. sibiricus* in Tomsk Region [19,57]. Furthermore, the tachinid parasitoid, *Blepharipa pratensis*, was observed to kill between 20% and 50% of the *D. sibiricus* pupae in Southern Siberia [19,91].

**Figure 18:**
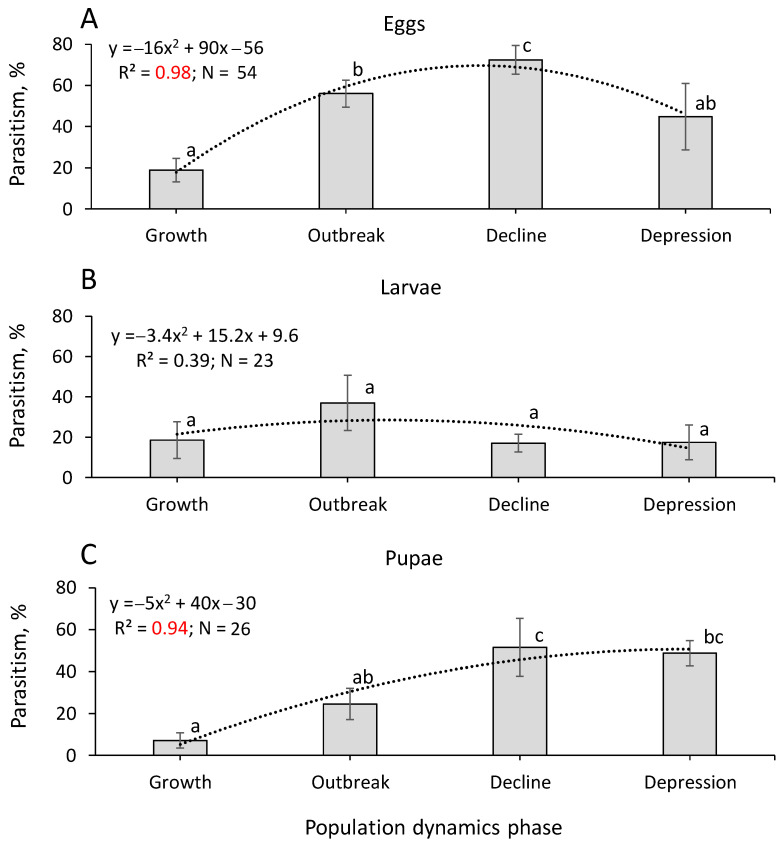
Relative parasitism (%) of eggs (**A**), larvae (**B**), and pupae (**C**) in *Dendrolimus sibiricus* populations during the growth, outbreak, decline, and depression phases in Northern Asia in the last 83 years (1940–2022). R^2^ values indicated in red are statistically significant at *p* < 0.001. The bars with different letters are significantly different (*p* < 0.05) and the bars with the same letter are not (*p* > 0.05) according to the Mann–Whitney U-test. The Z-value statistics are provided in Table S5.

Over the last 83 years (from 1940 to 2022) in Northern Asia, a total of 103 cases of parasitism have been documented, contributing to the mortality of *D. sibiricus* eggs (54 cases), larvae (23 cases), and pupae (26 cases). Among these cases, 15 were observed in the recent pest foci in Siberian regions in 2018–2022 (Table S2). Based on this dataset, we calculated average parasitism for different developmental stages of *D. sibiricus* across four population phases: growth, outbreak, decline, and depression (Figure 18).

Egg and pupal parasitism was lower in *D. sibiricus* during the population growth phase, increasing throughout the outbreak phase to the decline phase, with a subsequent decrease in the depression phase (Figure 18). In these cases, the parasitism was well described by a polynomial quadratic function with high significance (*p* < 0.001) (Figure 18). Averaged larval parasitism was not statistically different in the studied population phases (Figure 18).

**Figure 19:**
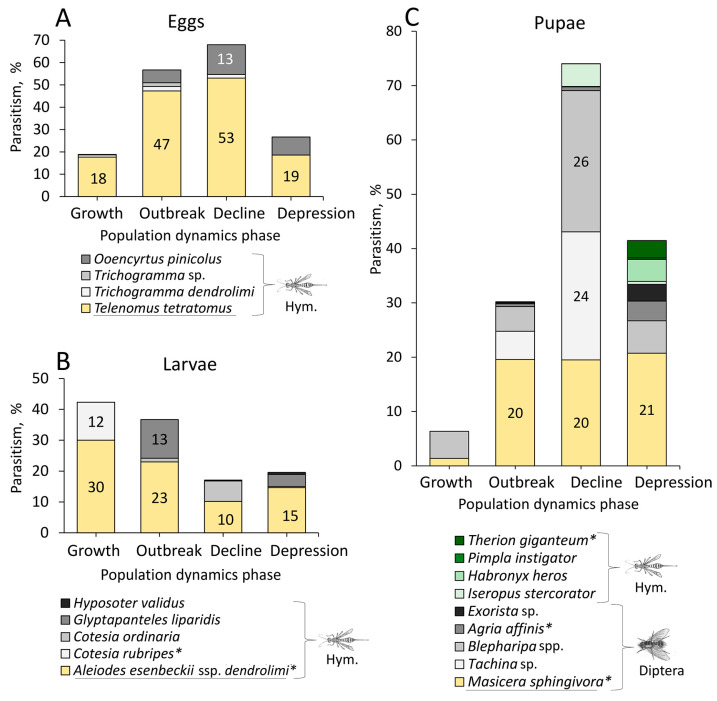
Contribution of different parasitoid species to the mortality of eggs (**A**), larvae (**B**), and pupae (**C**) in *Dendrolimus sibiricus* populations undergoing different phases as per observations in Northern Asia over 83 years (1940–2022). For the parasitoid species causing mortality ≥10%, the values of averaged parasitism (%) are indicated in the bars. The most impactful parasitoid species are underlined in the legends. For parasitoids indicated with *, the actual names are provided (whereas in the early literature, they were listed under early names, i.e., *Cotesia rubripes* was listed as *Apanteles rubripes* (Haliday, 1834), *Aleiodes esenbeckii* ssp. *dendrolimi* as *Aleiodes esenbeckii* (Harting, 1838), *Therion giganteum* as *Exochilum giganteum*, *Agria affinis* as *Pseudosarcophaga affinis* (Fallen, 1816), and *Masicera sphingivora* as *M. zimini* Kolomiets, 1952). Hym.—Hymenoptera.

The analysis of an 83-year dataset enabled us to assess the impact of various parasitoid species on the mortality of *D. sibiricus* across Northern Asia (Figure 19).

Among the parasitoids commonly detected in *D. sibiricus* foci during the long-term observation period, only a few species had a significant impact on pest mortality (Figure 19). The scelionid *Telenomus tetratomus* showed the greatest impact, killing >50% of the pest eggs (Figure 19A). It was followed by another egg parasitoid, the encyrtid *Ooencyrtus pinicolus*, which caused mortality of around 13% of *D. sibiricus* eggs (Figure 19A). Among larval parasitoids, the braconid *Aleiodes esenbeckii* ssp. *dendrolimi* had a significant impact on the pest (~30% larval mortality), followed by other braconids *G. liparidis* (13%) and *Cotesia* spp. (12%) (Figure 19B).

The tachinids, particularly *Masicera sphingivora*, *Tachina* sp., and *Blepharipa* spp., caused high mortality of *D. sibiricus* pupae (Figure 19C). Their combined contribution to pest mortality often reached up to 70% (Figure 19, the phase of population decline). In contrast, hymenopteran parasitoids that attack the pupae of *D. sibiricus*, such as *Iseropus stercorator*, *Habronyx heros*, *Pimpla instigator*, and *Therion giganteum*, collectively accounted for approximately 10% of the pest mortality (Figure 19).

## 4. Discussion

Our study presents new field data and summarizes the information on the parasitoid diversity of *D. sibiricus* based on the archival collections (since 1905) and the literature data (since 1940) for Northern Asia. Furthermore, it compiled data on parasitism in various regions of Siberia and the Russian Far East over the last 83 years.

Among parasitoids reared from eggs, larvae, and pupae of *D. sibiricus* from the recent pest foci in Siberia and investigated archival specimens (overall 860 parasitoid specimens), we identified 16 parasitoid species, including one braconid species (*Meteorus versicolor*) that was documented as a parasitoid of *D. sibiricus* for the first time. It is important to stress that species identification in our study was mostly based on the analysis of morphological features of parasitoids (the representative of orders Hymenoptera and Diptera). The exclusive reliance on DNA barcoding would not have been sufficient for determining the majority of these species given that hymenopteran and dipteran parasitoids, particularly from Asia, are generally underrepresented in genetic databases. Consequently, genetic databases such as BOLD and NCBI lack reference DNA barcodes for these taxonomic groups. In fact, among 16 species that we subjected to DNA barcoding from Siberia, which included nine hymenopteran and two dipteran parasitoids, 11 species were previously unrecorded in both BOLD and GenBank. The incorporation of DNA barcoding into our study, primarily rooted in morphological identification, has enabled the acquisition of reliable genetic data. This, in turn, contributes to the enhancement of existing genetic databases, facilitating more rapid and precise species identification [100,101]. Furthermore, it aids in the identification of misclassified cases in BOLD and/or NCBI [102,103].

The use of integrative taxonomy has allowed us to resurrect the Eastern Palaearctic form of *Aleiodes esenbeckii* [92] from synonymy as a valid subspecies, *Aleiodes esenbeckii* (Hartig, 1838) *dendrolimi* (Matsumura, 1926), status nov. This reclassification is supported by morphological and DNA barcoding data, which enable the reliable distinction of this subspecies from *Aleiodes esenbeckii* (referred to as *Aleiodes esenbeckii esenbeckii* in our study), found in the Western Palaearctic and associated with the related species, *D. pini* [92]. Furthermore, our genetic analysis has underscored the necessity of clarifying the intraspecific variability in certain species, in particular, *Trichogramma dendrolimi.* The Siberian specimens of this species exhibited significant genetic divergence from those found in China and Italy (2.6% and 2.9%, respectively), which may be a sign of cryptic diversity.

Based on the results of an extensive literature survey involving articles published in the last 83 years, we compiled an exhaustive list of parasitoids associated with *D. sibiricus* in its historical range Northern Asia (i.e., the Asian part of Russia), northern and north-eastern regions of Kazakhstan, Mongolia, China, and Korea. The compiled list comprises 93 species of Hymenoptera and Diptera. Remarkably, nearly all these parasitoids, with a few exceptions, are generalists that are associated with other Lepidoptera species and, in some cases, with representatives of other insect orders, such as Coleoptera, Hymenoptera, Hemiptera, and Diptera. This implies that these parasitoids are widely distributed across the *D. sibiricus* range and have the potential to switch to the pest when it is present in abundance. These parasitoids can exert an impact on host population dynamics, representing a form of “top-down” pressure from natural enemies [104]. However, the parasitoid response to host population growth usually has a time lag that may serve as a predictor of cyclical fluctuations in host abundance [104]. Indeed, the analysis of parasitism in the *D. sibiricus* population experiencing growth, outbreak, decline, and depression phases revealed a gradual, time-lagged increase in parasitism, with a peak during the pest decline phase. This phenomenon was observed in our study for egg and pupal parasitoids. However, no clear mechanism was identified for larval parasitoids. The complexes of larval parasitoids are generally taxonomically more diverse compared with egg and pupal parasitoid assemblages, but the majority of larval parasitoids play an insignificant role in the population dynamics of *D. sibiricus.*

Clearly, not all parasitoids associated with *D. sibiricus* possess the capacity to control its populations. Our field observations and the literature data on parasitism in *D. sibiricus* across Northern Asia over the last 83 years reveal that only eight parasitoids, constituting 8.6% of all parasitoid species associated with the pest, cause significant mortality in its populations. They are the hymenopteran parasitoids that attack *D. sibiricus* eggs (*Telenomus tetratomus* and *Ooencyrtus pinicolus*) and larvae (*Aleiodes esenbeckii* ssp. *dendrolimi*, *Cotesia* spp. and *G. liparidis*), and the dipteran pupal parasitoids (*Masicera sphingivora*, *Tachina* sp. and *Blepharipa* sp.) that cause high mortality in the pest populations (up to 100% in some cases). Indeed, both the literature data and our field observations have unveiled remarkable instances of exceptionally high parasitism in certain regions of Northern Asia. For example, the egg parasitoid *Telenomus tetratomus* was demonstrated to induce up to 100% egg mortality in *D. sibiricus* populations in various Siberian locations, including Irkutsk Province in 1950 [21] and Krasnoyarsk Territory in 1946 [18] and 2018 (our observations in the present paper). Additionally, such high parasitism was documented in the Russian Far East, particularly in Primorsky Territory in 1996 [24]. *Telenomus tetratomus* also demonstrated high efficacy in various Siberian regions. To the west, in Tomsk Province, it caused the mortality of 88% of the pest eggs in 1956 [19], and to the east, in Zabaikalsky Territory in 1940, it was responsible for 79% of the egg mortality [21]. In the Tuva Republic in the 1960s and 1970s, it killed up to 65% of *D. sibiricus* eggs [23].

*Telenomus* egg parasitoids have been largely reported as major enemies of insect pests, both native and invasive alien species, particularly, from the orders Lepidoptera and Hemiptera [105,106,107,108,109,110,111]. The representatives of this species-rich genus are often not easy to identify, and misidentifications are likely. Thus, a genus revision involving integrative taxonomy is highly needed, bearing in mind that *Telenomus* species are more and more used for biological control of forest and agricultural pests [105,106,107,108,109,110,111].

Among other parasitoids showing the ability to control *D. sibiricus* in Northern Asia, there are seven species, including tachinids (*Masicera sphingivora*, *Tachina* sp., and *Blepharipa* sp.) and hymenopteran parasitoids (*Iseropus stercorator*, *Habronyx heros*, *Pimpla instigator*, and *Therion giganteum*) that target *D. sibiricus* pupae (detailed examples are provided in Table S2). Their potential and suitability for serving as biocontrol agents should be further explored in order to develop effective programs to suppress the outbreaks of this important forest pest.

## 5. Conclusions

Our study points to a high taxonomic diversity of parasitoids associated with *D. sibiricus* in Northern Asia. To facilitate their swift and accurate identification and differentiation from the related species distributed across Eurasia, it is imperative to establish a reference DNA barcoding reference library for *D. sibiricus* parasitoids, providing data on intraspecific genetic variability within the pest range and beyond. The molecular genetic data acquired from the revised archival specimens and the parasitoids freshly reared from *D. sibiricus* eggs, larvae, and pupae in our study, which were initially identified by morphology, can serve as a robust foundation for the development of such a library.

Our research highlights an urgent need to revise the genus *Telenomus* involving integrative taxonomy, bearing in mind that its representatives are highly important biocontrol agents, and their use in biocontrol programs against plant pests is increasing.

A comparative study of the parasitoid complexes of *D. sibiricus* and the closely related *D. pini*, which naturally occurs in Europe and whose native range partially overlaps with *D. sibiricus* in Russia, is required. This research could help identify common parasitoid species, as well as those having the ability to switch from *D. pini* to *D. sibiricus*, exerting a notable control effect. Such knowledge is highly essential, especially considering the potential of *D. sibiricus* to spread westward.

The parasitoids enabling the significant mortality of *D. sibiricus* in Northern Asia should be further explored for the development of biocontrol programs. Among them, the parasitoids infesting *D. sibiricus* eggs (*Telenomus* and *Ooencyrtus* spp.), larvae (*Aleiodes esenbeckii* ssp. *dendrolimi*, *Cotesia* spp., and *Iseropus stercorator*), and pupae (the ichneumonids *Habronyx heros*, *Pimpla instigator*, *Therion giganteum*, and the tachinids *Masicera sphingivora*, and *Tachina* and *Blepharipa* spp.) should be given special attention.

## Figures and Tables

**Figure 7:**
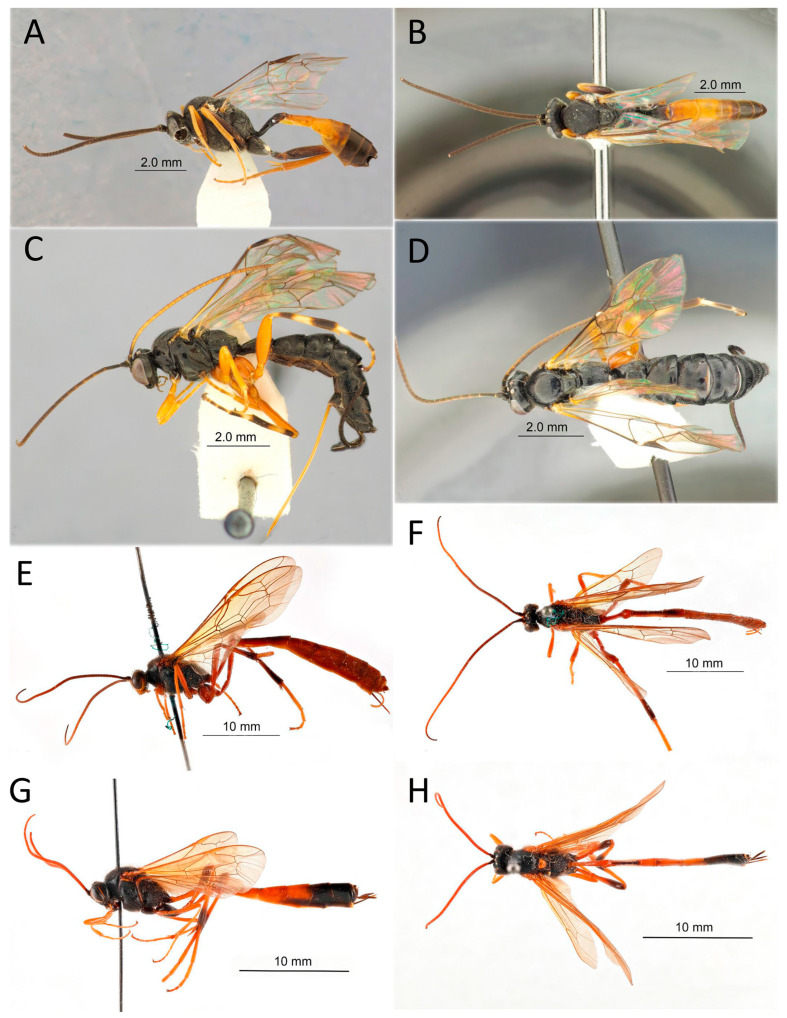
Parasitoids from the family Ichneumonidae, (**A**,**C**,**E**,**G**) lateral view and (**B**,**D**,**F**,**H**) dorsal view. (**A**,**B**) *Hyposoter validus* (Pfankuch) (Campopleginae), Tomsk Province; (**C**,**D**) *Iseropus stercorator* (Fabricius) (Pimplinae), Irkutsk Province; (**E**,**F**) *Habronyx heros* (Wesmael) (Anomaloninae), Krasnoyarsk Territory; and (**G**,**H**) *Therion circumflexum* (Linnaeus) (Anomaloninae), Krasnoyarsk Territory. Photos: S.A. Belokobylskij.

**Figure 11:**
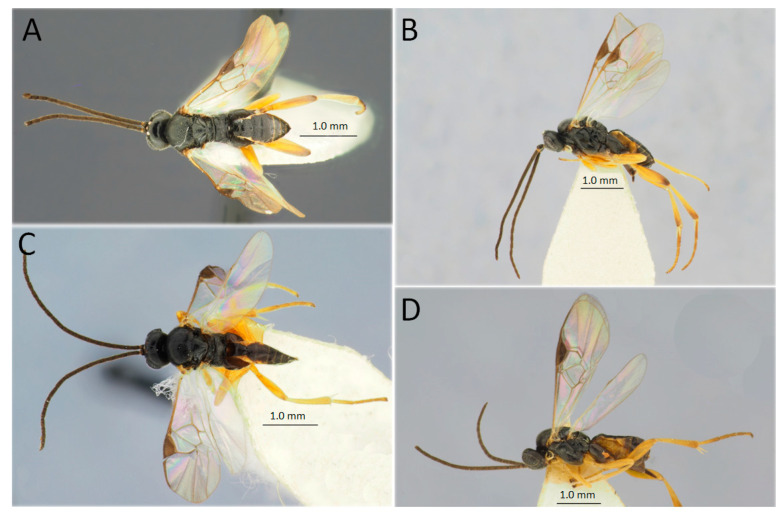
Parasitoids from the family Braconidae: (**A**,**C**) dorsal view and (**B**,**D**) lateral view. (**A**,**B**) *Cotesia ordinaria* (Ratzeburg) (Microgastrinae), Krasnoyarsk Territory and (**C**,**D**) *Glyptapanteles liparidis* (Bouché) (Microgastrinae), Irkutsk Province. Photos: S.A. Belokobylskij.

**Figure 12:**
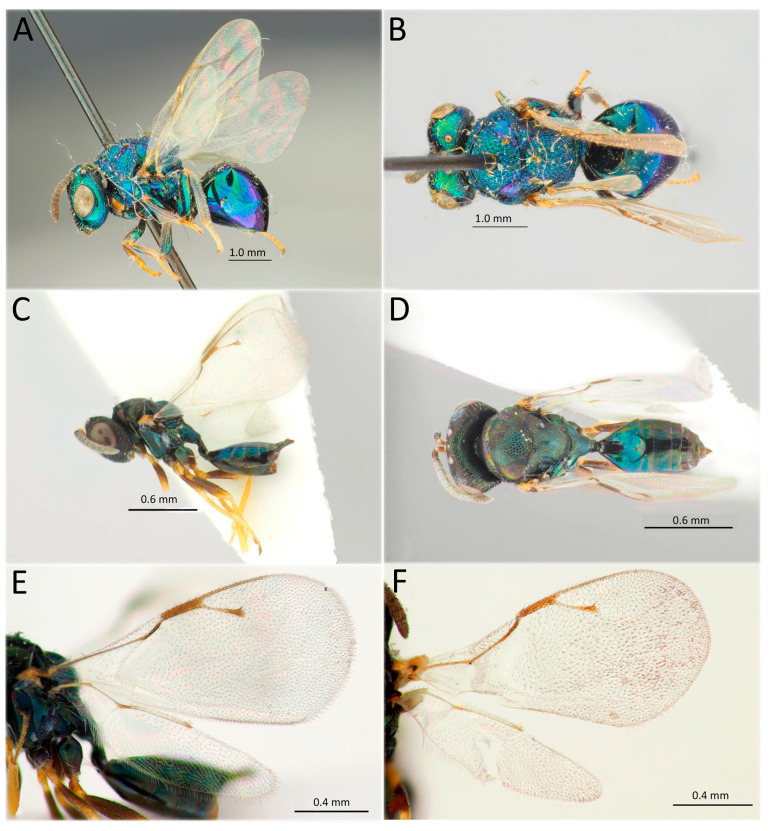
Parasitoids from the superfamily Chalcidoidea: (**A**,**C**) lateral view; (**B**,**D**) dorsal view; and (**E**,**F**) wings. (**A**,**B**) *Perilampus nitens* Walker (Perilampidae), Irkutsk Province and (**C**–**F**) *Pachyneuron solitarium* (Hartig) (Pteromalidae), ibidem. Photos: S.A. Belokobylskij.

**Figure 15:**
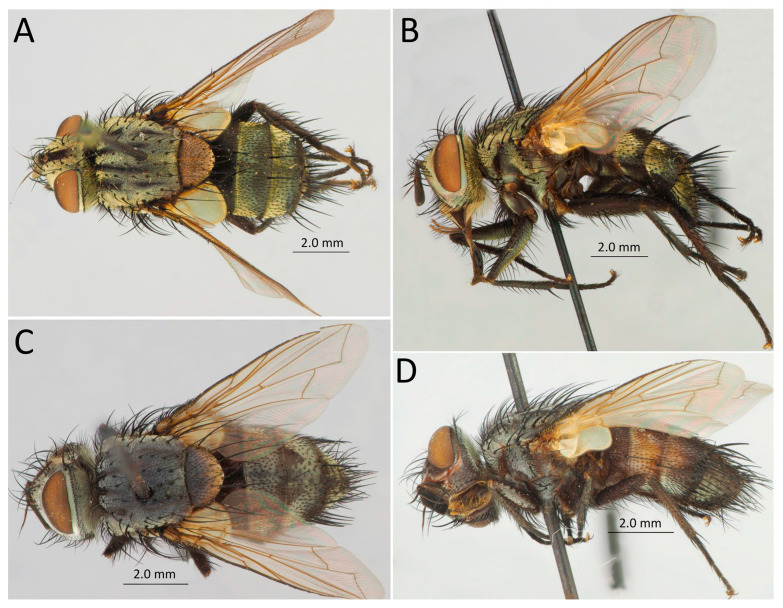
Parasitoids from the family Tachinidae: (**A**,**C**) dorsal view and (**B**,**D**) lateral view. (**A**,**B**) *Exorista larvarum* (Linnaeus) (Exoristinae) and (**C**,**D**) *Masicera sphingivora* (Robineau-Desvoidy) (Exoristinae). Photos: S.A. Belokobylskij.

**Table 1:** Intra- and interspecific divergences in the COI mtDNA gene among Braconidae (Hymenoptera) parasitoid species attacking *Dendrolimus sibiricus* in Northern Asia. Minimal pairwise distances are given for species pairs; maximal intraspecific distances are indicated in brackets; (—) indicates that no data are provided because a single specimen was sequenced.

Species ^1^	Species			
	*Aleiodes esenbeckii* ssp. *dendrolimi*, status nov.	*Aleiodes* *esenbeckii* (Spain)	*Cotesia* *ordinaria*	*Glyptapanteles* *liparidis*
*Aleiodes esenbeckii* ssp. *dendrolimi*, status nov.	(0.75)			
*Aleiodes esenbeckii* ssp. *esenbeckii* (Spain) ^2^	3.72	(—)		
*Cotesia ordinaria*	28.28	31.18	(1.80)	
*Glyptapanteles liparidis*	30.99	31.42	8.18	(0.92)

^1^ Species involved in the analyses were represented by the following number of replications: *Aleiodes esenbeckii* ssp. *dendrolimi* (13), *A. esenbeckii* (Spain) (1), *Cotesia ordinaria* (7), *G lyptapanteles liparidis* (3). ^2^ DNA barcode *A. esenbeckii* (Spain) was retrieved from BOLD for comparative analysis.

**Table 2:** Intraspecific divergences in the COI mtDNA gene in *Trichogramma dendrolimi* (Hymenoptera: Trichogrammatidae) from Western Siberia, Central Europe (Italy) and Eastern Asia (China). For an explanation of maximal distance and the meaning of (—), see the title of Table 1.

Origin of *T. dendrolimi*	Origin of *T. dendrolimi*		
	Western Siberia	Italy	China
Western Siberia	(2.05)		
Italy	2.62	(—)	
China	2.90	0.51	(—)

**Table 3:** Intra- and interspecific divergences in the COI mtDNA gene among Tachinidae (Diptera) parasitoids attacking *Dendrolimus sibiricus* in Northern Asia. For an explanation of minimal and maximal distances and the meaning of (—), see the title of Table 1.

Species ^1^	Species		
	*Masicera* *sphingivora*	*Masicera* *silvatica*	*Exorista* *larvarum*
*Masicera sphingivora*	(0.2)		
*Masicera silvatica * ^2^	3.3	(—)	
*Exorista larvarum*	12.2	13.8	(1.6)

^1^ Species involved in the analyses were represented by the following number of replications: *Masicera sphingivora* (5), *M. sylvatica* (1), *Exorista larvarum* (3). ^2^ DNA barcode of *M. silvatica* (France) was retrieved from BOLD for comparative analyses, as it was the nearest neighbour to *M. sphingivora* from Siberia.

**Table 4:** Most abundant * parasitoid species in *Dendrolimus sibiricus* populations in the Asian part of Russia and China.

No.	Parasitoid Species	Parasitized Stage ^1^	Region	References
	**HYMENOPTERA: Ichneumonidae**			
1	*Hyposoter takagii* (Matsumura, 1926)	L	China	[27]
2	*Apechthis capulifera* (Kriechbaumer, 1887)	P	RU: Khabarovsk Territory	[27]
3	*Pimpla disparis* Viereck, 1911	P	RU: Sakhalin, Kuril Isl.	[61]
4	*Theronia atalantae atalantae* (Poda, 1761)	P	RU: Tomsk, Novosibirsk Province, Tuva Rep.	[57]
	**Braconidae**			
5	*Aleiodes* (*Aleiodes*) *esenbeckii* (Hartig, 1838) ssp. *dendrolimi* (Matsumura, 1926)	L	RU: Tomsk, Novosibirsk Province, Tuva Rep; Khabarovsk Territory, Primorskiy Territory	[27,57]
6	*Cotesia ordinaria* (Ratzeburg, 1844) (*Apanteles dendrolimi* Mats.)	L	RU: Tomsk, Novosibirsk Province, Tuva Rep., Khabarovsk Trr., Primorskiy Territory	[27,57]
7	*Cotesia rubripes* (Haliday, 1834)	L	RU: Omsk, Tomsk, Novosibirsk Province, Tuva Rep.	[19,57]
	**Scelionidae**			
8	*Telenomus dendrolimi* (Matsumura, 1925)	E	RU: Sakhalin Province, Kuril Islands; China	[21,61]
9	*Telenomus tetratomus* (Thomson, 1861)	E	RU: Eastern Siberia, Far East	[24,57,61]
	**Torymidae**			
10	*Monodontomerus aeneus* (Fonscolombe, 1832)	L, P	RU: Siberia	[19]
	**Trichogrammatidae**			
11	*Trichogramma dendrolimi* Matsumura, 1926	E	RU: Tomsk, Novosibirsk Province, Tuva Rep.	[57]
12	*Trichogramma evanescens* Westwood, 1833	E	China	[61]
	**Encyrtidae**			
13	*Ooencyrtus pinicolus* (Matsumura, 1926)	E	RU: Khabarovsk Territory	[24,27]
	**Pteromalidae**			
14	*Pachyneuron solitarium* (Hartig, 1838)	E	RU: Far East	[61]
	**DIPTERA: Tachinidae**			
15	*Blepharipa pratensis* (Meigen, 1824)	L, P	RU: Siberia, Khabarovsk Territory, Primorskiy Territory	[24,27]
16	*Blepharipa schineri* (Mesnil, 1939)	L, P	RU: Khabarovsk Territory, Primorskiy Territory	[24,27]
17	*Carcelia matsukarehae* (Shima, 1969)	L, P	RU: Amur Province	[91]
18	*Lespesia frenchii* (Williston, 1889)	L, P	RU: Central, Eastern Siberia, Amur Province, Primorskiy Territory	[91]
19	*Masicera sphingivora* (Robineau-Desvoidy, 1830)	L, P	RU: Eastern Siberia, Khabarovsk Territory	[27]
	**Total**	E—6 spp., L and or P—13 spp.		

Remarks. ^1^ E—egg; L—larva, P—pupa; * Present in mass in some years and able to kill ≥ 50% of individuals in *D. sibiricus* populations.

## Data Availability

The genetic data used in this study are publicly accessible in BOLD using the link https://dx.doi.org/10.5883/DS-PARDS (accessed on 1 January 2024).

## Supplementary Materials

The following supporting information can be downloaded at: https://www.mdpi.com/article/10.3390/life14020268/s1, Table S1: The specimens of parasitoids reared from *Dendrolimus sibiricus* in Siberia (Russia) in 2018–2022 and the DNA barcodes of parasitoids borrowed from BOLD or GenBank for comparison; Table S2: Parasitism of eggs, larvae, and pupae of Dendrolimus sibiricus in accordance to the pest population dynamics in Northern Asia; Table S3: The checklist of parasitoids associated with *Dendrolimus sibiricus* in Northern Asia; Table S4: The predatory flies, with some exhibiting parasitoid behaviour, associated with the larvae and/or pupae of *Dendrolimus sibiricus* in Northern Asia; Table S5: The matrices of Z-values calculated with the use of the Mann–Whitney U-test for comparing the parasitism of *Dendrolimus sibiricus* eggs, larvae, and pupae at different population dynamics phases in Northern Asia.
