# Supplementary material for: The Diversity of Parasitoids and Their Role in the Control of the Siberian Moth, Dendrolimus sibiricus (Lepidoptera: Lasiocampidae), a Major Coniferous Pest in Northern Asia
Source: Life (Basel). 2024 Feb 17;14(2):268. doi: 10.3390/life14020268 (PMC10890493; doi:10.3390/life14020268)
Supplement: Supplementary file 1 [file life-14-00268-s001.zip › Table S1.pdf]

The Parasitoid Diversity and Their Role in the Control of the Siberian Moth, *Dendrolimus sibiricus* (Lepidoptera: Lasiocampidae), a Major Coniferous Pest in Northern Asia

Natalia I. Kirichenko, Alexander A. Ageev, Sergey A. Astapenko, Anna N. Golovina, Dmitry R. Kasparyan, Oksana V. Kosheleva, Alexander V. Timokhov, Ekaterina V. Tselikh, Evgeny V. Zakharov, Dmitrii L. Musolin, Sergey A. Belokobylskij

Table S1. The specimens of parasitoids reared from *Dendrolimus sibiricus* in Siberia (Russia) in 2018–2022 and the DNA barcodes of parasitoids borrowed from BOLD or GenBank for comparison.

No.	Sample ID	Family	Species	Country	Administrative region*	District	Collection Date	Collectors	Process ID	GenBank Accession Number
**ORIGINAL SEQUENCES**										
**HYMENOPTERA**										
1	NK1517	Braconidae	Aleiodes esenbeckii	Russia	Krasnoyarsk Terr.	Irbeiskiy district	30.IV.2020	Golovina A.N.	DSPAR033-22	OR732445
2	NK1516	Braconidae	Aleiodes esenbeckii	Russia	Krasnoyarsk Terr.	Irbeiskiy district	30.IV.2020	Golovina A.N.	DSPAR032-22	OR732433
3	NK1514	Braconidae	Aleiodes esenbeckii	Russia	Krasnoyarsk Terr.	Irbeiskiy district	30.IV.2020	Golovina A.N.	DSPAR030-22	OR732450
4	NK1513	Braconidae	Aleiodes esenbeckii	Russia	Tomsk Prov.	Shegarskiy district	19.IX.2018	Astapenko S.A.	DSPAR029-22	OR732462
5	NK1512	Braconidae	Aleiodes esenbeckii	Russia	Tomsk Prov.	Shegarskiy district	19.IX.2018	Astapenko S.A.	DSPAR028-22	OR732463
6	NK1511	Braconidae	Aleiodes esenbeckii	Russia	Irkutsk Prov.	Kachugskiy district	28.VII.2020	Ageev A.A	DSPAR027-22	OR732438
7	NK1510	Braconidae	Aleiodes esenbeckii	Russia	Tomsk Prov.	Shegarskiy district	19.IX.2018	Astapenko S.A.	DSPAR026-22	OR732424
8	NK1509	Braconidae	Aleiodes esenbeckii	Russia	Tomsk Prov.	Shegarskiy district	19.IX.2018	Astapenko S.A.	DSPAR025-22	OR732431

No.	Sample ID	Family	Species	Country	Administrative region*	District	Collection Date	Collectors	Process ID	GenBank Accession Number
9	NK1508	Braconidae	Aleiodes esenbeckii	Russia	Tomsk Prov.	Shegarskiy district	19.IX.2018	Astapenko S.A.	DSPAR024-22	OR732439
10	NK1501	Braconidae	Aleiodes esenbeckii	Russia	Krasnoyarsk Terr.	Irbeiskiy district	15.VI.2021	Astapenko S.A.	DSPAR017-22	OR732425
11	NK1500	Braconidae	Aleiodes esenbeckii	Russia	Krasnoyarsk Terr.	Irbeiskiy district	15.VI.2021	Astapenko S.A.	DSPAR016-22	OR732423
12	NK1499	Braconidae	Aleiodes esenbeckii	Russia	Tomsk Prov.	Asinovskiy district	21.IX.2018	Ageev A.A	DSPAR015-22	OR732429
13	NK1487	Braconidae	Aleiodes esenbeckii	Russia	Krasnoyarsk Terr.	Irbeiskiy district	26.VIII.2019	Astapenko S.A.	DSPAR003-22	OR732461
14	NK1507	Braconidae	Cotesia ordinaria	Russia	Irkutsk Prov.	Kachugskiy district	16.VII.2021	Ageev A.A	DSPAR023-22	OR732422
15	NK1506	Braconidae	Cotesia ordinaria	Russia	Irkutsk Prov.	Kachugskiy district	16.VII.2021	Ageev A.A	DSPAR022-22	OR732437
16	NK1505	Braconidae	Cotesia ordinaria	Russia	Krasnoyarsk Terr.	Irbeiskiy district	15.VI.2021	Astapenko S.A.	DSPAR021-22	OR732447
17	NK1504	Braconidae	Cotesia ordinaria	Russia	Krasnoyarsk Terr.	Irbeiskiy district	15.VI.2021	Astapenko S.A.	DSPAR020-22	OR732460
18	NK1503	Braconidae	Cotesia ordinaria	Russia	Krasnoyarsk Terr.	Irbeiskiy district	30.IV.2020	Golovina A.N.	DSPAR019-22	OR732459
19	NK1502	Braconidae	Cotesia ordinaria	Russia	Krasnoyarsk Terr.	Irbeiskiy district	30.IV.2020	Golovina A.N.	DSPAR018-22	OR732454
20	NK1490	Braconidae	Glyptapanteles liparidis	Russia	Irkutsk Prov.	Kachugskiy district	16.VII.2021	Ageev A.A	DSPAR006-22	OR732467
21	NK1489	Braconidae	Glyptapanteles liparidis	Russia	Irkutsk Prov.	Kachugskiy district	16.VII.2021	Ageev A.A	DSPAR005-22	OR732457
22	NK1493	Encyrtidae	Ooencyrtus pinicolus	Russia	Irkutsk Prov.	Kachugskiy district	28.VII.2020	Ageev A.A	DSPAR009-22	OR732420
23	NK1492	Encyrtidae	Ooencyrtus pinicolus	Russia	Irkutsk Prov.	Kachugskiy district	28.VII.2020	Ageev A.A	DSPAR008-22	OR732436

No.	Sample ID	Family	Species	Country	Administrative region*	District	Collection Date	Collectors	Process ID	GenBank Accession Number
24	NK1573	Encyrtidae	Ooencyrtus pinicolus	Russia	Tyva Rep.	Targalovka location	09.VIII.1963	Kondakov Yu.P.	DSPAR089-22	OR732426
25	NK1570	Encyrtidae	Ooencyrtus pinicolus	Russia	Tyva Rep.	Targalovka location	09.VIII.1963	Kondakov Yu.P.	DSPAR086-22	OR732444
26	NK1568	Encyrtidae	Ooencyrtus pinicolus	Russia	Tyva Rep.	Targalovka location	09.VIII.1963	Kondakov Yu.P.	DSPAR084-22	OR732442
27	NK1563	Encyrtidae	Ooencyrtus pinicolus	Russia	Tyva Rep.	Targalovka location	09.VIII.1963	Kondakov Yu.P.	DSPAR079-22	OR732458
28	NK1486	Ichneumonidae	Habronyx heros	Russia	Krasnoyarsk Terr.	Irbeiskiy district	06.IX.2019	Astapenko S.A.	DSPAR002-22	OR732464
29	NK1498	Ichneumonidae	Hyposoter validus	Russia	Tomsk Prov.	Asinovskiy district	21.IX.2018	Ageev A.A	DSPAR014-22	OR732452
30	NK1488	Ichneumonidae	Hyposoter validus	Russia	Tomsk Prov.	Asinovskiy district	21.IX.2018	Ageev A.A	DSPAR004-22	OR732449
31	NK1515	Perilampidae	Perilampus nitens	Russia	Krasnoyarsk Terr.	Irbeiskiy district	15.VII.2020	Ageev A.A	DSPAR031-22	OR732441
32	NK1524	Pteromalidae	Pachyneuron solitarium	Russia	Irkutsk Prov.	Kachugskiy district	29.VII.2020	Astapenko S.A.	DSPAR040-22	OR732430
33	NK1494	Pteromalidae	Pachyneuron solitarium	Russia	Irkutsk Prov.	Kachugskiy district	28.VII.2020	Ageev A.A	DSPAR010-22	OR732440
34	NK1491	Pteromalidae	Pachyneuron solitarium	Russia	Irkutsk Prov.	Kachugskiy district	28.VII.2020	Ageev A.A	DSPAR007-22	OR732443
35	NK1523	Scelionidae	Telenomus tetratomus	Russia	Irkutsk Prov.	Kachugskiy district	28.VII.2020	Astapenko S.A.	DSPAR039-22	OR732427
36	NK1518	Scelionidae	Telenomus tetratomus	Russia	Irkutsk Prov.	Kachugskiy district	28.VII.2020	Ageev A.A	DSPAR034-22	OR732448
37	NK1497	Scelionidae	Telenomus tetratomus	Russia	Irkutsk Prov.	Kachugskiy district	28.VII.2020	Ageev A.A	DSPAR013-22	OR732428
38	NK1496	Scelionidae	Telenomus tetratomus	Russia	Irkutsk Prov.	Kachugskiy district	28.VII.2020	Ageev A.A	DSPAR012-22	OR732446

No.	Sample ID	Family	Species	Country	Administrative region*	District	Collection Date	Collectors	Process ID	GenBank Accession Number
39	NK1522	Trichogram-matidae	Trichogramma dendrolimi	Russia	Irkutsk Prov.	Kachugskiy district	28.VII.2020	Astapenko S.A.	DSPAR038-22	OR732453
40	NK1521	Trichogram-matidae	Trichogramma dendrolimi	Russia	Irkutsk Prov.	Kachugskiy district	28.VII.2020	Astapenko S.A.	DSPAR037-22	OR732455
41	NK1520	Trichogram-matidae	Trichogramma dendrolimi	Russia	Irkutsk Prov.	Kachugskiy district	28.VII.2020	Astapenko S.A.	DSPAR036-22	OR732435
42	NK1519	Trichogram-matidae	Trichogramma dendrolimi	Russia	Irkutsk Prov.	Kachugskiy district	28.VII.2020	Astapenko S.A.	DSPAR035-22	OR732466
					**DIPTERA**					
43	NK1526	Tachinidae	Exorista larvarum	Russia	Tyva Rep.	Ishtii-Khem village	01.VIII.1963	Kondakov Yu.P.	DSPAR042-22	OR732432
44	NK1531	Tachinidae	Masicera sphingivora	Russia	Tyva Rep.	Ishtii-Khem village	18.III.1964	Kondakov Yu.P.	DSPAR047-22	OR732434
45	NK1530	Tachinidae	Masicera sphingivora	Russia	Tyva Rep.	Ishtii-Khem village	15.IV.1964	—	DSPAR046-22	OR732456
46	NK1529	Tachinidae	Masicera sphingivora	Russia	Tyva Rep.	Ishtii-Khem village	20.III.1964	—	DSPAR045-22	OR732421
47	NK1528	Tachinidae	Masicera sphingivora	Russia	Tyva Rep.	Ishtii-Khem village	13.III.1964	Kondakov Yu.P.	DSPAR044-22	OR732451
48	NK1527	Tachinidae	Masicera sphingivora	Russia	Tyva Rep.	Ishtii-Khem village	18.III.1964	—	DSPAR043-22	OR732465
					**SEQUENCES BORROWED FROM BOLD OR GENBANK**					
					**HYMENOPTERA**					
49	KU682240	Braconidae	Aleiodes esenbeckii	Spain	—	—	—	—	GBMIN74556-17	KU682240
50	MRS_JFT0716	Braconidae	Cotesia sp.	United Kingdom	—	—	15.VI.2016	Shaw M.R.	BCNCA261-18	OR732419

No.	Sample ID	Family	Species	Country	Administrative region*	District	Collection Date	Collectors	Process ID	GenBank Accession Number
51	KY421536	Braconidae	Glyptapanteles liparidis	Czech Rep.	—	—	—	—	GBMIN74375-17	KY421536
52	MN521025	Encyrtidae	Ooencyrtus sp. SAEVG Morph0251	India	—	—	01.X.2015	—	GBMNC44807-20	MN521025
53	INDOBIOSYS-CCDB25314-H11	Perilampidae	—	Indonesia	Jawa Barat	—	05.X.2015	Schmidt S.	BCIND760-16	MH926749
54	BIOUG24698-E09	Pteromalidae	—	Canada	Ontario	Guelph	26.VIII.2014	Meredith G.	GMOUF696-15	MG380662
55	BIOUG17132-E12	Scelionidae	—	Canada	Northwest Terr.	Nahanni Nat. Park	04.VII.2014	—	CNNHB2693-14	MG509194
56	BIOUG17632-B12	Scelionidae	—	Canada	Northwest Terr.	Nahanni Nat. Park	08.VIII.2014	—	CNNHG1972-14	KR792756
57	MG932197	Trichogram-matidae	Trichogramma dendrolimi	Italy	—	—	—	—	GBMNA19717-19	MG932197
58	MG932179	Trichogram-matidae	Trichogramma dendrolimi	China	—	—	01.I.1992	—	GBMNA19713-19	MG932179
					**DIPTERA**					
59	AB700013	Tachinidae	Exorista larvarum	Japan	Hokkaido-chiho	Hokkaido	—	—	GBDP14050-13	AB700013
60	BIOUG05974-D07	Tachinidae	Exorista larvarum	Canada	Saskatchewan	Prince Albert National Park	14.VII.2012	—	SSPAA2893-13	KM913279
61	JP00410	Tachinidae	Masicera silvatica	France	Provence-Alpes-Cote d'Azur	Hautes-Alpes	16.VII.2009	Tschorsnig	TACFI580-12	KX843725

No.	Sample ID	Family	Species	Country	Administrative region*	District	Collection Date	Collectors	Process ID	GenBank Accession Number
**OUTGROUPS**										
1	NK258	Tenthredinidae	Profenusa thomsoni	Russia	Novosibirsk Prov.	Central Siberian Bot. garden	10.VII.2012	Kirichenko N.	ISSIK122-14	KX360278
2	NK261	Agromyzidae		Russia	Novosibirsk Prov.	Central Siberian Bot. garden	05.VIII.2011	Kirichenko N.	ISSIK125-14	OP292611

Remarks: *States: Terr. – Territory, Prov. – Province, Rep. – Republic; — data are not available.
