# Supplementary material for: The Diversity of Parasitoids and Their Role in the Control of the Siberian Moth, Dendrolimus sibiricus (Lepidoptera: Lasiocampidae), a Major Coniferous Pest in Northern Asia
Source: Life (Basel). 2024 Feb 17;14(2):268. doi: 10.3390/life14020268 (PMC10890493; doi:10.3390/life14020268)
Supplement: Supplementary file 1 [file life-14-00268-s001.zip › Table S2.pdf]

The Parasitoid Diversity and Their Role in the Control of the Siberian Moth, *Dendrolimus sibiricus* (Lepidoptera: Lasiocampidae), a Major Coniferous Pest in Northern Asia

Natalia I. Kirichenko, Alexander A. Ageev, Sergey A. Astapenko, Anna N. Golovina, Dmitry R. Kasparyan, Oksana V. Kosheleva, Alexander V. Timokhov, Ekaterina V. Tselikh, Evgeny V. Zakharov, Dmitrii L. Musolin, Sergey A. Belokobylskij

Table S2. Parasitism of eggs, larvae, and pupae of *Dendrolimus sibiricus* in accordance to its population dynamics phases in Northern Asia.

I. EGG PARASITISM

**Administrative region[1], sampling year**	**Number of collected eggs**	**Parasitoid contribution to *D. sibiricus* egg mortality, %**						**References[2]**
		Telenomus tetratomus	***Trichogramma dendrolimi***	***Trichogramma sp.***	***Mesopolobus subfumatus***	***Ooencyrtus pinicolus***	**Total parasitism**	
D. sibiricus population growth								
Tuva Rep., 1962	2466	19.20	0	0.08	0	0.08	19.36	[23]
Krasnoyarsk Ter., 2019	3099	5.70	0	0.08	0	0	5.78	Our paper, 2024
Buryatia Rep., 1958	3459	0.08	0.02	0	0	0	0.10	[21]
Amur Prov., 1982	2000	44.30	5.00	0	0	0.70	50.00	[20]
Primorsky Ter., 1991	N/A	19.00	0	0	0	0	19.00	[20]
D. sibiricus outbreak								
Tomsk Prov., 1955	N/A	N/A	N/A	N/A	N/A	N/A	16.60	[20]
Tomsk Prov., 1956	18727	88.42	0.81	0.02	0	0.05	89.30	[19]
Tomsk Prov., 1956	16737	59.90	16.90	0.02	0	0.01	76.83	[19]
Tomsk Prov., 1956	N/A	50.60	0	0	0	0	50.60	[19]
Tomsk Prov., 2018	N/A	30.00	0	0	0	0	30.00	Our paper, 2024
Krasnoyarsk Ter., 1946	87885	94.00	0	0	0	0	94.00	[18]
Krasnoyarsk Ter., 1965	4805	4.70	0	0	0	0.30	5.00	[21]

Administrative region[1], sampling year	Number of collected eggs	Parasitoid contribution to *D. sibiricus* egg mortality, %						References[2]
		Telenomus tetratomus	*Trichogramma dendrolimi*	*Trichogramma sp.*	*Mesopolobus subfumatus*	*Ooencyrtus pinicolus*	Total parasitism	
Krasnoyarsk Ter., 1966	5018	6.80	0	0	0	2.13	8.93	[21]
Krasnoyarsk Ter., 1995	N/A	N/A	N/A	N/A	N/A	N/A	32.00	[14]
Krasnoyarsk Ter., 2017	4652	80.00	3.10	0	0	0	83.10	Our paper, 2024
Krasnoyarsk Ter., 2018	N/A	100.00	0	0	0	0	100.00	Our paper, 2024
Tuva Rep., 1947	N/A	17.70	0	0	0	0	17.70	[19]
Tuva Rep., 1958	N/A	N/A	N/A	N/A	N/A	N/A	54.40	[20]
Tuva Rep., 1959	29090	71.67	1.74	0	0	2.04	81.93	[19]
Tuva Rep., 1960	8480	45.93	2.28	0	0	4.10	52.31	[19]
Tuva Rep., 1963	1689	16.70	0	0.10	0	0.10	16.90	[23]
Tuva Rep., 1964	180315	52.70	0	0.20	0	0.08	52.98	[23]
Tuva Rep., 1965	19593	52.00	0	0.10	0	0.10	52.20	[23]
Tuva Rep., 1966	391	65.50	0	0	0	0	65.50	[23]
Irkutsk Prov., 1949	3841	64.07	0	0.13	0	0.07	64.27	[21]
Irkutsk Prov., 1951	768	75.78	3.5	0	0	5.08	84.36	[21]
Irkutsk Prov., 2020	7500	54.00	0	38.00	0	0	92.00	Our paper, 2024
Irkutsk Prov., 2022	13723	14.40	9.80	0	0	0	24.20	Our paper, 2024
Buryatia Rep., 1967	4640	N/A	N/A	N/A	N/A	N/A	98.60	[21]
Amur Prov., 1983	5000	45.00	5.00	0	0	10	60	[20]
Amur Prov., 1957	4500	43.50	5.00	0	0	1.50	50	[20]
Primorsky Ter., 1975	6000	0.10	0	0	0	51.00	51.18	[20]
Primorsky Ter., 1986	N/A	2.00	1.00	0	0	61.00	64.00	[20]
D. sibiricus population decline								
Tomsk Prov., 1956	N/A	N/A	N/A	N/A	N/A	N/A	81.90	[20]
Tomsk Prov., 2018	N/A	78.00	0	0	0	0	78.00	Our paper, 2024
Krasnoyarsk Ter., 1957	N/A	N/A	N/A	N/A	N/A	N/A	99.00	[21]
Krasnoyarsk Ter., 1996	N/A	80.90	0	0	0	0	80.90	[14]
Krasnoyarsk Ter., 2020	182	54.00	0	0	0	0	54.00	Our paper, 2024

Administrative region[1], sampling year	Number of collected eggs	Parasitoid contribution to *D. sibiricus* egg mortality, %						References[2]
		Telenomus tetratomus	*Trichogramma dendrolimi*	*Trichogramma sp.*	*Mesopolobus subfumatus*	*Ooencyrtus pinicolus*	Total parasitism	
Tuva Rep., 1948	N/A	56.00	0	0	0	0	56.00	[19,23]
Tuva Rep., 1950	14825	7.22	0.79	0.02	0	0.65	8.68	[19,23,55]
Tuva Rep., 1959	N/A	N/A	N/A	N/A	N/A	N/A	90.30	[20]
Irkutsk Prov., 1950	N/A	100.00	0	0	0	0	100.00	[21]
Irkutsk Prov., 1954	1964	6.90	0.20	0	0	0	7.10	[21]
Irkutsk Prov., 1955	918	18.51	0.65	0	0	0	19.16	[21]
Chita Prov., 1940	N/A	79.00	0	0	0	0	79.00	[21]
Amur Prov., 1990	8000	74.00	5.00	0	0	20.0	99.00	[20]
Amur Prov., 1958	4500	80	17.00	0	0	2.00	99.00	[21]
Rus. Far East, 1960	N/A	0	0	0	0	90	90	[21]
Primorsky Ter., 1976	12000	15.00	0	0	0	73.00	95.4	[20]
Primorsky Ter., 1996	N/A	93.00	0	0	0	0	93.00	[20]
D. sibiricus population depression								
Tomsk Prov., 1957	N/A	N/A	N/A	N/A	N/A	N/A	15.00	[20]
Tomsk Prov., 1962	16	62.50	0	0	0	0	62.50	[19]
Tuva Rep., 1961	246	36.18	0	0	0	11.38	47.56	[23]
Irkutsk Prov.1940	N/A	N/A	N/A	N/A	N/A	N/A	5.00	[21]

Remarks: [1]Ter. – Territory, Prov. – Province, Rep. – Republic; N/A – data not available. [2]See the reference list in our paper.

II. LARVA PARASITISM

Administrative region[1], sampling year	Number of collected larvae	Parasitoid contribution to *D. sibiricus* larval mortality, %						References[2]
		*Aleiodes esenbeckii**	*Hyposoter validus*	*Cotesia ordinaria*	*Glyptapanteles liparidis*	*Cotesia rubripes* (= *Apanteles rubripes)*	Total parasitism	
D. sibiricus population growth								
Tomsk Prov., 1955	N/A	N/A	N/A	N/A	N/A	24.60	25.10	[19,20]
Tuva Rep., 1958	N/A	N/A	N/A	N/A	N/A	N/A	0.50	[20]
Buryatia Rep., 1958	N/A	30.00	0	0	0	0	30.00	[21]
D. sibiricus outbreak								
Krasnoyarsk Ter., 1995	N/A	24.00	0	0	0	0	24.00	[14]
Buryatia Rep., 1959	N/A	41.00	0	0	0	0	41.00	[21]
Buryatia Rep., 1960	N/A	65.00	0	0	0	0	65.00	[21]
Irkutsk Prov., 2022	517	0	0	0.08	0	0	0.08	Our data 2023
Rus. Far East, 1962	N/A	10	0	7.00	0	0	17.00	[20]
Amur Prov., 1990	N/A	0	0	0	75.00	0	75.00	[20]
D. sibiricus population decline								
Tomsk Prov., 1956	851	10.40	0.30	0	0.20	0	10.90	[19,20]
Tomsk Prov., 2018	N/A	5.00	0	20	0	0	25.00	Our data 2023
Krasnoyarsk Ter., 1946	560	34.00	0	0	0	0	34.00	[55]
Krasnoyarsk Ter., 1965	93	3.30	0	23.70	0	0	27.00	[21]
Krasnoyarsk Ter., 1996	N/A	12.00		0.80	0	0	12.80	[14]
Tuva Rep., 1950	1698	0.30	1.00	0.02	0	0	1.32	[19]
Irkutsk Prov., 2021	N/A	6.20	0	2.10	0	0	8.30	Our data 2023
D. sibiricus population depression								
Tomsk Prov., 1957	162	0	0	0.60	0.60	0	1.20	[20]
Tomsk Prov., 1962	4	0	0	0	25.00	0	25.00	[19]

Administrative region[1], sampling year	Number of collected larvae	Parasitoid contribution to *D. sibiricus* larval mortality, %						References[2]
		*Aleiodes esenbeckii**	*Hyposoter validus*	*Cotesia ordinaria*	*Glyptapanteles liparidis*	*Cotesia rubripes* (= *Apanteles rubripes)*	**Total parasitism**	
Krasnoyarsk Ter., 1966	N/A	22.50	0	0	0	0	22.50	[21]
Tuva Rep., 1958	246	0.50	1.50	0	0.50	0	2.50	[19]
Tuva Rep., 1959	250	0.80	2.40	0	0.40	0	3.60	[19]
Tuva Rep., 1960	100	0	0	2.00	1.00	0	3.00	[19]
Irkutsk Prov., 1949	1321	63.97	0	0	0	0	63.97	[21]

Remarks: [1]Ter. – Territory, Prov. – Province, Rep. – Republic; N/A – data not available; * Likely *Aleiodes esenbeckii* ssp. *dendrolimi.* [2]See the reference list in our paper.

III. PUPA PARASITISM

Administrative region[1], sampling year	Number of collected pupae	Parasitoid contribution to *D. sibiricus* pupal mortality, %										References[2]
		Masicera Sphingivora (=M. zimini)	*Agria affinis (= Pseudosarcophaga affinis)*	*Exorista sp.*	*Therion giganteum (=Exochilum giganteum)*	*Iseropus stercorator*	*Pimpla instigator*	*Habronyx heros*	*Blepharipa* spp.	*Tachina sp.*	Total parasitism	
D. sibiricus population growth												
Tomsk Prov., 1955	N/A	N/A	N/A	N/A	N/A	N/A	N/A	N/A	N/A	N/A	13.30	[20]
Tuva Rep., 1958	N/A	N/A	N/A	N/A	N/A	N/A	N/A	N/A	N/A	N/A	3.90	[20]
Buryatia Rep., 1958	N/A	1.36	0	0	0	0	0	0	0	0	1.36	[21]
Primorsky Ter., 1985	N/A	0	0	0	0	0	0	0	10	0	10	[20]
D. sibiricus outbreak												
Tomsk Prov., 1956	712	0	2.00	0.30	0.50	0.60	0.10	0.40	0.90	0	4.80	[19]
Krasnoyarsk Ter., 1957	N/A	20.00	0	0	0	0	0	0	0	0	20.00	[21]
Tuva Rep.,1950	12000	66.60	0	0	0	0	0	0	0	0	66.60	[19]
Tuva Rep., 1958	N/A	5.20	0	0	0	0	0	0	0	0	5.20	[19]
Tuva Rep., 1959	N/A	21.70	0	0	0	0	0	0	0	0	21.70	[19]
Tuva Rep., 1960	1225	54.80	2.10	0.10	0.20	0.10	0	0	0	0	57.30	[19,23]
Irkutsk Prov., 1949	646	26.24	0	0	0	0	0	0	0	0	26.24	[21]
Irkutsk Prov., 2021	N/A	0	0	0	0.10	0	0	0	0	0	0.10	Our data 2023
Irkutsk Prov., 2022	679	0	0	0	0	0.58	0	0	0	41.64	42.22	Our data 2023
Buryatia Rep., 1960	N/A	1.30	0	0	0	0	0	0	0	0	1.30	[21]
Primorsky Ter., 1986	N/A	0	0	0	0	0	0	0	40	0	40.00	[20]
D. sibiricus population decline												
Krasnoyarsk Ter., 1963	18	0	0	0	0	11.10	0	0	0	61.11	72.21	[21]
Krasnoyarsk Ter.,	93	0	0	0	0	1.10	0	0	0	9.60	10.70	[21]

Administrative region[1], sampling year	Number of collec-ted pupae	Parasitoid contribution to *D. sibiricus* pupal mortality, %										References[2]
		Masicera Sphingi-vora (=M. zimini)	*Agria affinis (= Pseudo-sarcopha-ga affinis)*	*Exorista sp.*	*Therion giganteum (=Exochilum giganteum)*	*Iseropus sterco-rator*	*Pimpla instiga-tor*	*Habro-nyx heros*	*Blepha -ripa* spp.	*Tachina sp.*	**Total para-sitis m**	
1965												
Tuva Rep., 1950	7697	58.50	2.10	0.20	0	0.30	0.01	0	0	0	61.11	[55]
Irkutsk Prov.,1948	2944	N/A	N/A	N/A	N/A	N/A	N/A	N/A	N/A	N/A	35.11	[21]
Primorsky Ter., 1976	N/A	0	0	0	0	0	0	0	70	0	70	[20]
Primorsky Ter., 1987	N/A	0	0	0	0	0	0	0	60	0	60	[20]
D. sibiricus population depression												
Tomsk Prov., 1957	N/A	N/A	N/A	N/A	N/A	N/A	N/A	N/A	N/A	N/A	58.10	[20]
Tomsk Prov., 1957	240	0	9.20	6.70	7.10	1.70	0.80	12.10	17.90	0	55.50	[19]
Tuva Rep., 1960	N/A	N/A	N/A	N/A	N/A	N/A	N/A	N/A	N/A	N/A	60.90	[20]
Tuva Rep., 1961	119	31.20	1.70	2.50	2.50	0	0	0	0	0	37.90	[19]
Irkutsk Prov.,1949	N/A	31.00	0	0	0	0	0	0	0	0	31.00	[21]

Remarks: [1]Ter. – Territory, Prov. – Province, Rep. –Republic; N/A – data not available. [2]See the reference list in our paper.
