# Supplementary material for: The Diversity of Parasitoids and Their Role in the Control of the Siberian Moth, Dendrolimus sibiricus (Lepidoptera: Lasiocampidae), a Major Coniferous Pest in Northern Asia
Source: Life (Basel). 2024 Feb 17;14(2):268. doi: 10.3390/life14020268 (PMC10890493; doi:10.3390/life14020268)
Supplement: Supplementary file 1 [file life-14-00268-s001.zip › Table S3.pdf]

The Parasitoid Diversity and Their Role in the Control of the Siberian Moth, *Dendrolimus sibiricus* (Lepidoptera: Lasiocampidae), a Major Coniferous Pest in Northern Asia

Natalia I. Kirichenko, Alexander A. Ageev, Sergey A. Astapenko, Anna N. Golovina, Dmitry R. Kasparyan, Oksana V. Kosheleva, Alexander V. Timokhov, Ekaterina V. Tselikh, Evgeny V. Zakharov, Dmitrii L. Musolin, Sergey A. Belokobylskij

Table S3. The checklist of parasitoids associated with *Dendrolimus sibiricus* in Northern Asia.

No.	Species of parasitoids[1]	Hosts range of parasitoids[2]	Abundance of parasitoids	Distribution	References[3]
		**EGG PARASITOIDS**			
	**Hymenoptera: Encyrtidae**				
1.	*Ooencyrtus pinicolus* (Matsumura, 1926)	*Cosmotriche lobulina* (Denis et Schiffermüller), *Euthrix potatoria* (Linnaeus), *Dendrolimus superans* (Butler), *D. pini* (Linnaeus), *D. sibiricus* (Tschetverikov) (Lasiocampidae), Bombycidae, Erebidae (Lepidoptera)	Rare (Siberia), abundant (Far East)	Russia (South Ural, Western and Eastern Siberia, Far East), Kazakhstan, China, Japan	[19,27,36,37,47,50,54,56,57,61,81,85,87,90,91]
	**Hymenoptera: Scelionidae**				
2.	*Telenomus dendrolimi* (Matsumura,1925) (=*T. dendrolimusi* Chu, 1937)	*Dendrolimus sibiricus*, *Dendrolimus spectabilis* Butler, *D. albolineatus* Matsumura, *D. punctatus* Walker (Lasiocampidae, Lepidoptera)	Mass (56–94% Sakhalin, Kuril Islands, China)	Russia (Sakhalin, Kuril Islands), Japan, China, Korea	[47,61,76,91]
3.	*Telenomus tetratomus* (Thomson, 1861) (=*T. bombycis* Mayr, 1879, *T. gracilis* Mayr, 1879, *T. verticillatus* Kieffer, 1917)	*Dendrolimus pini*, *D. sibiricus*, *Macrothylacia rubi* (Linnaeus), *Lasiocampa trifolii* (Denis et Schiffermüller, **Euthrix potatoria*, **Eriogaster lanestris* (Linnaeus) (Lasiocampidae), *Calliteara abietis* (Denis et Schiffermüller) and *Orgyia antiqua* (Linnaeus) (Erebidae)	Rare (Buryatia), abundant (Krasnoyarsk), 50-80% (Amur)	Russia (European part, Western and Eastern Siberia, Far East), Europe, Kazakhstan, Mongolia, China, Japan, North America	[19,27,49,50,52–54,56,57,75,81,85,91]
4.	*Telenomus umbripennis* Mayr, 1879	*Dendrolimus segregatus* Butler, *Dendrolimus sibiricus* (Lasiocampidae, Lepidoptera)	No data	Russia (Siberia), Europe	[43,45,46,49,85]

No.	Species of parasitoids[1]	Hosts range of parasitoids[2]	Abundance of parasitoids	Distribution	References[3]
	**Hymenoptera: Trichogrammatidae**				
5.	*Trichogramma dendrolimi* Matsumura, 1926	*Dendrolimus pini, D. punctatus, D. sibiricus, D. spectabilis, Lebeda nobilis* Walker, *Adela nobilis* Christoph, *Malacosoma neustria* (Linnaeus) (Lasiocampidae, Lep), *Deporaus betulae* (Linnaeus)) (Rhynchitidae, Coleoptera), *Acantholyda posticalis* Matsumura (Pamphiliidae, Hymenoptera)	Abundant	Russia (European part, Western, Eastern Siberia, Far East), Europe, Turkey, Iran, Pakistan, Kazakhstan, China, Korean Peninsula, Japan, India, southeast Asia, South America	[19,54,56,57,85]
6.	*Trichogramma evanescens* Westwood, 1833	Lepidoptera varia (incl. Lasiocampidae), Chrysomelidae, Curculionidae, Dermestidae, Rhynchitidae Tenebrionidae (Coleoptera), Anthomyiidae, Stratiomyiidae, Syrphidae, Tabanidae (Diptera).	Abundant (China)	Russia (European part, Western Siberia), Europe, North Africa, Georgia, Armenia, Azerbaijan, Turkey, Iran, Turkmenistan, Uzbekistan, Kazakhstan, China, North America, India, southeast Asia, South America	[61,85]
7.	*Trichogramma lingulatum* Pang et Chen, 1974	*Dendrolimus sibiricus* (Lasiocampidae) and *Samia cynthia* (Drury) (Saturniidae) (Lepidoptera)	Rare	Russia (Amur), China, Japan	[24,27,85,90,91]
8.	*Trichogramma semblidis* (Aurivillius, 1898)	Lepidoptera varia (incl. *Dendrolimus sibiricus* (Lasiocampidae)), Rhynchitidae, Curculionidae (Coleoptera), Anthomyiidae, Sciomyzidae and Tabanidae (Diptera)	No data	Russia (European part, Western Siberia), Europe, Syria, Iran, Kazakhstan, North America, India	[19,85]
	**Hymenoptera: Eupelmidae**				
9.	*Anastatus japonicus* Ashmead, 1904 *	Lepidoptera varia (incl. *Dendrolimus sibiricus, Lymantria dispar* (Linnaeus) (Lasiocampidae)), Pentatomidae, Alydidae (Hemiptera); **parasitoids hosts** (*Aleiodes esenbeckii* (Hartig) ssp. *dendrolimi* (Braconidae, Hymenoptera), *Ooencyrtus pityocampae* Mercet (Encyrtidae, Hymenoptera))	Rare	Russia (European part, Ural, Eastern Siberia, Far East), Europe, North Africa, Turkey, Uzbekistan, Kyrgyzstan, Kazakhstan, China , Korean Peninsula, Japan, North America (introduced), India	[19,27,61,62,85,91]
10.	*Mesocomys albitarsis* (Ashmead, 1904)	Lepidoptera varia (incl. *Dendrolimus sibiricus* (Lasiocampidae)), Lymantriinae and Saturniidae	Rare	Russia (Primorskiy Territory), China, Korean Peninsula, Japan, southeast Asia	[61,85]

No.	Species of parasitoids[1]	Hosts range of parasitoids[2]	Abundance of parasitoids	Distribution	References[3]
	**Hymenoptera: Pteromalidae**				
11.	*Euneura lachni* (Ashmead, 1887) *	Lepidoptera varia (incl. *Dendrolimus sibiricus* (Lasiocampidae)), Aphididae, Aleyrodidae (Hemiptera), Syrphidae (Diptera); **parasitoids hosts** (*Trichogramma dendrolimi* (Trichogrammatidae), *Pauesia* spp. (Braconidae (Hymenoptera))	No data	Russia (Irkutsk, Sakhalin Island), Europe, Iran, Pakistan, Kyrgyzstan, China, Korean Peninsula, Japan, North America, India	[61,85,95]
12.	*Mesopolobus subfumatus* (Ratzeburg, 1852) *	Lasiocampidae (incl. *Dendrolimus sibiricus*), Gracillariidae and Tortricidae (Lepidoptera), Diprionidae, Pamphiliidae (Hymenoptera); **parasitoids hosts** (Tachinidae (Diptera), Braconidae, Ichneumonidae and Scelionidae (Hymenoptera))	From rare (elsewhere) to abundant (Krasnoyarsk)	Russia (European part, Western, Eastern Siberia, Far East), Europe, China, Japan, N America	[19,47,50,52,56,57,61,85,79]
13.	*Mesopolobus superansi* Yang et Gu, 1995	*Dendrolimus sibiricus*	No data	Northeast China	[81]
14.	*Pachyneuron solitarium* (Hartig, 1838) *	Lepidoptera varia (incl. *Cosmotriche lobulina*, *Dendrolimus kikuchii* Matsumura, *D. pini*, *D. sibiricus*, *D. spectabilis*, *D. superans* (Lasiocampidae)), Coccinellidae (Coleoptera), Asilidae (Diptera), Aphididae, Coccidae, Pseudococcidae, Psyllidae (Hemiptera); **parasitoids hosts** (*Telenomus tetratomus* (Scelionidae); Aphelinidae, Braconidae, Encyrtidae (Hymenoptera))	Mass	Russia (Buryatia, Far East), Europe, Georgia, Kazakhstan, China, Korean Peninsula, Japan, India	[19,50,56,57,61,81,85,95]
		**LARVAL ENDOPARASITOIDS**			
	**Hymenoptera: Braconidae**				
15.	*Aleiodes* (*Aleiodes*) *esenbeckii* (Hartig, 1838) ssp. *dendrolimi* (Matsumura, 1926)	*Dendrolimus pini*, *D. punctatus*, *D. spectabilis*, *D. sibiricus*, *D. superans*, *D. tabulaeformis* Tsai et Liu, *Cosmotriche lobulina* (Lasiocampidae, Lepidoptera)	Abundant (Krasnoyarsk)	Russia (European part), Europe, Turkey, Iran, Afghanistan<br>For ssp. *dendrolimi*: Russia (Western and Eastern Siberia, Far	[19,27,52,53,57,61,85,90,91,92,93]

No.	Species of parasitoids[1]	Hosts range of parasitoids[2]	Abundance of parasitoids	Distribution	References[3]
				East), Mongolia, China Korean Peninsula, Japan	
16.	*Cotesia ordinaria* (Ratzeburg, 1844) (=*dendrolimi* Mats.)	*Dendrolimus pini*, *D. punctatus*, *D. spectabilis*, *D. sibiricus*, *D. superans*, *D. superans albolineatus*, *D. tabulaeformis*, *Macrothylacia rubi*) (Lasiocampidae), *Amata palestinae* Hampson, (Erebidae) (Lepidoptera)	Rare	Russia (European part, Western and Eastern Siberia, south Far East), Europe, Turkey, Israel, Iran, Mongolia, China, Korean Peninsula, Japan	[19,27,47,52,54,56,57,85,90,91,93]
17.	*Cotesia prozorovi** (Telenga, 1955)	*Dendrolimus superans* (Lasiocampidae, Lepidoptera)	No data	Russia (Eastern Siberia)	[85]
18.	*Cotesia rubecula* (Marshall, 1885)	Macrolepidoptera (incl. Pieridae, Plutellidae)	No data	Russia (European part, Eastern Siberia, Far East), Europe, Iran, China, North America, Australia	[85]
19.	*Cotesia rubripes* (Haliday, 1834)	Lepidoptera varia (incl. Geometridae, Lasiocampidae, Noctuidae, Notodontidae and Sphingidae)	Mass 26-70% (Tomsk)	Russia (South European part of Russia, Western, Eastern Siberia, south Far East.), Europe, Morocco, Turkey, Kazakhstan, Mongolia, Korean Peninsula, Japan	[19,56,57,85]
20.	*Glyptapanteles liparidis* (Bouche, 1834) (=Apanteles liparidis)	*Dendrolimus pini*, *D. punctatus*, *D. spectabilis*, *D. sibiricus*, *D. superans*, *D. superans albolineatus*, *D. tabulaeformis*, *Eriogaster lanestri*s (Lasiocampidae), Erebidae, Notodontidae and Noctuidae (Lepidoptera)	Rare (Far East, Eastern Siberia)	Russia (European part, Western, Eastern Siberia, south Far East), Europe, Georgia, Armenia, Azerbaijan, North Africa, Iran, Kazakhstan, Mongolia, China, Korean Peninsula, Japan, India, North America (introduced)	[19,27,50,54,56,57,61,90,91,85,93]
21.	*Meteorus versicolor* (Wesmael, 1835)	*Dendrolimus pini*, *D. spectabilis, D. sibiricus*, *Macrothylacia rubi*, *Malacosoma castrense* (Linnaeus), *M. neustria*, *M. parallela* Staudinger, *Selenephera lobulina* (Lasiocampidae)), Erebidae, Geometridae, Lymantriidae, Noctuidae, Nolidae, Notodontidae, and Thaumetopoeidae	No data	Russia (European part, Ural, Western, Eastern Siberia, Far East), Europe, Transcaucasia, Turkey, Israel, Palestine, Iran, Uzbekistan, Tajikistan, Kazakhstan, Mongolia, China,	[85,93]

No.	Species of parasitoids[1]	Hosts range of parasitoids[2]	Abundance of parasitoids	Distribution	References[3]
		(Lepidoptera)		Korea, Japan, North America	
	**Hymenoptera: Ichneumonidae**				
22.	*Casinaria nigripes* (Gravenhorst, 1829)	Lepidoptera varia (incl. *Dendrolimus superans sibiricus, D. pini* (Lasiocampidae), *Orgyia antiqua, Calliteara pudibunda* (Linnaeus) (Erebidae))	Rare	Russia (European part (Samara), Western, Eastern Siberia (Tomsk, Tyva)), Europe	[19,24,27,91]
23.	*Dusona leptogaster* Holmgren, 1860	Lepidoptera varia (incl. *Dendrolimus sibiricus* (Lasiocampidae))	Rare	Russia (Eastern Siberia (Irkutsk), Far East (South Kuril Is.)), Japan (Honshu)	[52–54,56,57,61,90]
24.	*Dusona tenuis* (Förster, 1868) (=*Campoplex proximus* Forster,1868	*Dendrolimus sibiricus, D. spectabilis* (Lasiocampidae, Lepidoptera)	No data	Russia (European part (Yaroslavl Prov.), Siberia). Europe, Azerbaijan, China, India	[48,61]
25.	*Hyposoter takagii* (Matsumura, 1926)	Lepidoptera (varia)	Abundant	China	[27,47,91]
26.	*Hyposoter validus* (Pfankuch, 1921) (=*Anilasta valida*)	*Dendrolimus sibiricus* (Lasiocampidae, Lepidoptera)	Rare	Russia (Eastern, Western Siberia, Far East (Sakhalin)), Europa (Norway, Germany, Poland)	[19,24,90,93]
27.	*Mesochorus kuwayamae* Matsumura, 1926 *	*Dendrolimus albolineatus, D. sibiricus* (Lasiocampidae, Lepidoptera); **parasitoids hosts** (*Cotesia ordinaria* (Braconidae, Hymenoptera))	No data	Russia (Far East), China, Japan	[47,85,90]
28.	*Opheltes glaucopterus* Linnaeus, 1758 (ssp. *apicalis* Matsumura, 1912) *	*Cimbex* (Cimbicidae, Hym), *Dendrolimus sibiricus* (Lasiocampidae, Lepidoptera) (as hyperparasitoid)	No data	Holarctic	[61,74]
	**LARVAL ECTOPARASITOIDS**				
	**Hymenoptera: Ichneumonidae**				
29.	*Acropimpla didyma* (Gravenhorst, 1829)	Lepidoptera varia (incl. *Dendrolimus pini*, *Euthrix potatoria, Malacosoma neustria* (Lasiocampidae), *Lymantria dispar* (Limantriidae.), *Archanara dissoluta* (Treitschke), *Rhizedra lutosa* (Hübner) (Noctuidae, Lepidoptera)	No data	Russia (European part, Far East), Europe, Turkey, China, Japan	[24,27,73,85,91]
30.	*Acropimpla pictipes* (Gravenhorst, 1829)	Lepidoptera varia (incl. Tortricidae, Oecophoridae, Momphidae, Gelechiidae,	No data	Russia (European part, south of Siberia and Far East), Europe,	[24,27,73,85,91]

No.	Species of parasitoids[1]	Hosts range of parasitoids[2]	Abundance of parasitoids	Distribution	References[3]
		Geometridae), *Anthonomus pomorum* (Linnaeus) (Curculionidae, Coleoptera)		Caucasus, Turkey, Kazakhstan, Mongolia, China, Japan	
31.	*Iseropus stercorator* (Fabricius, 1793)	Lepidoptera varia (incl. Lasiocampidae, Noctuidae, Saturniidae, Sphingidae)	Abundant (Krasnoyarsk)	Russia (European part, Ural, Siberia, Far East), Europe, North Africa, Caucasus, Turkey, Iran, Central Asia, Kazakhstan, Mongolia, China, Japan, North America	[19,24,50,52–54,56,57,61,85,90,91,93]
32.	*Netelia (Netelia) melanura* (Thomson, 1888) (=*Paniscus testaceus* Gravenhorst, 1829)	*Dendrolimus sibiricus* (Lasiocampidae), *Acronicta tridens* Denis et Schiffermuller, *Drymonia ruficornis* (Hufnagel) (Noctuidae, Lepidoptera)	Rare	Russia (European part, Eastern Siberia, Far East), Europe, North Africa, Canary Islands, Georgia, Turkey, Uzbekistan, Japan	[61,85]
		**LARVAL OR LARVAL-PUPAL ENDOPARASITOIDS**			
	**Diptera: Tachinidae**				
33.	*Blepharipa pratensis* (Meigen, 1824) (=*scutellata* R.-D)	Macrolepidoptera varia (incl. *Dendrolimus pini* L., *Dendrolimus superans sibiricus*, *Lymantria dispar* (Lasiocampidae))	From rare to abundant (20%)	Russia (south of Siberia and Far East), Transcaucasia, Europe	[19,22,24,27,56,86,91]
34.	*Blepharipa schineri* (Mesnil, 1939)	Macrolepidoptera varia (incl. *Lymantria dispar*, *Dendrolimus sibiricus* (Lasiocampidae), *Endromis versicolora* (Endromididae), Erebidae, Notodontidae, Lasiocampidae)	From rare to abundant	Russia (European part, south of Siberia and Far East), China, Japan, Europe (to Germany)	[19,22,24,27,57,86,91]
35.	*Blepharipa zebina* (Walker, 1849)	Macrolepidoptera (varia)	No data	Russia (Primorsky Territory), Japan	[27,86,91]
36.	*Carcelia gnava* (Meigen, 1824)	Lepidoptera varia (incl. *Malacosoma neustria* (Lasiocampidae), Lymantriidae, Arctiidae, Notodontidae, Thyatiridae)	Rare	Russia (European part, Far East), Europe, Japan	[22,86]
37.	*Carcelia matsukarehae* (Shima, 1969)	*Dendrolimus superans sibiricus*, *Dendrolimus spectabilis* (Lasiocampidae, Lepidoptera)	Abundant (Amur)	Russia (Primorsky Territory), Japan	[22,24,27,86,91]
38.	*Clemelis pullata* (Meigen,	Lepidoptera varia (incl. *Loxostege sticticalis*	No data	Russia (European part, Western,	[22,26]

No.	Species of parasitoids[1]	Hosts range of parasitoids[2]	Abundance of parasitoids	Distribution	References[3]
	1824)	(Linnaeus) (Crambidae), *Dendrolimus sibiricus* (Lasiocampidae))		Eastern Siberia), Europe, Transcaucasia, Israel, Central Asia, China, Mongolia	
39.	*Compsilura concinnata* (Meigen, 1824)	Lepidoptera varia (incl. *Dendrolimus sibiricus* (Lasiocampidae)), Tenthredinidae (Hymenoptera)	Rare	Russia (central and south European part, Transcaucasia, Europa, Central Asia, Israel, south of Siberia, Japan	[22,24,27,86,91]
40.	*Panzeria rudis* (Fallen, 1810) (=*Ernestia rudis*)	*Dendrolimus sibiricus* (Lasiocampidae), *Panolis flammea* (Denis et Schiffermuller), *Orthosia* spp. (Noctuidae, Lepidoptera)	Up to 100% (on *Dendrolimus pini* and *Lymantria dispar*)	Russia (European part, south of Siberia, Far East), Europe, Japan, Mongolia, Tajikistan	[22,49,86]
41.	*Exorista larvarum* (Linnaeus, 1758)	Lepidoptera varia (incl. *Dendrolimus sibiricus* (Lasiocampidae) and *Lymantria dispar* (Erebidae))	From rare to abundant	Russia (European part, Western, Eastern Siberia, south of Far East), Europe, Middle East, North Africa, Transcaucasia, Central Asia, Mongolia, Japan, India. Taiwan, Nearctic (introduced)	[19,22,24,26,27,52–54,56,61,67,86,91]
42.	*Exorista fasciata* (Fallén, 1820)	Lepidoptera varia (incl. Lymantriidae, Noctuidae, Arctiidae, Zygaenidae)	Rare	Russia (European part, Western, Eastern Siberia, south of Far East), Europe, Transcaucasia, Middle East, Mongolia, China, Taiwan	[19,22,24,27,50,54,56,57,61,86,89,91]
43.	*Exorista segregata* (Rondani, 1859)	Lepidoptera varia (incl. *Dendrolimus sibiricus*)	Rare	Russia (European part, Western Siberia), Europe, Transcaucasia, North Africa, Middle East, Central Asia Mongolia	[22,86,89]
44.	*Hubneria affinis* (Fallén, 1810)	Lepidoptera (varia)		Russia (European part, Eastern Siberia), Europe, Transcaucasia, Mongolia, China	[19,22,26,56,57,86]
45.	*Lespesia frenchii* (Williston, 1889)	Lepidoptera (varia)	Abundant	Russia (Siberia, Far East), North America.	[27,89,91]

No.	Species of parasitoids[1]	Hosts range of parasitoids[2]	Abundance of parasitoids	Distribution	References[3]
46.	*Linnaemya perinealis* Pandellé, 1895	Lepidoptera (varia)	No data	Russia (European part, Siberia), Europe, Kazakhstan, Mongolia, China, Japan	[86,89]
47.	*Masicera sphingivora* (Robineau-Desvoidy, 1830) (= *zimini* Kol.)	Lepidoptera varia (incl. *Dendrolimus sibiricus* (Lasiocampidae), Geometridae, Erebidae, Sphingidae, Noctuidae, Lymantriidae)	From rare (Buryatia) to abundant (other parts of Siberia)	Russia (European, Siberia, south Far East, Western Russia), Europe, Transcaucasia, Central Asia, Japan, Iran, Mongolia	[19,22,27,50,53,54,56,57,61,86,89]
48.	*Macroprosopa atrata* (Fallén, 1810)	Lepidoptera	No data	Russia (European part, Siberia), Europe, Transcaucasia, Mongolia	[89]
49.	*Mikia tepens* (Walker, 1849) (= *M. magnifica*)	Macrolepidoptera varia (incl. *Dendrolimus superans sibiricus, Dendrolimus pini* L., (Lasiocampidae), *Lymantria monacha* (Linnaeus) (Limantriidae))	From rare to abundant	Russia (Western, Eastern Siberia, south of Far East), Europe, Kazakhstan, China, Japan, India, Malaysia, Vietnam	[19,22,24,26,56,57,86,91]
50.	*Pales pavida* (Meigen, 1824)	Macrolepidoptera (varia), some Microlepidoptera.	Rare	Russia (European part, south of Western and Eastern Siberia, south of Far East), Europe, Transcaucasia, Central Asia, Middle East, Mongolia, China, Japan	[19,22,24,26,27,56,57,86,91]
51.	*Picconia incurva* (Zetterstedt, 1844)	Lepidoptera (varia)	No data	Russia (Eastern Siberia), Europe, Transcaucasia, Iran, Israel, Turkmenistan, Mongolia	[89]
52.	*Pseudogonia rufifrons* (Wiedemann, 1830)	Lepidoptera (varia)	No data	Widespread in Palearctic, Oriental, Australasian and Afrotropical regions	[89]
53.	*Senometopia excisa* (Fallén, 1820)	Lepidoptera varia (incl. *Dendrolimus sibiricus, D. superans albolineatus* (Lasiocampidae), Notodontidae, Noctuidae, Geometridae, Nymphalidae)	Rare	Russia (European part, Far East), Europe, Japan	[22,86]
54.	*Tachina grossa* (Linnaeus,	Lepidoptera varia (incl. Lymantriidae,	Rare	Russia (European part, Western,	[19,22,27,50,54,56,57,61

No.	Species of parasitoids[1]	Hosts range of parasitoids[2]	Abundance of parasitoids	Distribution	References[3]
	1758)	Lasiocampidae, Sphingidae)		Eastern Siberia, south Far East (Amur)), Europe, Transcaucasia, C. Asia, Kazakhstan, Mongolia, China	,86,89,91]
55.	*Tachina nupta* (Rondani, 1859)	Lepidoptera (varia)	Rare	Russia (European part, Western, Eastern Siberia, south Far East), Europe, Transcaucasia, Central Asia, China, Mongolia, Japan, Korean Peninsula	[22,27,89,91]
56.	*Thelaira nigripes* (Fabricius, 1794)	Macrolepidoptera varia (incl. *Dendrolimus sibiricus* (Lasiocampidae), Arctiidae, Noctuidae)	Rare	Russia (European part, Western, Eastern Siberia, south Far East), Europe, Transcaucasia, China, Japan, Taiwan	[22,26]
57.	*Tachina magna* (Giglio-Tos, 1890)	Macrolepidoptera varia (incl. Limantriidae, Notodontidae)	Rare	Russia (European part, south Ural, Eastern Siberia, south of Far East), Europe, Transcaucasia, China, Japan, Taiwan	[22,26,56,61,86]
58.	*Winthemia venusta* (Meigen, 1824)	Lepidoptera varia (incl. Notodontidae, Geometridae)	No data	Russia (European part, Eastern Siberia, south Far East), Europe, Transcaucasia, China, Japan, Taiwan	[26,27,86,91]
	**Hymenoptera: Ichneumonidae:**				
59.	*Achaius oratorius oratorius* (Fabricius, 1793)	*Dendrolimus sibiricus, Dendrolimus spectabilis* (Lasiocampidae, Lepidoptera)	No data	Russia (European part of Russia, Siberia, Far East), Europe, Azerbaijan	[47,90]
60.	*Diphyus amatorius* (Müller, 1776)	Lepidoptera varia (incl. *Dendrolimus sibiricus* (Lasiocampidae), Geometridae, Noctuidae)	No data	Russia (European part of Russia, Siberia, south Far East. Europe, Turkey, Azerbaijan, China, Japan	[47,90]
61.	*Habronyx heros* (Wesmael, 1849) (= *gigas* Kriech.)	Lepidoptera varia (incl. *Dendrolimus pini, D. punctatus, D. spectabilis, D. sibiricus, D. superans, D. superans albolineatus, Lasiocampa quercus*	Rare	Russia (European part, south of Siberia and Far East), Europe, Turkey, Israel, China, Korean	[19,27,52,85,91,93]

No.	Species of parasitoids[1]	Hosts range of parasitoids[2]	Abundance of parasitoids	Distribution	References[3]
		(Linnaeus), *Macrothylacia rubi*, *Pachypasa otus* (Druyry) (Lasiocampidae), Noctuidae, Sphingidae)		Peninsula, Japan	
62.	*Lymantrichneumon disparis segmentalia* (Uchida, 1936)	Lymantriidae (Lepidoptera)	No data	Russia (Far East), China, Korean Peninsula, Japan	[90]
63.	*Therion circumflexum* (Linnaeus, 1758)	Lepidoptera varia (incl. *Dendrolimus pini*, *D. spectabilis*, *D. sibiricus*, *D. superans*, *D. superans albolineatus*, *Lasiocampa trifolii* (Lasiocampidae), Erebidae, Geometridae, Noctuidae, Notodontidae and Sphingidae)	No data	Russia (European part, Ural, Eastern Siberia, Far East), Europe, North Africa, Caucasus, Turkey, Israel, Mongolia, China, Korean Peninsula, Japan, India, North America	[47,50,85,93]
64.	*Therion giganteum* (Gravenhorst, 1829)	Lepidoptera varia (incl. *Dendrolimus* spp., *Gastropacha* sp., *Lasiocampa* spp. (Lasiocampidae), *Lycophotia* sp. and *Pseudaletia* sp. (Noctuidae))	From rare (Buryatia) to abundant (Far East)	Russia (south European part, Western, Eastern Siberia, south Far East), Europe, North Africa, Caucasus, China	[19,24,47,52–54,56,57,85,90]
	**LARVAL OR LARVAL-PUPAL ENDOPARASITOIDS**				
65.	*Delomerista mandibularis* (Gravenhorst, 1829) *	*Dendrolimus sibiricus* (Lasiocampidae, Lepidoptera), Tenthredinidae (Hymenoptera); **parasitoids hosts** (*Aleiodes* (*Aleiodes*) *esenbeckii* ssp. *dendrolimi* (Braconidae, Hymenoptera))	Abundant (one of the main secondary parasitoids)	Russia (European part, Ural, south of Western, Eastern Siberia and Far East), Europe, Turkey, Japan, North America	[19,50,51,73,85]
	**Hymenoptera:Eurytomidae**				
66.	*Eurytoma rosae* Nees, 1834 *	Cecidomyiidae, Tephritidae (Diptera), Cynipidae (Hymenoptera), Tortricidae, Arctiidae (Lepidoptera); **parasitoids hosts** (*Aleiodes esenbeckii* ssp. *dendrolimi* (Braconidae, Hymenoptera))	Rare	Russia (European part, Western and Eastern Siberia, Far East), Europe, North Africa, Armenia, Turkey, Iran, Kazakhstan, China, South America	[19,56,57,61,85]
	**Hymenoptera:Torymidae**				
67.	*Monodontomerus aeneus* (Fonscolombe, 1832) (=*obsoletus*) *	Lepidoptera varia (incl. Arctiidae, Lasiocampidae, Lymantriidae, Pieridae, Tortricidae), Apidae, Diprionidae, Pamphiliidae,	Abundant (Krasnoyarsk). One of the	Russia (European part, Western and Eastern Siberia), Europe, Georgia, Azerbaijan, Iran,	[19,50,52,85]

No.	Species of parasitoids[1]	Hosts range of parasitoids[2]	Abundance of parasitoids	Distribution	References[3]
		Sphecidae, Tenthredinidae and Vespidae (Hymenoptera); **parasitoids hosts** (*Therion giganteum, Casinaria nigripes*, *Hyposoter validus* (Ichneumonidae), *Aleiodes esenbeckii* ssp. *dendrolimi* (Braconidae), Chrysididae (Hymenoptera), Tachinidae (Diptera))	most important secondary parasitoids	Kyrgyzstan, Kazakhstan, China, North and South America	
	**Hymenoptera: Eupelmidae**				
68.	*Eupelmus* (*Eupelmus*) *microzonus* Forster, 1860 *	Cynipidae, Eurytomidae (Hymenoptera), Apionidae, Bruchidae, Curculionidae (Coleoptera), Cecidomyiidae, Chloropidae,Tephritidae (Diptera), Lasiocampidae (incl. *Dendrolimus sibiricus*), Psychidae, Pyralidae (Lepidoptera); **parasitoids hosts** (*Aleiodes esenbeckii* ssp. *dendrolimi* (Braconidae, Hymenoptera))	Rare	Russia (European part, Western Siberia, Far East), Europe , North Africa, Caucasus, Turkey, Israel, United Arab Emirates, Iran, Afghanistan, Turkmenistan, Tajikistan, Uzbekistan, Kyrgyzstan, Kazakhstan, China, North America	[19,56,57,85]
69.	*Eupelmus* (*Macroneura*) *vesicularis* (Retzius, 1783) *	Coleoptera, Diptera, Hymenoptera and Lepidoptera; **parasitoids hosts (***Aleiodes esenbeckii* ssp. *dendrolimi* (Braconidae, Hymenoptera))	Rare	Russia (European part, Western Siberia, Far East), Europe, N Africa, Georgia, Armenia, Turkey, Israel, Iran, Turkmenistan, Kazakhstan, China, North America, Australia	[19,56,57,85]
	**Hymenoptera: Perilampidae**				
70.	*Perilampus nitens* Walker, 1834 *	*Dendrolimus sibiricus* (Lasiocampidae, Lepidoptera); **parasitoids hosts** (*Aleiodes esenbeckii* ssp. *dendrolimi* (Braconidae, Hymenoptera), Tachinidae (Diptera))	Rare	Russia (European part, Western Siberia, Far East), Europe	[19,56,85]
	**Hymenoptera: Pteromalidae**				
71.	*Dibrachys microgastri* (Bouche, 1834) *	Araneae, Coleoptera, Dermaptera, Diptera, Hemiptera, Hymenoptera (Diprionidae, Tenthredinidae), Lepidoptera, Neuroptera; **parasitoids hosts** (*Masicera sphingivora* (Tachinidae, Diptera), *Agria affinis, Sarcophaga*	Abundant	Russia (European part, W Siberia, Far East), Europe, N Africa, Turkey, Syria, Iran, Afghanistan, Pakistan, Turkmenistan, Tajikistan, Uzbekistan,	[19,53,57,61,82,85]

No.	Species of parasitoids[1]	Hosts range of parasitoids[2]	Abundance of parasitoids	Distribution	References[3]
		uliginosa, S. pseudoscoparia (Sarcophagidae, Diptera), *Casinaria nigripes, Hyposoter validus, Theronia atalantae* (Ichneumonidae, Hymenoptera), *Aleiodes esenbeckii* ssp. *dendrolimi* (Braconidae, Hymenoptera))		Kyrgyzstan, Kazakhstan, Mongolia, China, Korean Peninsula, Japan, N America, India, SE Asia, Afrotropics, South America, Australasia	
72.	*Pteromalus kuwayamae* Matsumura, 1926 *	*Dendrolimus sibiricus, D. spectabilis* (Lasiocampidae, Lepidoptera); **parasitoids hosts** (*Hyposoter takagii* (Ichneumonidae, Hymenoptera))	No data	Russia: Far East (Sakhalin)	[47]
73.	*Pteromalus matsuyadorii* Matsumura, 1926 *	*Dendrolimus sibiricus, D. spectabilis* (Lasiocampidae, Lepidoptera)	No data	Russia: Far East (Sakhalin)	[47]
	**Hymenoptera: Chalcididae**				
74.	*Brachymeria fiskei* (Crawford, 1910) *	Lepidoptera varia (incl. *Dendrolimus sibiricus* (Lasiocampidae), Lymantriidae, Yponomeutidae); **parasitoids hosts** (*Blepharipa schineri* (Tachinidae, Diptera))	No data	China	[61]
75.	*Brachymeria minuta* (Linnaeus, 1767) *	Lepidoptera varia (incl. Arctiidae, Gelechiidae, Hesperiidae, Lasiocampidae, Lymantriidae, Pieridae, Tortricidae, Yponomeutidae); **parasitoids hosts** (*Masicera sphingivora, Blepharipa pratensis* (Tachinidae, Diptera))	Rare	Russia (European par, Ural, Western and Eastern Siberia, Far East), Europe, North Africa, Turkey, Syria, Israel, Iran, Uzbekistan, Kazakhstan, Japan, India, southeast Asia, Australasia	[19,54,56,57,61,85,90]
	**Hymenoptera:Torymidae**				
76.	*Monodontomerus minor* (Ratzeburg, 1848) (Hymenoptera:Torymidae) *	Apidae, Cimbicidae Diprionidae (Hymenoptera), Lepidoptera, Diptera (Tachinidae); **parasitoids hosts:** (*Masicera sphingivora (=Masicera zimini* Kolomiets, 1952*)* (Tachinidae, Diptera), *Aleiodes esenbeckii* ssp. *dendrolimi* (Braconidae), Ichneumonidae, Eurytomidae (Hymenoptera))	Rare	Russia (European part, Eastern Siberia, South of Far East), Europe, Georgia, Turkey, Pakistan, Tajikistan, Kazakhstan, China, Korean Peninsula, Japan, N America, India	[19,85]

No.	Species of parasitoids[1]	Hosts range of parasitoids[2]	Abundance of parasitoids	Distribution	References[3]
		**PUPAL ENDOPARASITOIDS**			
	**Hymenoptera: Ichneumonidae**				
77.	*Apechthis capulifera* (Kriechbaumer, 1887)	Lepidoptera varia (incl. Geometridae, Saturniidae, Lasiocampidae, Libytidae, Lymantriidae, Lycaenidae, Noctuidae, Pieridae, Papilionidae, Satyridae, etc.)	Abundant	Russia (South of Siberia and Far East), Europe, Caucasus, Central Asia, China, Korean Peninsula, Japan	[24,27,73,85,91]
78.	*Apechthis quadridentata** (Thomson, 1877) (=*Pimpla dendrolimi* Mats.)	Lepidoptera varia (incl. *Dendrolimus superans*, *Dendrolimus spectabilis* (Butler) (Lasiocampidae))	Rare	Russia (European part, Ural, south of Siberia and Far East) Europe, Caucasus, Turkey, Kazakhstan, Mongolia, China, Korean Peninsula, Japan	[47,61,73,85]
79.	*Apechthis compunctor* (Linnaeus, 1758)	Lepidoptera varia (incl. Geometridae, Pieridae, Lasiocampidae, Lymantriidae)	Rare	Russia (European part, south of Siberia and Far East) Europe, Caucasus, Turkey, Central Asia, Kazakhstan	[19,56,73,85]
80.	*Gregopimpla himalayensis* (Cameron, 1899) (= *Iseropus himalayensis*	Lepidoptera varia (incl. *Margaronia pyloalis* Wlk. (Pyralidae), *Dendrolimus punctatus*, *D. spectabilis*, *Malacosoma neustrium*, *Gastropacha quercifolia* Linnaus (Lasiocampidae), *Lymantria dispar* (Lymantriidae), *Clostera anastomosis* Linnaeus (Notodontidae), *Grapholita molesta* Busck, *Leguminivora glycinivorella* Matsumura, *Archips oporanus* Linnaeus (Tortricidae), *Ectomyelois pyrivorella* Matsumura (Pyralidae)	No data	Russia (Far East), China, Korean Peninsula, Japan, India	[27,73,85,90,91]
81.	*Itoplectis tabatai** (Uchida, 1930)	*Dendrolimus superans*, *Dendrolimus sibiricus* (Lasiocampidae, Lepidoptera)	Rare	Russia (Far East)	[61,73,85]
82.	*Pimpla disparis* Viereck, 1911	Lepidoptera varia.	Rare; can be abundant and effective	Russia (Eastern Siberia, Far East), Mongolia, China, Korean Peninsula, Japan, North America (introduced), India	[27,61,85,91]

No.	Species of parasitoids[1]	Hosts range of parasitoids[2]	Abundance of parasitoids	Distribution	References[3]
83.	*Pimpla pluto* Ashmead, 1906	Lepidoptera varia (inc. *Dendrolimus sibiricus*)	Rare	Russia (Western and Eastern Siberia, Far East), Kazakhstan, China, Korean Peninsula, Japan	[61,85]
84.	*Pimpla rufipes* (Miller, 1759) (*Pimpla instigator* F.)	Lepidoptera (varia)	From rare to abundant (Siberia)	Russia (European part, Ural, Western and Eastern Siberia, Far East), Europe, North Africa, Caucasus, Turkey, Iran, Afghanistan, Central Asia, Kazakhstan, Mongolia, China, Korean Peninsula, Japan, North America (introduced)	[19,27,52,54,56,61,85,90,91]
85.	*Pimpla turionellae* (Linnaeus, 1758) *	Lepidoptera varia (incl. Choreutidae, Erebidae, Geometridae, Tortricidae, Yponomeutidae etc.); **parasitoids hosts** (*Dusona leptogaster* (Ichneumonidae, Hymenoptera))	Rare	Russia (European part, Ural,W, S Siberia, Far East), Europe, N Africa, Caucasus, Turkey, Israel, Iran, Afghanistan, Central Asia, Kazakhstan, Mongolia, China, Korean Peninsula, Japan, North America (introduced), India	[19,57,85,90]
86.	*Theronia atalantae gestator* (Thunberg, 1822)	Lepidoptera varia (incl. *Dendrolimus sibiricus*, *D. superans*, *D, spectabilis*, *D punctatus*, *D. tabulaeformis*, *Limantria dispar* (Limantriidae), *Hyphantria cunea* (Erebidae), *Pieris rapae* (Pieridae))	No data	Russia (Eastern Siberia, Far East), China, Korean Peninsula, Japan, India	[24,85,90]
87.	*Theronia atalantae atalantae* (Poda, 1761) *	Lepidoptera (varia); **parasitoids hosts** (*Therion giganteum*, *Habronyx heros*, *Hyposoter validus*, *Casinaria nigripes*, *Iseropus stercorator*, (Ichneumonidae), *Aleiodes esenbeckii* ssp. *dendrolimi* (Braconidae) (Hymenoptera), *Mikia tepens* (Tachinidae, Diptera))	From rare (Buryatia) to abundant	Russia (European part, Siberia), Europe, Caucasus, Turkey, Iran, Turkmenistan, North America	[19,24,38,50,53,56,57,61,85]
	**Hymenoptera: Eulophidae**				
88.	*Aprostocetus xanthopus* (Nees, 1834)	Lasiocampidae (incl. *Dendrolimus superans sibiricus*), Lymantriidae (Lepidoptera), Scolytinae	Rare (Siberia)	Russia (European part, Eastern Siberia, Far East), Europe	[19,54,56,57,61,85]

No.	Species of parasitoids[1]	Hosts range of parasitoids[2]	Abundance of parasitoids	Distribution	References[3]
		(Coleoptera)			
		**PUPAL ECTOPARASITOIDS**			
89.	*Glyphicnemis profligator* (Fabricius, 1775)	*Dendrolimus sibiricus* (Lasiocampidae, Lepidoptera), *Diprion pini*, *Neodiprion sertifer* (Geoffroy) (Diprionidae), *Nematinus* spp. (Tenthredinidae) (Hymenoptera)	Rare	Russia (European part, Western Siberia, Far East), Europe, Japan	[38,61,90]
90.	*Gelis dendrolimi* (Matsumura, 1926)	*Dendrolimus sibiricus* (Lepidoptera Lasiocampidae)	No data	Russia (Sakhalin), Japan (Shikoku)	[47,90]
91.	*Mesoleptus transversor* Thunberg, 1822 (=*Exolytus splendens* (Grav.)) *	Diptera, Lepidoptera (Lasiocampidae), Hymenoptera (Tenthredinidae); **parasitoids hosts:** *Sarcophaga* (*Robineauella*) *pseudoscoparia* (Kramer), *Sarcophaga uliginosa* (Kramer) (Sarcophagidae, Diptera)	Rare	Russia (Eastern Siberia), Europe	[19,38,54,56,61]
92.	*Phygadeuon subspinosus* Gravenhorst, 1829 (=*Ph. grandiceps* Thoms.) *	Muscomorpha (Diptera); **parasitoids hosts** (*Masicera sphingivora* (Tachinidae, Diptera))	Rare	Russia (European part), Far East, Europe	[19,38,57,90]
93.	*Caenocryptus sexannulatus* Gravenhorst, 1829 *	*Cydia pomonella* (Linnaeus) (Tortricidae), *Dendrolimus sibiricus* (Lasiocampidae) (Lepidoptera); **parasitoids hosts** (*Aleiodes esenbeckii* ssp. *dendrolimi* (Braconidae, Hymenoptera))	From rare (Europe) to abundant (Russia)	Russia (Caucasus, Western Siberia), Europe	[19,38,56,57]

Remarks: [1]hyperparasites; [2]Some parasitoids, which are associated with *D. sibiricus*, can also act as hyperparasites (attacking primary the parasitoids of *D. sibiricus*). For such parasitic insects, additional data on their hosts among parasitoids are provided, indicated under **parasitoids hosts**. [3]See the reference list in our paper.
