# Supplementary material for: The Diversity of Parasitoids and Their Role in the Control of the Siberian Moth, Dendrolimus sibiricus (Lepidoptera: Lasiocampidae), a Major Coniferous Pest in Northern Asia
Source: Life (Basel). 2024 Feb 17;14(2):268. doi: 10.3390/life14020268 (PMC10890493; doi:10.3390/life14020268)
Supplement: Supplementary file 1 [file life-14-00268-s001.zip › Table S4.pdf]

The Parasitoid Diversity and Their Role in the Control of the Siberian Moth, *Dendrolimus sibiricus* (Lepidoptera: Lasiocampidae), a Major Coniferous Pest in Northern Asia

Natalia I. Kirichenko, Alexander A. Ageev, Sergey A. Astapenko, Anna N. Golovina, Dmitry R. Kasparyan, Oksana V. Kosheleva, Alexander V. Timokhov, Ekaterina V. Tselikh, Evgeny V. Zakharov, Dmitrii L. Musolin, Sergey A. Belokobylskij

Table S4. The predatory flies (with some exhibiting parasitoid behavior) associated with larvae and/or pupae of *Dendrolimus sibiricus* in Northern Asia.

No.	Species of parasitoids	Hosts range of insects	Abundances of parasitoids	Realm, country	References[1]
		**DIPTERA: Sarcophagidae**			
1	*Agria affinis* (Fallén, 1817)	Lepidoptera (varia), Diprionidae (*Diprion* spp., *Empria* spp.) (Hymenoptera)	From being rare to common. Heavily infested with secondary parasites.	Holarctic	[19,22,24,27,50,52–54,56,57,61,88,91]
2	*Agria monachae* (Kramer, 1908)	Lepidoptera varia (incl. *Dendrolimus pini*, *D. sibiricus* (Lasiocampidae), *Lymantria dispar*, *L. monacha* (L.) (Erebidae))	Abundant	Russia (European part, south of Siberia, south Far East) Europe, Japan	[22,24,27,88,91]
3	*Sarcophaga* (*Kramerea*) *schuetzei* Kramer, 1909	Macrolepidoptera varia (incl. *Aporia crataegi* (Pieridae), *Euthrix potatoria*, *Dendrolimus pini*, *D. superans sibiricus*, *Cosmotriche lobulina* (Lasiocampidae), *Caligula japonica* (Saturniidae), *Lymantria dispar*, *L. monacha*, *Orgyia antiqua* (Erebidae)	Rare	Russia (European part, south Siberia, Far East), Near East, Transcaucasia, Eastern and Central Europa, Kazakhstan, Mongolia, China, Korean Peninsula, Japan, Oriental Region	[19,24,27,50,54,56,57,61,88,91]
4	*Sarcophaga* (*Pandelleisca*) *similis* Meade, 1876	*Dendrolimus pini* (Lasiocampidae), *Lymantria monacha* (Erebidae), *Lacanobia oleraceae* (Linnaeus) (Noctuidae) (Lepidoptera)	No data	Russia (European part, Siberia, Far East), Europe, Near and Middle East, China, Korean Peninsula, Japan, Oriental Region	[24,27,88,91]

No.	Species of parasitoids	Hosts range of insects	Abundances of parasitoids	Realm, country	References[1]
5	*Sarcophaga* (*Parasarcophaga*) *albiceps* (Meigen, 1826)	Varia Lepidoptera and Coleoptera, Pamphiliidae (Hymenoptera)	Rare (Siberia)	Russia (European part, south of Siberia and Far East), Europe, Europe, Transcaucasia, Near and Middle East, China, Korean Peninsula, Japan. Australian and Oriental Regions	[19,22,47,54,56,57, 61,88,91]
6	*Sarcophaga* (*Robineauella*) *pseudoscoparia* (Kramer, 1911)	Macrolepidoptera varia (incl. *Aporia crataegi* (Linnaeus) (Pieridae), *Euthrix potatoria* (Linnaeus), *Dendrolimus pini*, *D. superans sibiricus*, *Cosmotriche lobulina* (Lasiocampidae), *Caligula japonica* (Saturniidae), *Lymantria dispar*, *L. monacha*, *Orgyia antiqua* (Erebidae)	Abundant, up to 24%	Russia (European part, Siberia, Far East) Central and Eastern Europe, Mongolia, China, Korean Peninsula, Japan	[19,22,24,27,50,54, 56,57,61,88,91]
7	*Sarcophaga (Varirosellea) uliginosa* (Kramer, 1908)	Macrolepidoptera varia (incl. *Aporia crataegi* (Pieridae), *Euthrix potatoria*, *Dendrolimus pini*, *D. superans sibiricus*, *Cosmotriche lunigera* (Lasiocampidae), *Caligula japonica* (Saturniidae), *Lymantria dispar*, *L. monacha*, *Orgyia antiqua* (Erebidae), *Nymphalis xanthomelas* (Esper) (Nymphalidae) and etc.)	From rare to abundant	Russia (European part, Siberia, Far East) Europe, Transcaucasia, Tajikistan, Kazakhstan, Mongolia, China, Korean Peninsula, Japan, Nearctic (introduction)	[19,22,24,27,54,56, 57,61,88,91]
8	*Sarcophaga carnaria* (Linnaeus, 1758)	Lepidoptera varia	No data	Russia (European part, Western Siberia), Near East, Transcaucasia, Europa.	[22,24,27,88,91]
9	*Sarcophaga harpax* Pandellé, 1896 (*Liosarcophaga harpax* Pandelle, 1896)	Macrolepidoptera varia (incl. *Aporia crataegi* (Linnaeus) (Pieridae), *Euthrix potatoria* (Linnaeus), *Dendrolimus pini*, *D. sibiricus* (Lasiocampidae), *Caligula japonica* (Moore) (Saturniidae), *Lymantria dispar*, *L. monacha*, *Orgyia antiqua* (Erebidae))	Rare	Russia (European part, south of Siberia, Far East), Europe, Tajikistan, Kazakhstan, Mongolia, China, Korean Peninsula, Japan, Oriental Region	[19,22,24,27,54,56, 57,88,91]

No.	Species of parasitoids	Hosts range of insects	Abundances of parasitoids	Realm, country	References[1]
		**DIPTERA: Muscidae**			
10	*Muscina stabulans* (Fallén, 1817)	Macrolepidoptera varia (incl. *Dendrolimus superans sibiricus, Dendrolimus pini*, *Malacosoma americanum* (Fabricius) (Lasiocampidae), *Papaipema nebris* (Guenée), *Archanara subcarnea* Kellicott (Noctuidae)), *Leptinotarsa decemlineata* (Say), *Pissodes strobi* (Peck) (Coleoptera)	From rare to abundant	Cosmopolitan	[19,22,24,27,54,56, 57,61,91]
11	*Muscina levida* (Harris, 1780) *(=assimilis* Fallen*)*	*Dendrolimus sibiricus* (Lasiocampidae), *Ectropis crepuscularia* (Denis et Schiffermuller) (Geometridae) (Lepidoptera)	Rare	Cosmopolitan	[19,22,56,57,61]

[1]See the reference list in our paper.
