# Supplementary material for: The Diversity of Parasitoids and Their Role in the Control of the Siberian Moth, Dendrolimus sibiricus (Lepidoptera: Lasiocampidae), a Major Coniferous Pest in Northern Asia
Source: Life (Basel). 2024 Feb 17;14(2):268. doi: 10.3390/life14020268 (PMC10890493; doi:10.3390/life14020268)
Supplement: Supplementary file 1 [file life-14-00268-s001.zip › Table S5.pdf]

The Parasitoid Diversity and Their Role in the Control of the Siberian Moth, Dendrolimus sibiricus (Lepidoptera: Lasiocampidae), a Major Coniferous Pest in Northern Asia

Natalia I. Kirichenko, Alexander A. Ageev, Sergey A. Astapenko, Anna N. Golovina, Dmitry R. Kasparyan, Oksana V. Kosheleva, Alexander V. Timokhov, Ekaterina V. Tselikh, Evgeny V. Zakharov, Dmitrii L. Musolin, Sergey A. Belokobylskij

Table S5. The matrices of Z-values calculated with the use of the Mann-Whitney U-test for comparing the parasitism rates of *Dendrolimus sibiricus* eggs, larvae, and pupae at different population dynamics phases in Northern Asia*.

I. EGG

**The population dynamics phase of *D. sibiricus***	**The population dynamics phase of *D. sibiricus***			
	Growth	Outbreak	Decline	Depression
Growth				
Outbreak	−2.51**			
Decline	−2.66**	−1.98**		
Depression	−0.84	0.88	1.57	

II. LARVA

**The population dynamics phase of *D. sibiricus***	**The population dynamics phase of *D. sibiricus***			
	Growth	Outbreak	Decline	Depression
Growth				
Outbreak	0.38			
Decline	0.01	1.07		
Depression	0.22	1.07	0.7	

III. PUPA

**The population dynamics phase of *D. sibiricus***	**The population dynamics phase of *D. sibiricus***			
	Growth	Outbreak	Decline	Depression
Growth				
Outbreak	−1.06			
Decline	−2.23**	−2.01**		
Depression	−2.32**	−1.77	0.82	

Remarks: *Original data used for the analyses are given in Table S2. **The Z-values indicated with two asterisks are statistically significant at $p < 0.05$; in other cases $p > 0.05$.
